# Bubble-assisted cleaning for primary amine oxidation towards ampere-level current densities

**DOI:** 10.1038/s41467-026-74878-0

**Published:** 2026-06-26

**Authors:** Xichang Lin, Bo Li, Yisong Li, Anuj Kumar, Bin Ding, Jiazhong Wu, Liang Luo, Xiaoming Sun

**Affiliations:** 1 State Key Laboratory of Chemical Resource Engineering, Beijing University of Chemical Technology, Beijing, China; 2 Research Institute of Petroleum Exploration & Development, China National Petroleum Corporation, Beijing, China; 3 State Key Laboratory of Enhanced Oil & Gas Recovery, Beijing, China; 4 Key Laboratory of Oilfield Chemistry (KLOC), CNPC, Beijing, China; 5 Nano-Technology Research Laboratory, Department of Chemistry, GLA University, Mathura, Uttar Pradesh India

**Keywords:** Electrocatalysis, Energy, Electrocatalysis

## Abstract

Organic molecular oxidation reactions are ideally regarded to be energy-saving alternative to replace oxygen evolution reaction (OER) for lowering the oxidation overpotential during green hydrogen production. However, the vast scale of hydrogen production is far beyond the fine chemical processes, inevitably resulting in substantial oxygen release. Here, we propose another type of coupled oxidation reactions to stably accommodate with high-current density hydrogen production, accompanied by easy separation. As taking benzylamine oxidation reaction (BOR) as a probe without other side reactions, we propose a bubble-assisted strategy by activating OER and utilizing the generated oxygen bubbles to timely take the insoluble product benzonitrile away from electrode surface, and effectively overcome the electrode poisoning issue. The assembled prototype water electrolyzer achieves a typical current density of 200 mA/cm^2^ and faradaic efficiency of 55% at 1.65 V for over 40 hours, diversify high-value chemical outputs. Such hybrid electrolysis within bubble-assisted strategy endows system flexibility for next-generation integrated electrochemical energy platforms.

## Abstract (web summary)

Organic oxidation paired with H₂ production is promising yet limited by electrode fouling from insoluble byproducts. Here, the authors report an in-situ O_2_ bubble cleaning strategy to remove oily products and sustain high current densities.

## Introduction

As the world pivots toward sustainable energy, green hydrogen is quickly emerging as a centerpiece for decarbonizing the economy of the 21st century that addresses both climate change mitigation through energy security, but the OER remains the rate-limiting step of overall water electrolysis because of its high thermodynamic potential of 1.23 V vs. reversible hydrogen electrode (RHE) and the lack of product value. Hence, new efforts that aim to couple processes with value-added organic oxidations or develop new catalyst systems to be operando, and potentially to utilize systems engineering to improve energy effectiveness and economic feasibility, will undoubtedly help to shift energy processes for the production of H_2_^1^. Alternative value-added anodic reactions with low thermodynamics have been constantly proposed to couple with hydrogen production^2–5^, as electrochemical hydrogen and chemical cogeneration (EHCC)^6^. However, their industrial feasibility remains constrained. One of the bottlenecks is the rather limited low anodic current density, which reversely compromises hydrogen production and overall system efficiency. In addition, it is worth noting that many reactions often refer to insoluble or slightly soluble species; considering the toxicity and environmental concerns on hydrotropic agents (e.g., acetonitrile)^7,8^, such solubility issues still matter in aqueous electrolytes.

To this end, various strategies have been proposed, basically referring to surface structure construction and modification^9^. For the enrichment of insoluble or limited soluble reactant species on electrodes, surfactants such as SDS^10^, can be integrated to promote the adsorption. Similar methods have also found applications in organic electroreduction, such as the use of Pickering emulsions to achieve electro-hydrogenation processes^11^. While for the reactions with soluble precursors converting into insoluble products, such as Kolbe reactions^12^ and the oxidation of primary amines^13–16^, the adhesion of insoluble oily products usually leads to rapid catalyst deactivation and performance degradation. To overcome this issue, an effective super-oleophobic micro/nano-structured electrode surface was proposed to lower the adhesion of insoluble product and maintain the active sites of the electrode surface for the Kolbe reaction^17^. While the irreversible oil wetting of the electrode surface still tends to happen owing to the formation of oil nanodroplets during the ongoing operation. Hence, the limited current density (< 100 mA/cm^2^) still remains a big gap to couple with industrial water electrolysis conditions^18,19^. Moreover, as compared with projected hydrogen production volumes exemplified by the anticipated 49 Mtpa by 2030, such paired anodic electrolysis remains far behind for the demand^20^. As a result, the rational design of scalable reactors requires matched industrial current density and system flexibility.

From the perspective of surface tension, the oil/gas interface has a much lower value (20 ~ 70 mN/m in room temperature) than that of the water/gas interface ( ~ 75 mN/m in room temperature)^21,22^, the bubble/oil interface has much higher affinity than the bubble/water interface. Hence, bubbles have been widely applied in flotation^23^ and cleaning of porous substrates^24^. In this work, we utilize BOR as one of the model reactions to illustrate an electrochemically generated oxygen bubble-assisted self-cleaning method in which the dynamically generated oxygen bubbles form frizzled bits of product (the insoluble benzonitrile (BN)) from the electrode surface, and the side reaction, hydrogen, can be co-produced in the presence of the bubbles, reducing catalyst fouling over time. The in-situ/operando observations show that bubble-driven detachment eliminates the possibility of catalyst deactivation that was otherwise irreversible. The hybrid device demonstrates industrially relevant performance, delivering 200 mA/cm^2^ at 1.65 V with an observed 55% faradaic efficiency, and stably operates for 40 h with essentially no decay in potential. Such a bubble-assisted strategy can be extended to different primary amine oxidation reactions, demonstrates a framework for a modular and add-on retrofittable value-added platform with techno-economic analysis (TEA), and provides increased electron economy, flexibility, and scalability of the process, which would facilitate the deployment of next-generation integrated electrochemical energy systems.

## Results and discussion

### FeNiS/NF electrocatalyst for BOR

**Fig. 1:**
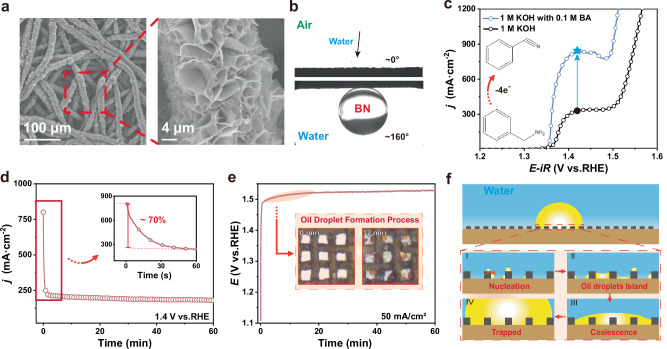
Electrode deactivation caused by oily product accumulation via characterizations. **a** SEM image and the contact angle of the NiFeS catalyst coating on nickel felt. **b** The water droplet contact angle and the underwater oil droplet contact angle. **c** Polarization curves of NiFeS catalyst in 1 M KOH with and without 0.1 M BA. The difference between the two markers represents the increase of current density in the presence of BOR (with 100% *iR* correction and *R*_s_ = 0.16 Ω for 1 M KOH with 0.1 M BA and 0.19 Ω for 1 M KOH. The non-*iR* corrected voltammograms are provided in Supplementary Fig. 21). **d** The stability test of NiFeS catalyst in 1 M KOH with 0.1 M BA. Inset: the decline of stability within the first 50 s (without *iR* correction). **e** Chronoamperometry test at the current density of 50 mA/cm^2^. Inset: the images of the electrode surface from 0 min to 12 min (without *iR* correction). **f** Schematic illustration of oil trapped process on the electrode surface. Source data for (**c–e**) are provided as a Source Data file.

For BOR, NiFeS was chosen as a model catalyst due to its synergistic multi-metallic nature, tunable electronic structure, and demonstrated catalytic activity for coupled electrochemical processes. The catalyst was prepared via the hydrothermal method in which nickel felt was reacted with precursors of Na_2_S and FeCl_3_ for 12 h, leading to the direct growth of well-aligned nanosheets adhered to the conductive substrate. This architecture would result in a durable electrode with mechanical stability, promote fast charge transfer, and provide abundant exposure of the catalytically active sites. Morphological characterization by Scanning Electron Microscope (SEM) showed that the plated nanosheets formed vertically and had grown over the entire surface of the nickel felt, while Energy Dispersive X-ray Spectroscopy (EDS) mapping (Fig. 1a, Supplementary Fig. 1) confirmed that Ni, Fe, and S were uniformly distributed throughout the nanosheet structures. Crystallographic evidence from X-ray diffraction (XRD, Supplementary Fig. 2) revealed diffraction patterns indexed to Ni_3_S_2_ (PDF:44-1418) and FeNi_3_ (PDF:38-0419), as confirmed by the presence of S in the FeNi_3_ alloy matrix^25^. The lattice incorporation of S is relevant because doping S into the alloy would regulate the electronic state of Ni and Fe centers, facilitating electron delocalization and effectively tuning the adsorption–desorption energetics of intermediates involved in the BOR. To further clarify the surface chemical states, we performed X-ray photoelectron spectroscopy (XPS, Supplementary Fig. 3). The characteristic peaks at 855.3 eV and 873.5 eV were consistent with Ni^2+^ in the Ni 2*p* region, while the S 2*p* spectrum had peaks at 163.6 eV and 168.5 eV, referencing metal sulfide and surface sulfate formed on the sulfide surface, respectively. The Fe 2*p* region showed peaks at 711.0 eV and 724.5 eV consistent with Fe^3+^, confirming that Fe has been successfully incorporated into the Ni_3_S_2_ lattice. Together, this spectroscopic evidence confirmed the construction of a multicomponent NiFeS framework with electronically modulated active sites for concerted electro-oxidation processes. In addition to structural and compositional benefits, the nanosheet morphology provides a high surface-to-volume ratio and interconnective porosity, which are essential for optimizing electrolyte diffusion and availability to the reactants. Moreover, the altered surface chemistry materially affects the wettability, which is increasingly viewed as a key factor impacting catalytic activity in electrochemical systems. We derived the principle based on Young’s equation (see more details in Supplementary Information Note 1)

1 $$\cos {\theta }_{{og}}=\frac{{\gamma }_{{og}}\cos {\theta }_{{og}}-{\gamma }_{{wg}}\cos {\theta }_{{wg}}}{{\gamma }_{{ow}}}$$

where ${\gamma }_{{og}}$, ${\gamma }_{{wg}}$ and ${\gamma }_{{ow}}$ are the surface tension of oil-gas interface, water-gas interface and oil-water interface, respectively. Hence, in principle, there is a strong inverse relationship between hydrophobicity and hydrophilicity^26^. The wettability of the as-prepared electrode was investigated. Generally, the solid-gas interfacial tension is lower than the solid-liquid interfacial tension. The electrode surface can demonstrate similar wettability. The water droplet contact angle of ~0° indicates the superhydrophilicity of the electrode, and the product (BN) oil droplet contact angle presents ~0° (Supplementary Fig. 4). However, the underwater oil droplet contact angle exceeds 160°, presenting superoleophobic nature (Fig. 1b). This initial superoleophobicity is expected for managing the organic product (Supplementary Fig. 5). The as-prepared NiFeS@nickel felt catalyst exhibits an OER onset potential at approximately 1.5 V vs. RHE in 50 mL 1 M KOH, with a pre-oxidation peak of Ni active species (Ni^2+^ to Ni^3+^) at 1.35 V (Fig. 1c). With the increase of added concentrations of Benzylamine (BA) precursor (Supplementary Fig. 6), the current density of polarization curve increases accordingly, and can even reach up to 850 mA/cm^2^ for 0.1 M BA at 1.42 ± 0.04 V vs. RHE (corresponding to the oxidation potentials of BOR), while that of bare nickel felt or nickel foam are both only 75.7 mA/cm^2^, indicating the ideal activity of NiFeS catalyst (Supplementary Fig. 7). Besides, the NiFeS catalyst grown on nickel foam exhibits a similar performance trend, delivering a current density of 450 mA/cm^2^ in 0.1 M BA at 1.42 ± 0.04 V vs. RHE, compares to 75.0 mA/cm^2^ of bare substrate (Supplementary Fig. 8). GC-MS analysis confirms that no detectable organic by-products, with average faradaic efficiency over 95% for BOR (Supplementary Figs. S9–S12).

However, different from the linear sweep voltammetry (LSV), the current density under the constant potential of 1.40 V vs. RHE decreases significantly from 800 mA/cm^2^ to 230 mA/cm^2^ within the first 50 s in each cycle, representing a decrease of over 70% (Fig. 1d). To elucidate the reason for the rapid performance degradation, we investigated the electrochemical surface area (ECSA) measurements. As shown in Supplementary Fig. 13, the ECSA of the electrode is initially 34.75 cm^2^/cm^2^, but decreases to 31.5 cm^2^/cm^2^ after operating at 1.40 V vs. RHE for 10 min. Notably, when the reaction is conducted at a higher potential of 1.5 V vs. RHE for 10 min, the ECSA recovers to 33.25 cm^2^/cm^2^ to some extent. For the verification of the phenomenon, we selected a simple nickel mesh as the substrate, and the working potential increases similarly and rapidly under constant current density measurement for the NiFeS catalyst coated on nickel mesh (NiFeS@NM) (Fig. 1e). NiFeS@NM also exhibits superoleophobic properties underwater (Supplementary Fig. 14). To enhance visualization of the colorless oily BN product and bubbles, we pre-stained the NM substrate by immersing it in an Oil Red O ethanol solution, followed by drying (Supplementary Figs. 15, 16). As compared with the clean state at the initial stage, the NiFeS@NM catalyst surface is obviously encapsulated by a large amount of oily BN products (with oil red O adsorbed) from 0 to 12 min (Fig. 1e inset). Hence, the dramatic performance decline can be attributed to the accumulation of oily BN product on the active sites. We can essentially infer that the oily product generated would form as a tiny nucleus, gradually become trapped as an oil island between the nanoarrays, and finally turn into an oil film (Fig. 1f), even though the electrode is initially super-oleophobic underwater.

### Observation of bubble-assisted behaviors for BOR

**Fig. 2:**
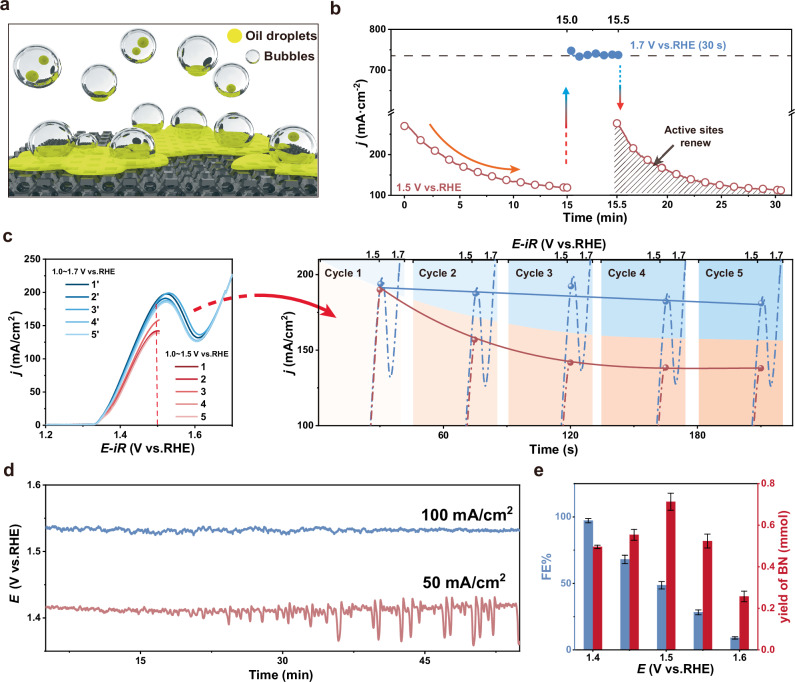
Effect of in-situ oxygen bubbles induced oily product removal on catalytic stability and current density. **a** Schematic illustration of bubble-assisted removal of oily product on the electrode surface. **b** Surface renewal via high-potential pulse enhances current stability (without *iR* correction). **c** Multi-step LSV test and current density peaks at different scan ranges with a scan rate of 10 mV/s (with 100% *iR* correction and *R*_s_ = 0.10 Ω. The non-*iR* corrected voltammograms are provided in Supplementary Fig. 21). **d** Potential variations under constant current densities of 50 mA/cm^2^ and 100 mA/cm^2^(without *iR* correction). **e** Histogram of Faraday efficiency (FE) and yield under different potentials in 1 M KOH with 0.1 M BA (without *iR* correction). Error bars represent standard deviation (*n* = 3). Source data for (**b–e**) are provided as a Source Data file.

Due to the significant accumulation of oily products, we propose a hybrid electrolysis method that utilizes an oxygen bubble-assisted approach for their removal (Fig. 2a). Owing to the strong affinity between the bubble and oily product, the generated BN would be effectively removed along with oxygen bubble detachment. Constant-voltage operation experiments under different conditions were conducted to verify the feasibility of this strategy (Fig. 2b). There is a significant current decay from the beginning at 1.5 V, while switching the potential to 1.70 V as a pulse for 30 s to activate OER, the current density can immediately recover from 120 mA/cm^2^ to 280 mA/cm^2^, and decays again as it switches to 1.50 V. Similarly, the variations of multi-cycle polarization curves also prove the roles of OER activation (Fig. 2c). For the LSV range from 1.0 to 1.50 V, the current density gradually decreases from 192 mA/cm^2^ of the first cycle to 140 mA/cm^2^ of the fifth cycle. When the LSV potential window is shifted from 1.50 V to 1.70 V, which activates the competitive OER, the current density at 1.50 V remains stable throughout all five cycles. Further, as shown in Fig. 2d, the potential corresponding to the constant current density of 100 mA/cm^2^ remains rather stable at 1.54 V for 60 min but exhibits progressive fluctuations around approximately 1.42 V at the current density of 50 mA/cm^2^, particularly with prolonging time. The FE and yield of BOR under varied voltages were further investigated using the NiFeS@nickel mesh electrode (1 cm^2^) in a three-electrode cell. The results indicate that the catalyst achieves a nearly 100% FE at a potential of 1.40 V, with a yield of 0.55 mmol/h (Fig. 2e). As the anodic potential increases, the OER becomes increasingly competitive, leading to a decrease in FE of BOR. The yield of BN rises gradually from 0.55 mmol/h to 0.71 mmol/h as the potential reaches 1.50 V, corresponding to an FE of 48.6%, reflecting competing OER activation. Beyond 1.55 V, the dominant OER decreases both BN yield and FE.

**Fig. 3:**
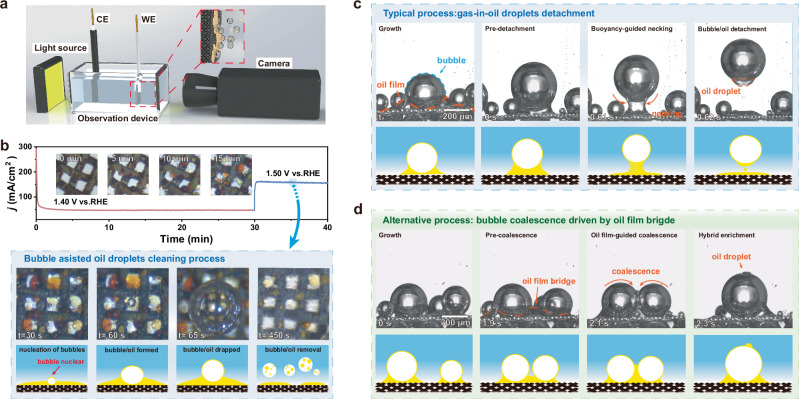
In-situ observations of bubble-assisted oily product removal from different views. **a** Configuration of observation setup using a CCD camera. **b** Two-step constant voltage test at 1.40 V vs. RHE and 1.50 V vs. RHE. And the inset shows the growth of an oil droplet captured by a CCD, and the bubble-assisted oil droplets cleaning process with its schematic diagrams in bule dash box (without *iR* correction). **c** Illustrations and the corresponding schematic diagrams of gas-in-oil droplets detachment. **d** Illustrations and the corresponding schematic diagrams of the bubble coalescence process between two reaction sites by oil film bridge. Source data for (**b**) is provided as a Source Data file.

To clearly investigate the bubble effect, we conducted the in-situ monitoring of the variations of electrode surface using a high-speed camera (Fig. 3a). As expected, the current density gradually declines after the initially sharp drop at a constant potential of 1.40 V vs. RHE for 30 min (Fig. 3b), accompanied by the BN oil droplets accumulated on the electrode surface (Fig. 3b inset). When the potential is switched to 1.50 V vs. RHE, a sudden increase in current density occurs as the OER is activated. From an aerial perspective, nucleation of oxygen bubbles occurs mostly under the orange-sculpted oil layer, where the bubbles continue to form and grow until buoyancy and interfacial forces become greater than the adhesive forces between the oil film and the electrode surface, causing the bubbles to detach. After that, a substantial portion of the oil film is removed, indicating that the hydrophobic by-product has been moved away and partly cleaned from the electrode interface. Additionally, the extra side-view visualization provides additional mechanistic insight into the behaviors within bubble-assisted cleaning processes. Figure 3c, Supplementary Video 1 illustrates that, in the late growth stage of an oxygen bubble, a significant necking phenomenon transpires at the bubble-oil-electrode interface, induced by the robust interfacial interaction between the expanding gas phase and the viscous oil film. Immediately prior to detachment, the bubble is drawing a distinct oily filament or ‘tail’, signifying the mechanical lifting effect exerted by the ascending bubble on the insoluble product layer. Upon the immediate detachment of the bubble, the oily residue diminishes substantially, confirming the efficacy of the bubble-assisted removal. A comparable interfacial cleaning phenomenon was noted on bare nickel mesh electrodes (Supplementary Fig. 17), indicating that the bubble-driven detachment mechanism is substrate-independent and primarily reliant on the physicochemical interactions between gas bubbles and oily product (Supplementary Information Note 2).

Due to the strong interaction between gas and oil phases, bubbles preferentially nucleate and grow within the oil-covered regions. After rupture of the oil film, bubble coalescence proceeds through the formation and evolution of an oil bridge (Fig. 3d, Supplementary Video 2). The curvature of the oil bridge generates a Laplace pressure gradient, which governs the coalescence behavior. In the presence of an oil film, the formation of oil-mediated bridges between adjacent bubbles reduces the interfacial energy barrier and enhances the curvature gradient, thereby accelerating the coalescence process. Consequently, the oil film not only enhances bubble coalescence through dynamic Laplace pressure effects but also promotes efficient removal of the oil layer from the electrode surface, minimizing the probability of secondary fouling.

### Prototype electrolysis couple with BOR and HER

**Fig. 4:**
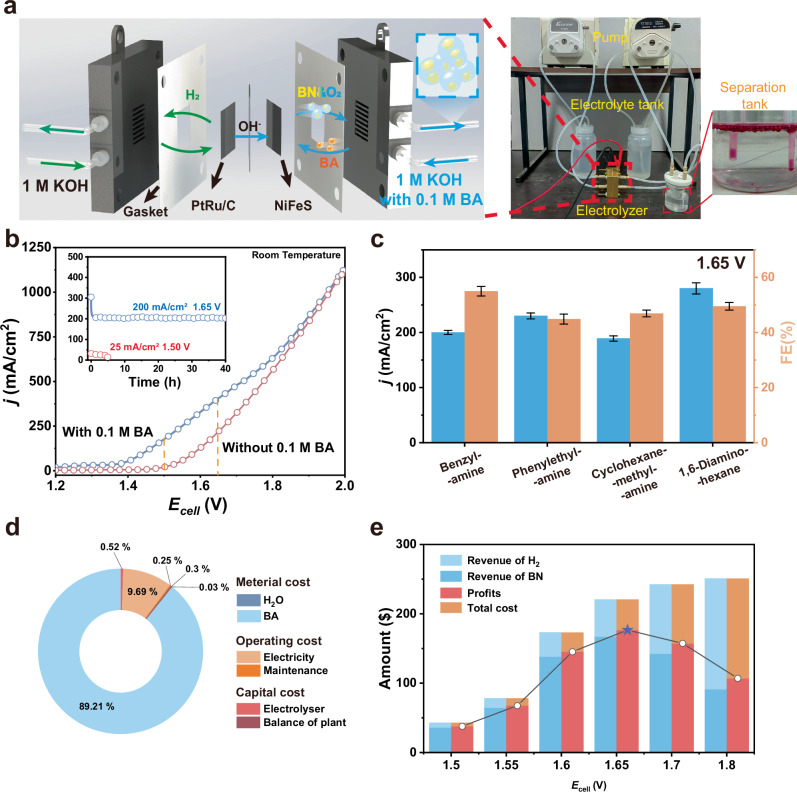
Application for an AEMWE device and techno-economic analysis. **a** Schematic and operating schematic diagram of the AEMWE. **b** LSV curves with and without BA precursor based on a 2 × 2 cm^2^ device. Inset: stability tests (without *iR* correction). **c** Substrate scope of the electrocatalysis of primary amines into nitriles over the catalyst. Error bars represent standard deviation (*n* = 3) (without *iR* correction). **d** Total cost of the BN electrochemical process at conditions of 55%, current density of 200 mA/cm^2^, and cell voltage of 1.65 V. **e** The revenues and profits in the different operating conditions. Source data for (**b–e**) are provided as a Source Data file.

In light of the effectiveness of the bubble-assisted cleaning strategy, we constructed an anion exchange membrane water electrolysis (AEMWE) device (2 × 2 cm^2^) with BOR coupling with Hydrogen Evolution Reaction (HER), using NiFeS as the anode catalyst and commercial PtRu/C as the cathode catalyst. The dual-recirculation operational mode is shown in Fig. 4a. The cathode compartment was typically operated with 1 M KOH solution, and the anode compartment was specifically supplied with 1 M KOH solution containing 0.1 M BA. The solution in the anode compartment was perpetually recirculated through the separation mechanism. The post-reaction solution comprising O_2_ and BN underwent purification through a separation system, wherein bubbles aided in the product’s separation from the solution. The purified reaction solution was reintroduced into the anode compartment for reuse, which maintaining a steady-state operation for AEMWE. As shown in Fig. 4b, the onset voltage is 1.55 V for OER. Following the introduction of the BA solution into the organic chamber, the flow reactor promptly delivers an anodic current from approximately 1.40 V, and ultimately achieves an ampere-level current density at 1.57 V, almost twice of the current density without BA introduction, indicating an obvious promotion for the hydrogen production (Supplementary Fig. 18). To evaluate the influence of bubble-assisted strategy in AEMWE device, the stability under two typical potentials (1.50 V and 1.65 V) which represented were assessed (Fig. 4b inset). The current density at the potential of 1.50 V (without OER) stabilizes eventually at 25 mA/cm^2^. In contrast, after the initial decrease due to the balance between the oily product generation and bubble-assisted removal, the current density at the potential of 1.65 V could be maintained at 200 mA/cm^2^ for over 40 h, demonstrating a promising stability and performance as well. After the operation, the Ni 2*p* and Fe 2*p* signals remain clearly observed at 855.3 eV and 711.0 eV by XPS data, and the Ni_3_S_2_ species could be identified by XRD data, indicating that the surface species are still retained after prolonged operation. (Supplementary Figs. 19, 20). We also extended this strategy for the oxidation of other primary amines to nitriles, as shown in Fig. 4c, indicating the versatility for matching high current density scenarios.

Based on the performance of the BOR-coupled AEMWE device, the TEA of the BOR process was further conducted, incorporating operating costs, capital costs, and material costs (Fig. 4d) (see more details in Supplementary Information Note 3). The TEA results show that the total cost and revenue are $408.63 and $585.27, respectively, indicating a profit of $176.64 with a fixed working area of 10 m^2^ (current density of 200 mA/cm^2^, cell voltage of 1.65 V, FE of 55%). In addition, the in-situ generated oxygen bubbles via OER serve as intrinsic bubble-assisted cleaning and oily product flotation carriers. More importantly, with the efficient bubble-assisted removal, such a unique water-insoluble product inversely saves the extra purification treatment and related costs, which is usually over 50% for conventional overall aqueous paired electrolysis systems. Material costs account for the largest share of total expenses, with the input of BA being the primary cost driver. The comparison of different current densities and FEs on the profitability of BN electrosynthesis was further investigated (Fig. 4e). We can find that the profitability and revenue are highly sensitive to the FE of BN. At low potentials, OER is not activated, and the economic viability remains questionable due to the limited current density induced by the accumulation of oily product on the electrode. As the potential increases, the overall revenue rises continuously and reaches a maximum at 1.65 V, since the extra profits are primarily derived from promoted BN production. While the over-high potentials would lead to a decline of the absolute yield of BN and thereby the net profit, owing to the predominant adsorption of water molecules for OER rather than BA for BOR. The findings suggest that our proposed paired electrolysis exhibits great potential in flexibly coupling with hydrogen production, providing a framework for a modular and add-on retrofittable value-added platform as driven by renewable energy.

In summary, to address the stability and performance limitations caused by the significant adhesion of insoluble products in many anodic organic electro-oxidation processes coupled with ampere-level hydrogen generation, we have devised a bubble-assisted cleaning approach. By tuning the oxidation potential to activate OER, the generated bubbles tend to nucleate from the oily product regime and finally efficiently remove the adhered oily product during the detachment, owing to the strong interaction between gas and oil. With such a mode, we conducted an AEMWE device with NiFeS as the anode catalyst for the electrooxidation of benzylamine and achieved a current density of 200 mA/cm^2^ at 1.65 V with 55% FE, maintaining stable performance for 40 h. TEA suggested that applying appropriate potential to activate OER is beneficial for achieving higher current density and an efficient BOR process, and more importantly, matching the hydrogen production for practical electrolysis systems. This work provides a new paradigm for hybrid electrolysis, maximizing reaction selectivity, current density, and feasibility so that the bubble-mediated strategy assists both stable hydrogen production and value-added chemical synthesis as well as realistic scalability.

## Methods

### Chemicals and materials

Benzylamine (99%), benzonitrile (99.5%), phenylethylamine (98%), cyclohexylamine (99%), cyclohexane carbonitrile (99.5%), hexamethylenediamine (99%) and adiponitrile (99.5%) were purchased from Aladdin Biological Co., Ltd. KOH (95%), Na_2_S (99.99%), and FeCl_3_·6H_2_O (99%) were sourced from Inno-chem Co., Ltd (Beijing, China). Nickel foam and nickel felt with 99.99% purity were purchased from ShengQiang Co., Ltd. The commercial platinum-carbon (PtRu/C, 40%Pt, 20% Ru) catalyst was purchased from Johnson Matthey (Germany). Anion exchange membrane was purchased from Versogen Co., Ltd. All aqueous solutions were prepared using deionized (DI) water from a water purification system. All chemicals were used as received.

### Electrolyte preparation

A 1 M KOH solution (pH=14 ± 0.02) was firstly prepared by dissolving solid KOH into DI water, and treated by ultrasonication with 15 min for dispersion. After that, 0.1 M solutions of different organic reactants were prepared by introducing the corresponding amount, with magnetic stirring for 8 h. The prepared electrolyte was stored in a dark place.

### Synthesis of NiFeS anode

The substrates (nickel felt and foam nickel) were successively treated by ultrasonication in ethanol, 3 M HCl and DI water for 15 min, respectively. The pretreated nickel foam and nickel felt (2 cm × 1 cm × 0.1 cm) were placed in a 25 mL Teflon-lined stainless autoclave containing 10 mL of DI water, 0.8 g Na_2_S, and 50 mg FeCl_3_·6H_2_O, and kept at 120 °C for 12 h. After naturally cooling down, the catalysts were washed with ethanol and DI water, then dried with vacuum drying for 12 h.

### Fabrication of Pt/C cathode

The mixed solvent consists of isopropanol and DI water with a ratio of 3:1. By introducing the Pt/C catalysts (with a Pt loading of 0.2 mg/cm^2^) and 10%-mass fraction anionic polymer, the formed slurry was ultrasonically dispersed for 2 h. After that, the cathode was prepared by spraying the slurry onto the nickel foam or nickel felt substrates.

### Characterizations

The powder XRD patterns were recorded on a RigakuD/Max2550X-ray diffractometer with Cu Ka radiation. The SEM images were obtained with a JEOL JSM 6700 F electron microscope. XPS was performed on an ESCALAB 250 X-ray photoelectron spectrometer with a monochromatic X-ray source.

### Electrochemical measurements

All electrochemical measurements were performed with a three-electrode system with a CHI instrument (Model 650E). The working surface area of the electrode was fixed as 1 cm^2^. A Hg/HgO electrode and a carbon rod were used as reference electrode and counter electrode, respectively, and the Hg/HgO electrode was calibrated prior to all tests. The potentials measured relative to the Hg/HgO electrode were converted to the RHE scale according to the following formula, ${E}_{{vs}.{RHE}}={E}_{{vs}.{Hg}/{HgO}}+0.098{V}+0.059{pH}$. For three-electrode testing, LSV measurements were performed within a voltage range of 0.25–1 V at a scan rate of 5 mV/s, and all acquired LSV curves were processed with 100% *iR* correction.

In contrast, device tests were conducted with a voltage scanning range of 1.1–2 V at the same scan rate of 5 mV/s, and the corresponding LSV results were presented without *iR* correction. All electrochemical experiments, including three-electrode characterization, electrolyzer performance testing, and bubble behavior observation, were carried out at a constant room temperature of 25 °C.

### Reference electrode calibration

Calibration of the target reference electrode was performed with an RHE. To fabricate the RHE, a capillary-attached Pt/C electrode was triple-rinsed with standard KOH solution to eliminate entrapped bubbles. The RHE (cathode) and counter electrode (anode) were assembled in a KOH-containing electrolytic cell, followed by 20 min electrolysis at 10 V to generate hydrogen inside the RHE cavity. The RHE was conditioned by overnight immersion in electrolyte post-electrolysis. The potential of the Hg/HgO electrode versus RHE in standard KOH was quantified by measuring the open-circuit potential difference, with RHE as the reference and Hg/HgO as the working electrode.

### EIS measurements

Electrochemical Impedance Spectroscopy (EIS) measurements were carried out on the assembled three-electrode configuration over a frequency range spanning 100 kHz to 0.1 Hz. The real impedance *R*’ at an imaginary impedance *R*” was defined as the solution resistance, which was determined to be 0.2 ± 0.05 Ω.

### ECSA measurements

After full electrode activation, the potential range was adjusted to the non-Faradaic region (100 mV window) to ensure no electrochemical reactions occurred. Cyclic Voltammetry (CV) measurements were performed at a scan rate of 50 mV/s until the CV curves became completely overlapped. Subsequently, CV tests were conducted at various scan rates of 20, 40, 60, 80, and 100 mV/s. The current density at the mid-potential was recorded for each scan rate. The C_dl_ was determined from the slope of the linear plot of current density versus scan rate. The ECSA was then calculated using a standard specific capacitance value of 40 μF/cm^2^.

### Device setup

The AEM device was assembled with NiFeS as anode catalyst and PtRu/C as cathode catalyst. The electrode area was fixed as 2 × 2 cm^2^. The cathode and anode compartments were separately filled with 1 L KOH solution in the cathode compartment and 5 L KOH solution with BA in the anode compartment. Piper-ION A40 of 3.5 × 3.5 cm^2^ were used as the anion exchange membrane.

### Evaluation of faradaic efficiency and yield

The liquid products were analyzed quantitatively by GC-MS (Shimadzu GC-2014C) equipped with an FID detector. An internal standard method was employed, using n-tridecane as the internal standard. A series of five concentration gradients of BA and BN solutions was prepared, and standard calibration curves were generated. The Mass Hunter software was utilized to compare the standard and test samples, enabling the determination of the product BA yield as,

2 $$FE\, \% =\frac{{mole}\,{of}\,{product}\,{perform}}{\frac{{total}\,{chargepassed}}{n \times F}}$$

where *F* is Faradic constant.

## Supplementary information

Supplementary Information

Description of Additional Supplementary Files

Supplementary Video 1

Supplementary Video 2

Transparent Peer Review file

## Source data

Source data

## Data Availability

The data generated in this study are provided in the Source Data file. Source data are provided with this paper.
