# Supplementary material for: Bubble-assisted cleaning for primary amine oxidation towards ampere-level current densities
Source: Nat Commun. 2026 Jun 26;17:8015. doi: 10.1038/s41467-026-74878-0 (PMC13454444; doi:10.1038/s41467-026-74878-0)
Supplement: Supplementary file 1 [file 41467_2026_74878_MOESM1_ESM.pdf]

Supplementary Information

Bubble-assisted cleaning for primary amine oxidation towards ampere-level current densities

Xichang Lin[1, #], Bo Li[1, #], Yisong Li[2,3,4], Anuj Kumar[5], Bin Ding[2,3,4], Jiazhong Wu[2,3,4], Liang Luo[1] and Xiaoming Sun[1]*

[1] State Key Laboratory of Chemical Resource Engineering, Beijing University of Chemical Technology, Beijing, China.

[2] Research Institute of Petroleum Exploration & Development, China National Petroleum Corporation

[3] State Key Laboratory of Enhanced Oil & Gas Recovery

[4] Key Laboratory of Oilfield Chemistry (KLOC), CNPC

[5] Nano-Technology Research Laboratory, Department of Chemistry, GLA University, Mathura, Uttar Pradesh 281406, India

* Corresponding Author

* E-mail: luoliang@mail.buct.edu.cn

[#] These authors contributed equally to this work.

This PDF file includes :

Supplementary Notes S1 to S3

Supplementary Figs. S1 to S21

Supplementary Tables S1 to S2

Supplementary Notes

Note 1. Principle of underwater oil droplet contact angle

According to the Young's equation,

$$\gamma_{lg}\cos\theta_{lg} = r(\gamma_{sg} - \gamma_{\mathrm{sl}}) \quad (1)$$

where γ_{lg}, γ_{sg} and γ_{sl} are the liquid-gas interface tension, solid-gas interface tension and solid-liquid interface tension. Liquid represents water or oil. Based on the equation, we can obtain the interface tension of water and oil in air, respectively.

$$\gamma_{wg} = \frac{\gamma_{sg}-\gamma_{\mathrm{sw}}}{\cos\theta_{wg}} \quad (2)$$

$$\gamma_{og} = \frac{\gamma_{sg}-\gamma_{\mathrm{so}}}{\cos\theta_{og}} \quad (3)$$

Thus the contact angle of oil underwater can be expressed as follow[1].

$$\cos\theta_{og} = \frac{\gamma_{og}\cos\theta_{og}-\gamma_{wg}\cos\theta_{wg}}{\gamma_{ow}} \quad (4)$$

As the value of surface tension of water in air $\gamma_{wg} = 72\,mN/m$, we can obtain $\gamma_{og} = 38.8\,mN/m$. With θ_{og} and θ_{wg} as variables, we can get contour charts, as shown in Supplementary Fig. 5. The electrode is underwater oleophobic as the contact angle is less than 57.8°. The summary of different wettabilities at various Interfaces can be conducted in Table S1.

Note 2. Theoretical model

In the process of coalescence, oil droplet and bubble are regarded as spheres. Before the coalescence, their total interface energy (E_1) is as follows.

$$E_1 = 4\pi r_o^2 \gamma_{ow} + 4\pi r_g^2 \gamma_{gw} \quad (5)$$

Where r_o and r_g are the radius of oil droplet and bubble, respectively. γ_{ow} and γ_{gw} are the oil-water interface tension and gas-water interface tension, respectively. After the coalescence, the total interface energy of oil droplet and bubble (E_2) is as follows. Assuming the oil droplet is completely enclosed by a bubble[2].

$$E_2 = 4\pi (r_o^3 + r_g^3)^{2/3} \gamma_{gw} + 4\pi r_o^2 \gamma_{og} \quad (6)$$

Where γ_{og} is the oil-gas interface tensionthe volume of oil droplet is enlarged by bubble and the little change in bubble volume is ignored in this process.

$$(r_o^3 + r_g^3)^{\frac{2}{3}} \approx r_g^2 \quad (7)$$

Then the difference of total interface energy (ΔE) is as follows.

$$\Delta E = 4\pi r_o^2 (\gamma_{og} - \gamma_{ow}) \quad (8)$$

Because,

$$\gamma_{og} - \gamma_{ow} < 0 \quad (9)$$

The coalescence phenomenon would be spontaneous.

Note 3. Techno-economic analysis (TEA)

To evaluate the economic potential for the electrosynthesis of BN, TEA was carried out. Notably, the price of electricity, H_2O, KOH, and H_2 selected in this study is based on the recently published literature.

The anticipated service life of the electrolyzer is 20 years and the working time per year is 350 days. The specific sample for the calculation of BN production under optimistic case assumptions: Take a reactor with a fixed working area of

10 m^2 was considered as an example at a current density of 0.2 A/cm^2 with 55% FE of BN at anode and 99% of H_2 at cathode as an example[3]:

1. The electricity price is considered to be 0.05 $/ (kW h).
2. The price of H_2O is 0.0007 $/kg. The price of KOH is 0.82 $/kg. The price of H_2 was 3 $/kg. The price of BA is 1.38 $/kg. The price of BN is 2.07 $/kg.
3. The operating voltage is set as 1.65 V.
4. The electrolyzer cost per area is calculated as follow,

$$450\ \$/kW \times 1.65\ \mathrm{V} \times 0.2\ A/cm^2 \times 10^4 \times 10^{-3} = 1485\ \$/m^2 \quad (10)$$

5. The required total current is calculated as,

$$0.2\ A/cm^2 \times 10\ m^2 \times 1000\ cm^2/m^2 = 20000\ A \quad (11)$$

The power of BM production is calculated as follow,

$$20000\ A \times 1.65\ V \times 10^{-3} = 33\ kW \quad (12)$$

Capital cost:

Electrolyzer cost:

$$10.0\ m^2 \times 1485\ \$/m^2 \div 20\ year = 2.06\ \$ \quad (13)$$

Balance of Plant:

$$2.06\ \$/ton \times 58\% = 1.20\ \$ \quad (14)$$

Operating cost:

1. Electricity:

$$33\ kW \times 24\ h\ \times 0.05\ \$\ /(kW \cdot h) = 39.6\ \$ \quad (15)$$

2. Maintenance of electrolyzer:

$$10.0\ m^2 \times 1485\ \$/m^2\ \times 0.025 \div 350\ day = 1.03\ \$ \quad (16)$$

Material cost:

1. Water

$$0.0007\ \$/ton \times 18\ \mathrm{kg/mol} \times \frac{20000\ \mathrm{A} \times 99\ \% \times 24\ \mathrm{h} \times 3600\ \mathrm{s/h}}{96485 \times 2} \div 1000\ kg/ton = 0.056\ \$ \quad (17)$$

Water consumption costs are omitted from the analysis as they accounted for

less than 0.1% of the total expenses.

2. BA

$$1.38\ \$/kg\ \times 107.15\ \mathrm{kg/mol} \times \frac{20000\ \mathrm{A} \times 55\ \% \times 24\ \mathrm{h} \times 3600\ \mathrm{s/h}}{96485 \times 4} = 364.74\ \$ \tag{18}$$

3. KOH

Owing to the potential for KOH recovery and reuse, it is not included in the overall cost assessment.

Total cost:

$$2.06 + 1.20\ + 39.6 + 1.03 + 364.74 = 408.63\ \$ \tag{19}$$

Revenue:

1. BN

$$1.38\ \$/kg\ \times 104.21\ \mathrm{kg/mol} \times \frac{20000\ \mathrm{A} \times 55\ \% \times 24\ \mathrm{h} \times 3600\ \mathrm{s/h}}{964854 \times 4} = 532.08\ \$ \tag{20}$$

2. H_2

$$3\ \$/kg\ \times 2\ \mathrm{kg/mol} \times \frac{20000\ \mathrm{A} \times 99\ \% \times 24\ \mathrm{h} \times 3600\ \mathrm{s/h}}{964854 \times 4} = 53.19\ \$ \tag{21}$$

Total Revenue:

$$532.08 + 53.19 = 585.27\ \$ \tag{22}$$

Supplementary Figures

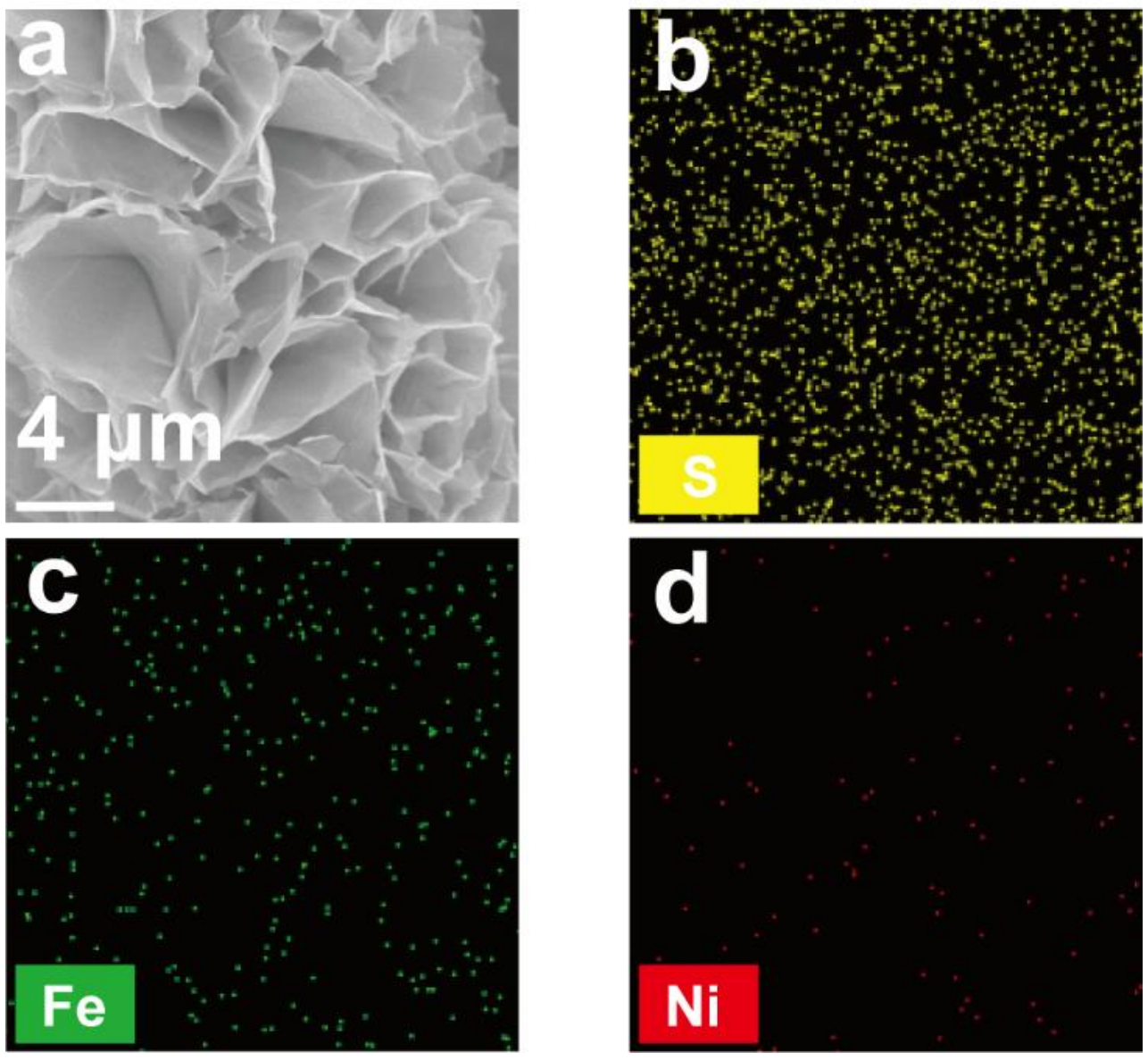

Supplementary Fig. 1. The SEM/EDS characterization of the NiFeS coated on nickel felt. a) SEM image of NiFeS catalyst, EDS of element b) S, c) Fe and d) Ni.

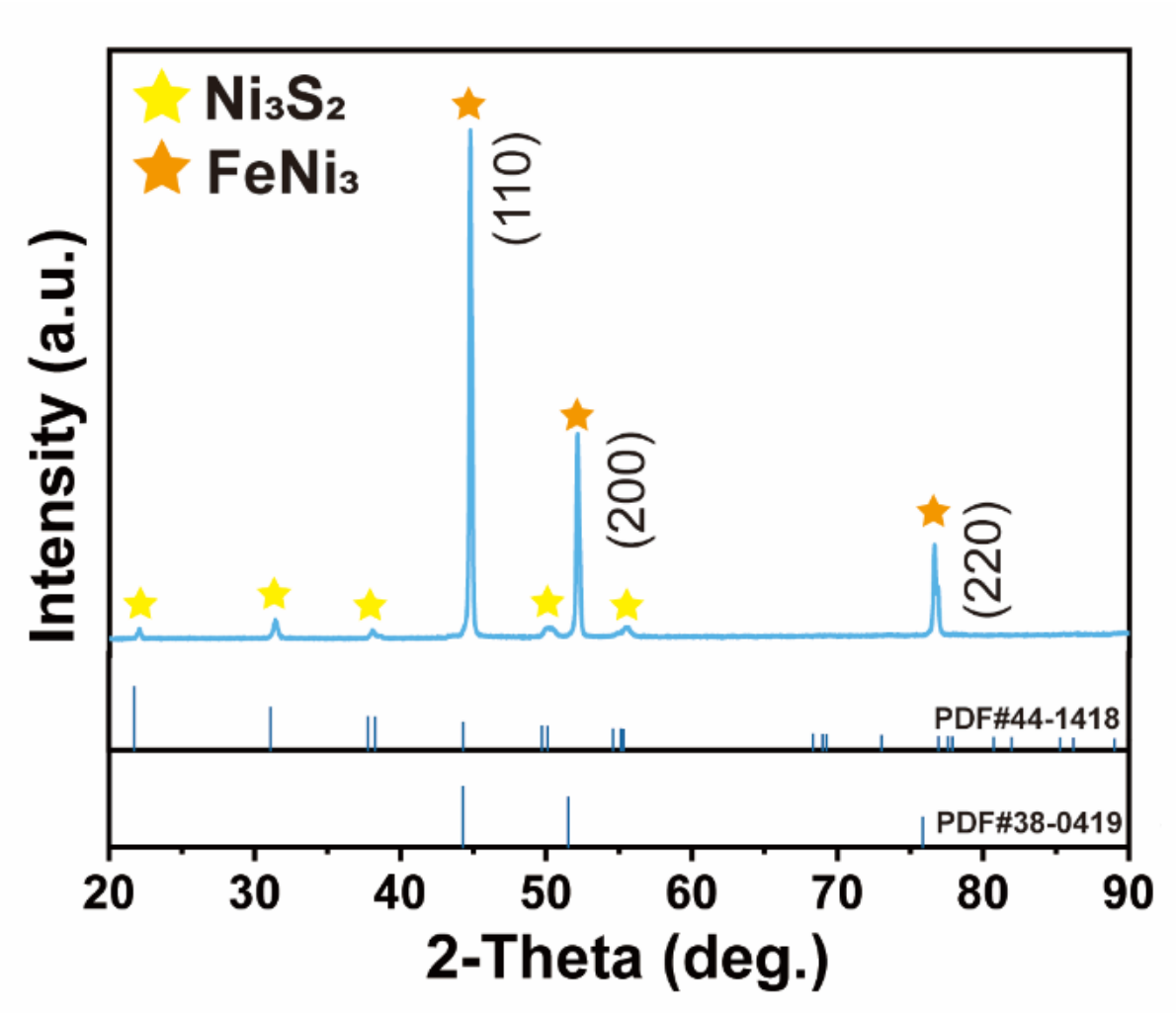

Supplementary Fig. 2. The XRD patterns of the NiFeS catalyst coated on nickel felt.

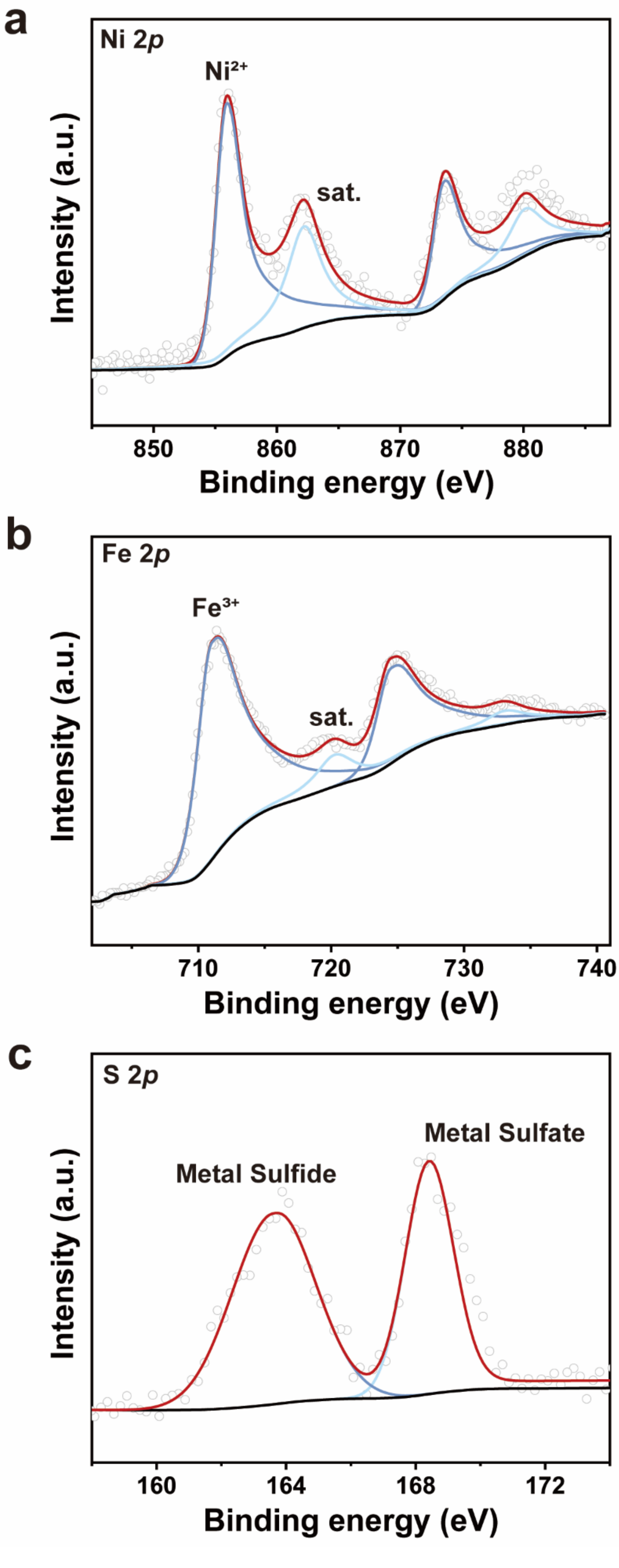

Supplementary Fig. 3. The XPS spectra of a) Ni 2p b) Fe 2p and c) S 2p for NiFeS catalyst.

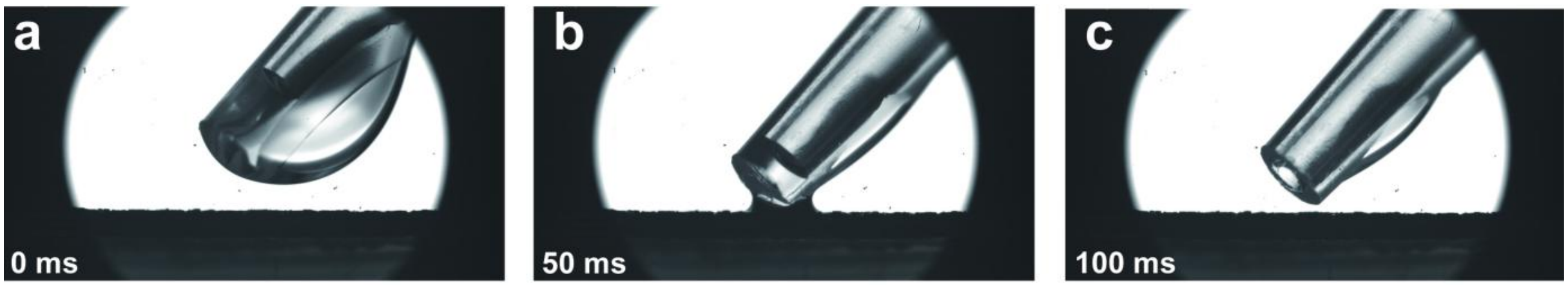

Supplementary Fig. 4. The diffusion process of BN on NiFeS/NF.

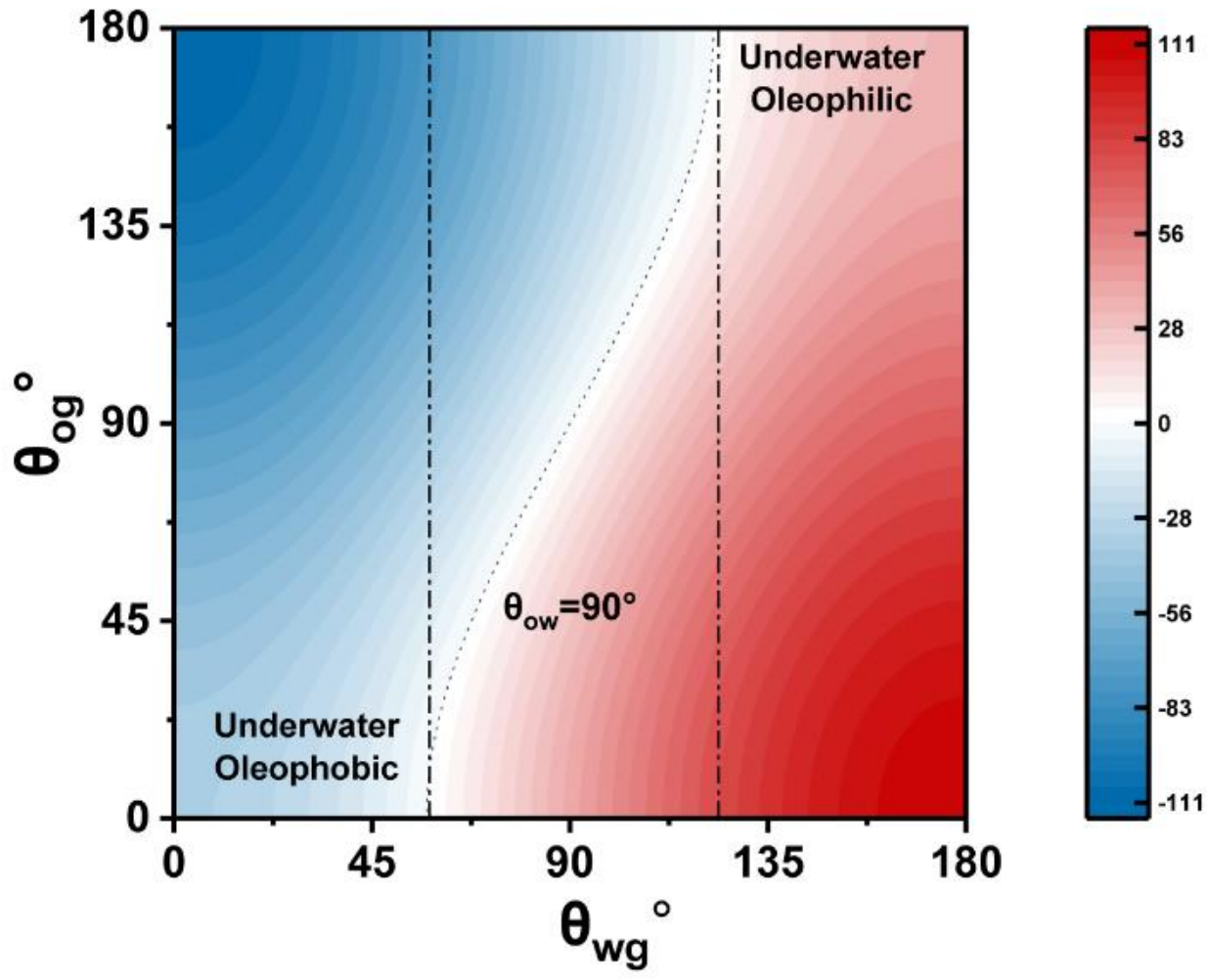

Supplementary Fig. 5. The principle of water-gas surface tension and oil-gas surface tension.

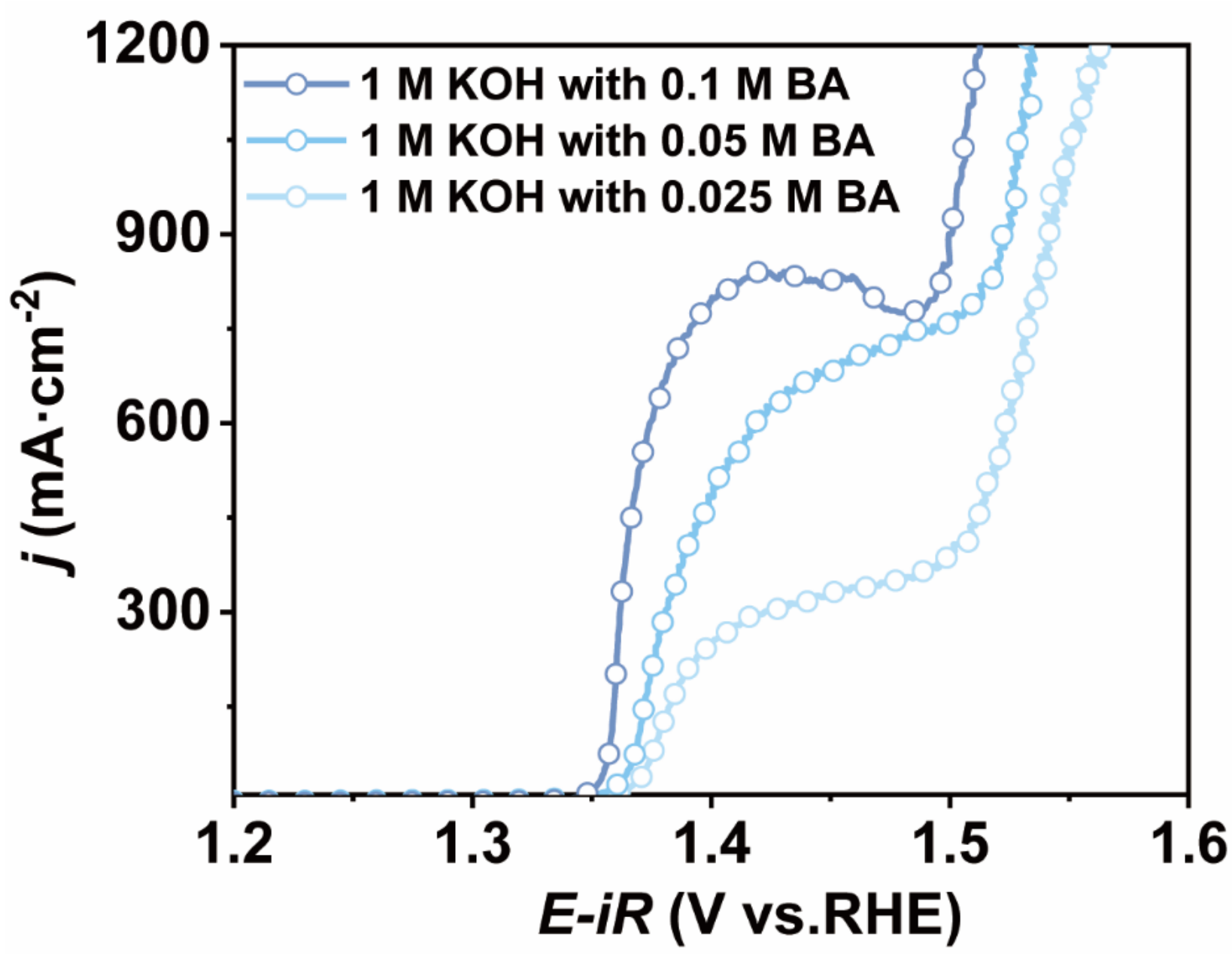

Supplementary Fig. 6. LSV curves of NiFeS catalyst on nickel felt for BOR at different BA concentrations (1 M KOH with 25, 50, 100 mM BA) (with 100% *iR* correction and $R_{s,\ 0.025\ M\ BA}$ =0.25 Ω, $R_{s,\ 0.05\ M\ BA}$ =0.18 Ω, $R_{s,\ 0.1\ M\ BA}$ =0.19 Ω. The non-*iR* corrected voltammograms are provided in Supplementary Fig. 21).

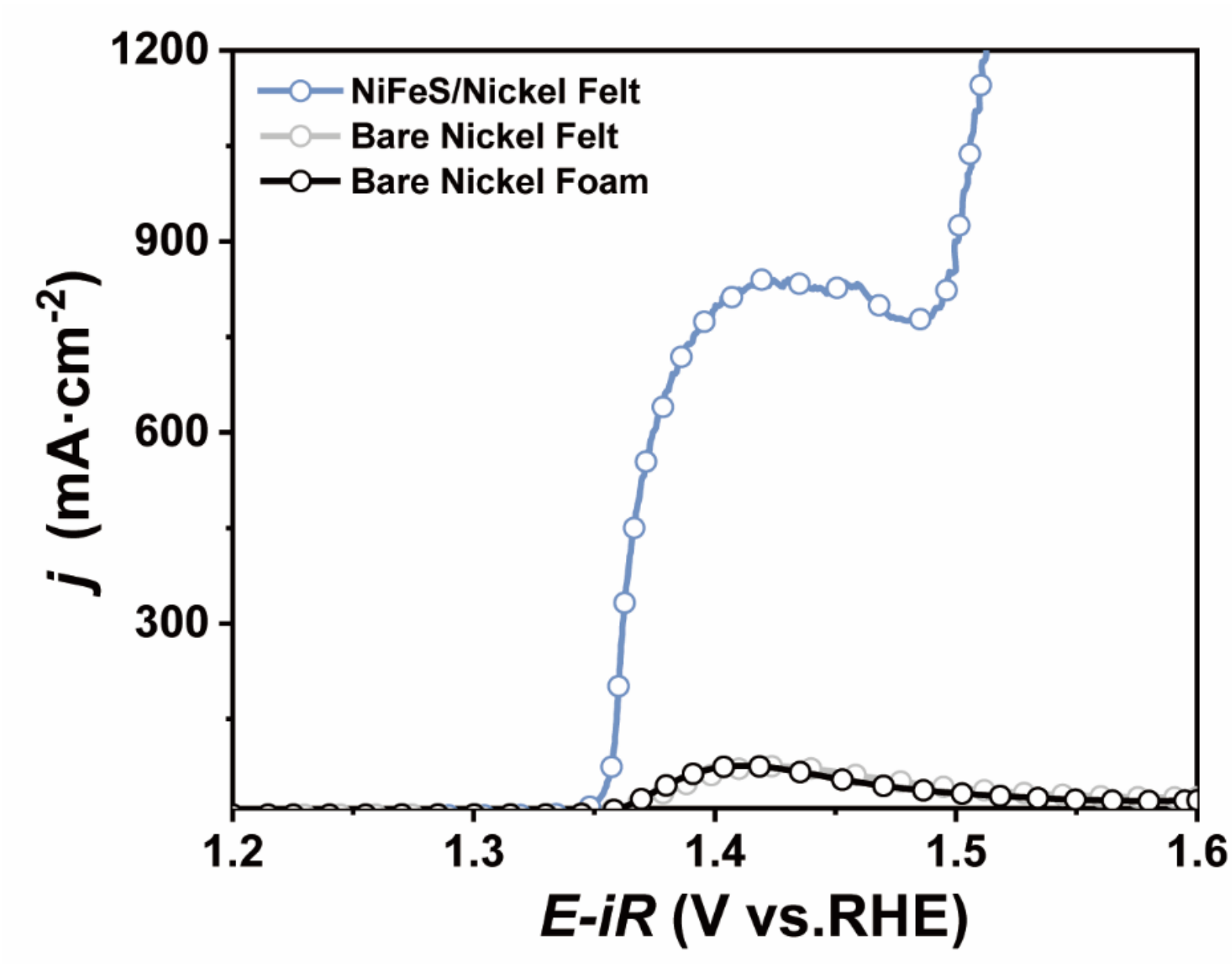

Supplementary Fig. 7. Polarization curves for benzylamine electrooxidation on NiFeS catalyst, bare nickel felt and bare nickel foam (with 100% *iR* correction and $R_{s,\ Bare\ Nickel\ Foam}$ =0.35 Ω, $R_{s,\ Bare\ Nickel\ Felt}$ =0.21 Ω, $R_{s,\ NiFeS/Nickel\ Felt}$ =0.19 Ω. The non-*iR* corrected voltammograms are provided in Supplementary Fig. 21).

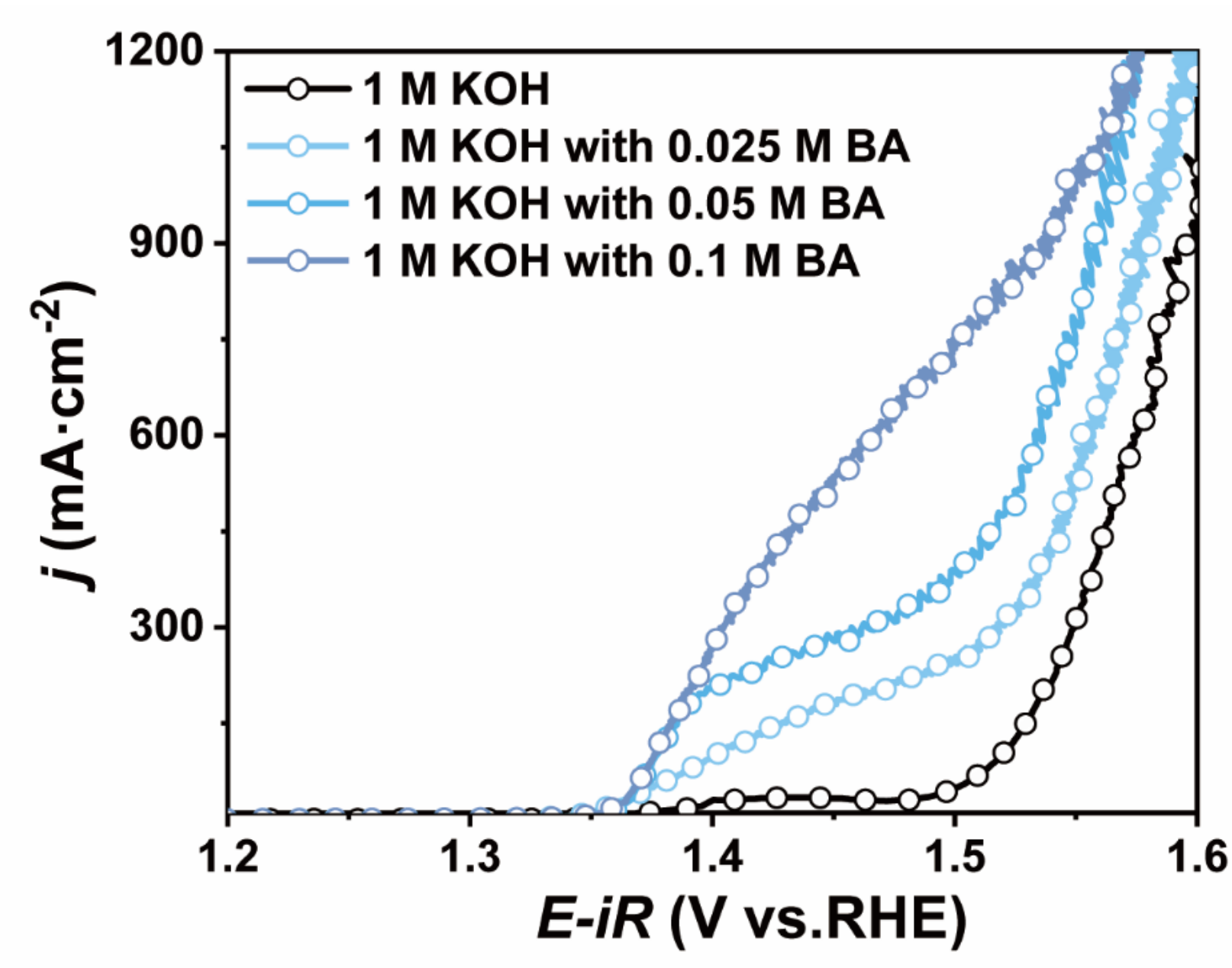

Supplementary Fig. 8. LSV curves of NiFeS catalyst on nickel foam for BOR at different BA concentrations (1 M KOH with 25, 50, 100 mM BA) (with 100% *iR* correction and $R_{s,\ 1\ M\ KOH}$ =0.14 Ω, $R_{s,\ 1\ M\ KOH\ with\ 0.025\ M\ BA}$ =0.21 Ω, $R_{s,\ 1\ M\ KOH\ with\ 0.05\ M\ BA}$ =0.10 Ω, $R_{s,\ 1\ M\ KOH\ with\ 0.1\ M\ BA}$ =0.13 Ω. The non-*iR* corrected voltammograms are provided in Supplementary Fig. 21).

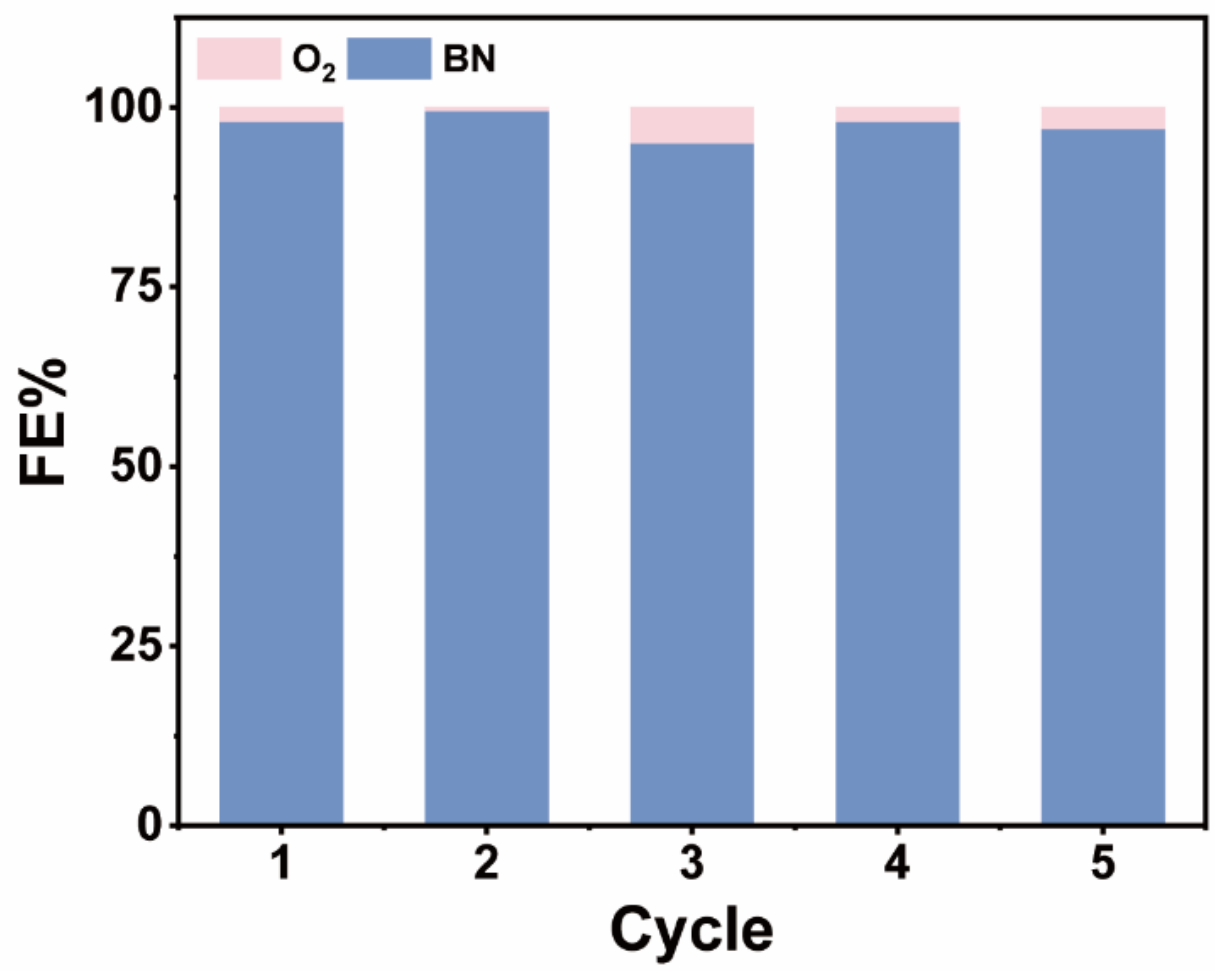

Supplementary Fig. 9. The FE variaiton for 5 cycles of BOR.

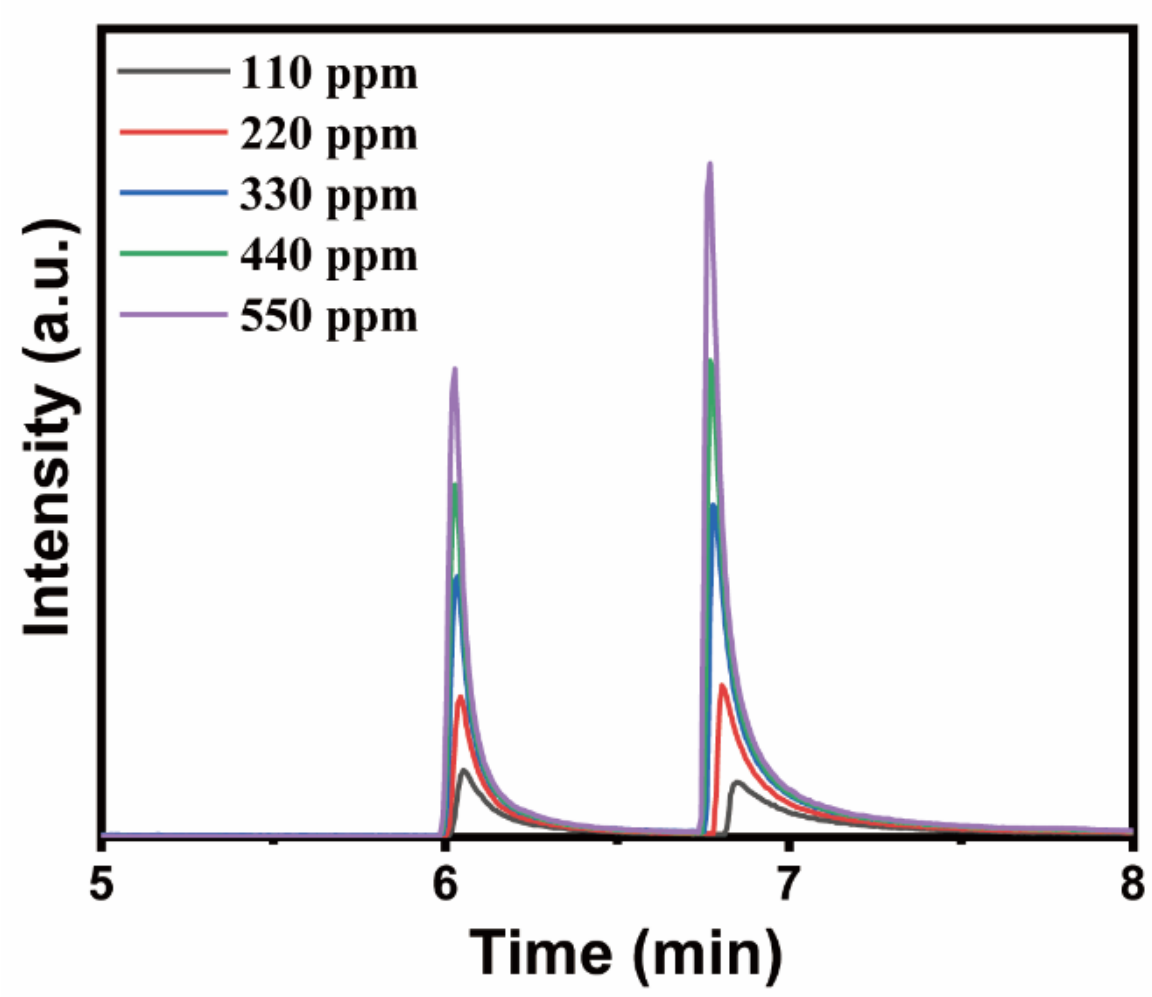

Supplementary Fig. 10. The standard samples of BA and BN tested by gas chromatography-mass spectrometry (GC-MS).

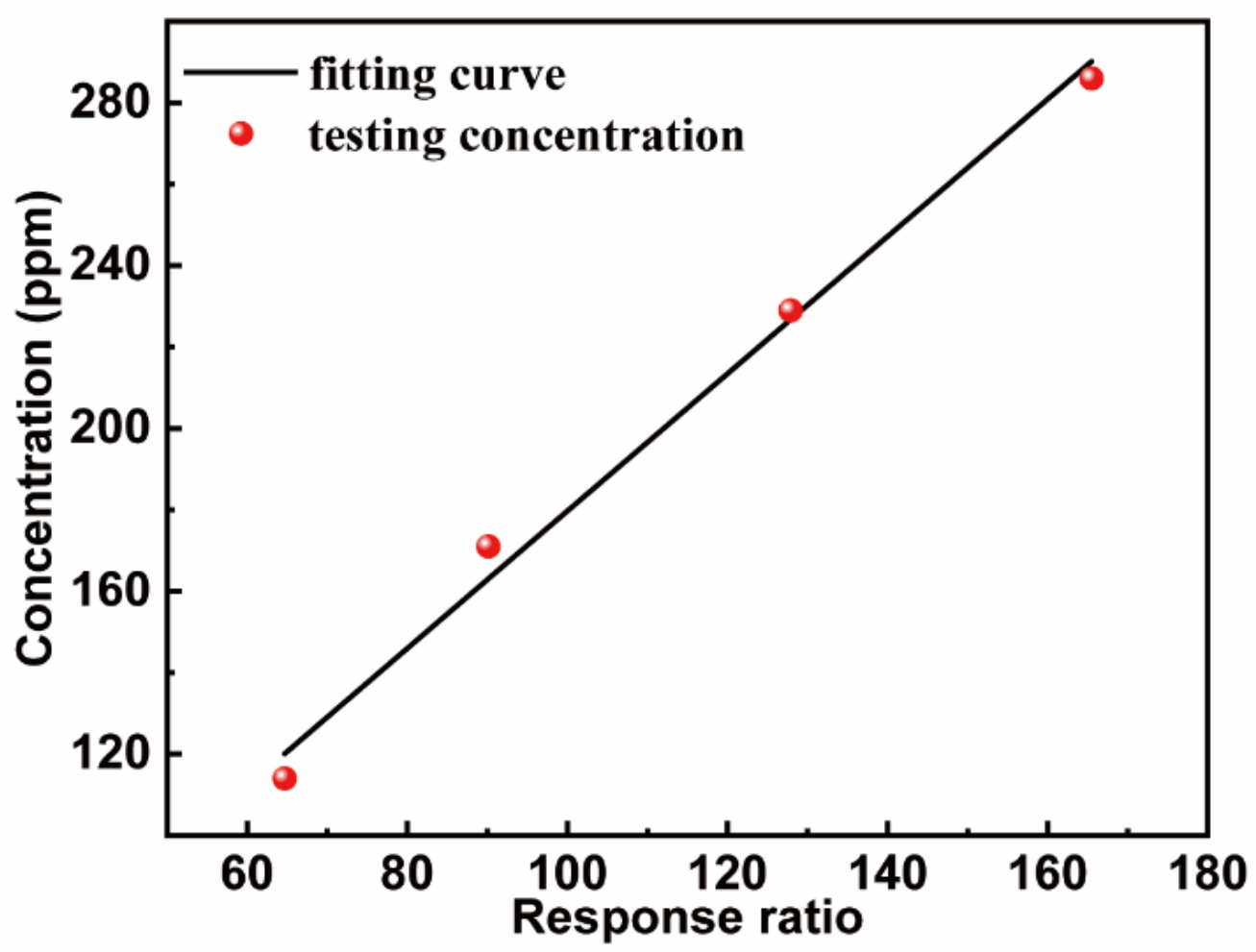

Supplementary Fig. 11. The standard curve of BA and BN tested by GC-MS.

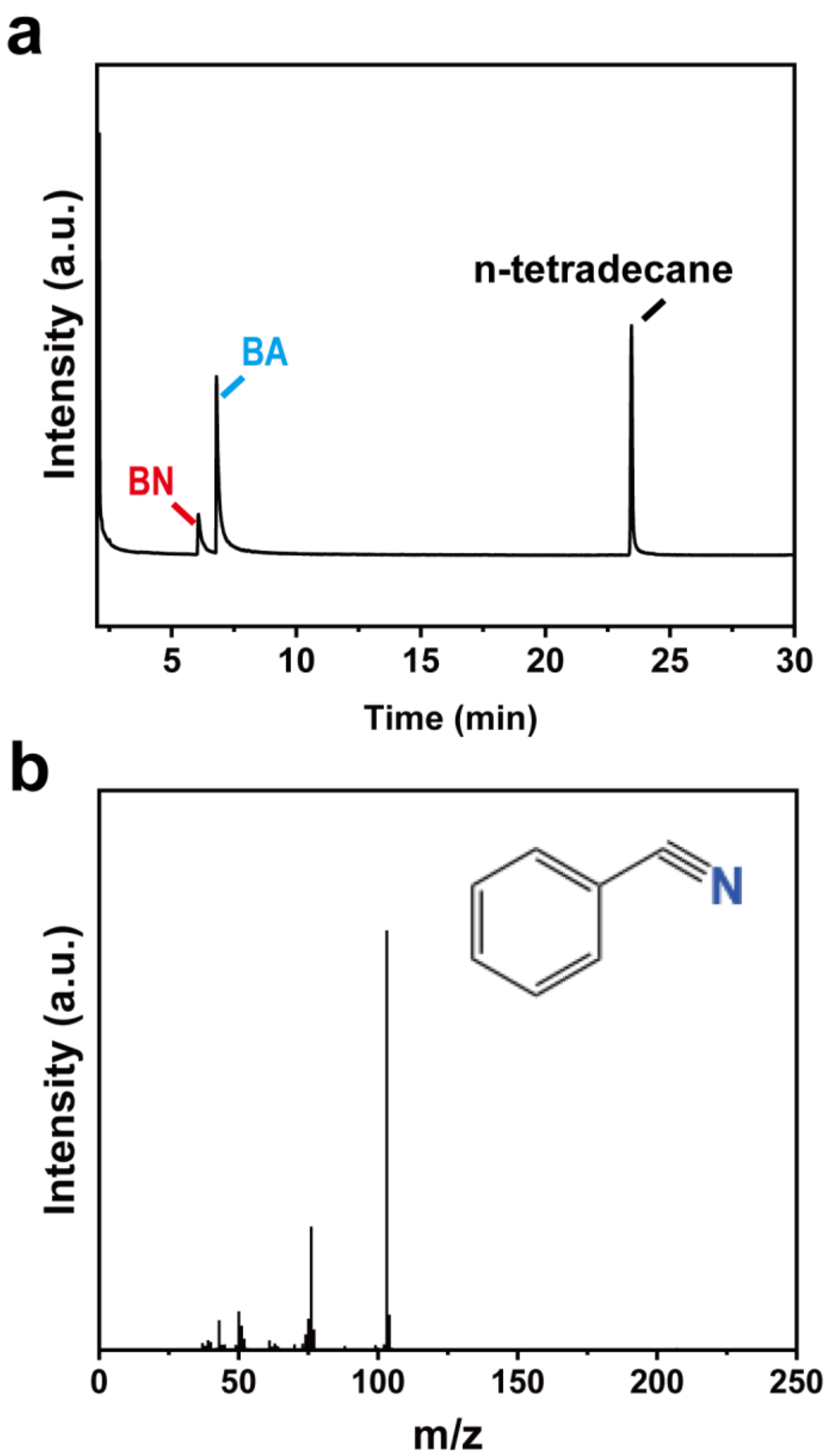

Supplementary Fig. 12. (a) GC chromatogram of the reaction mixture.(b) MS Spectrum at Retention Time 6.071 min

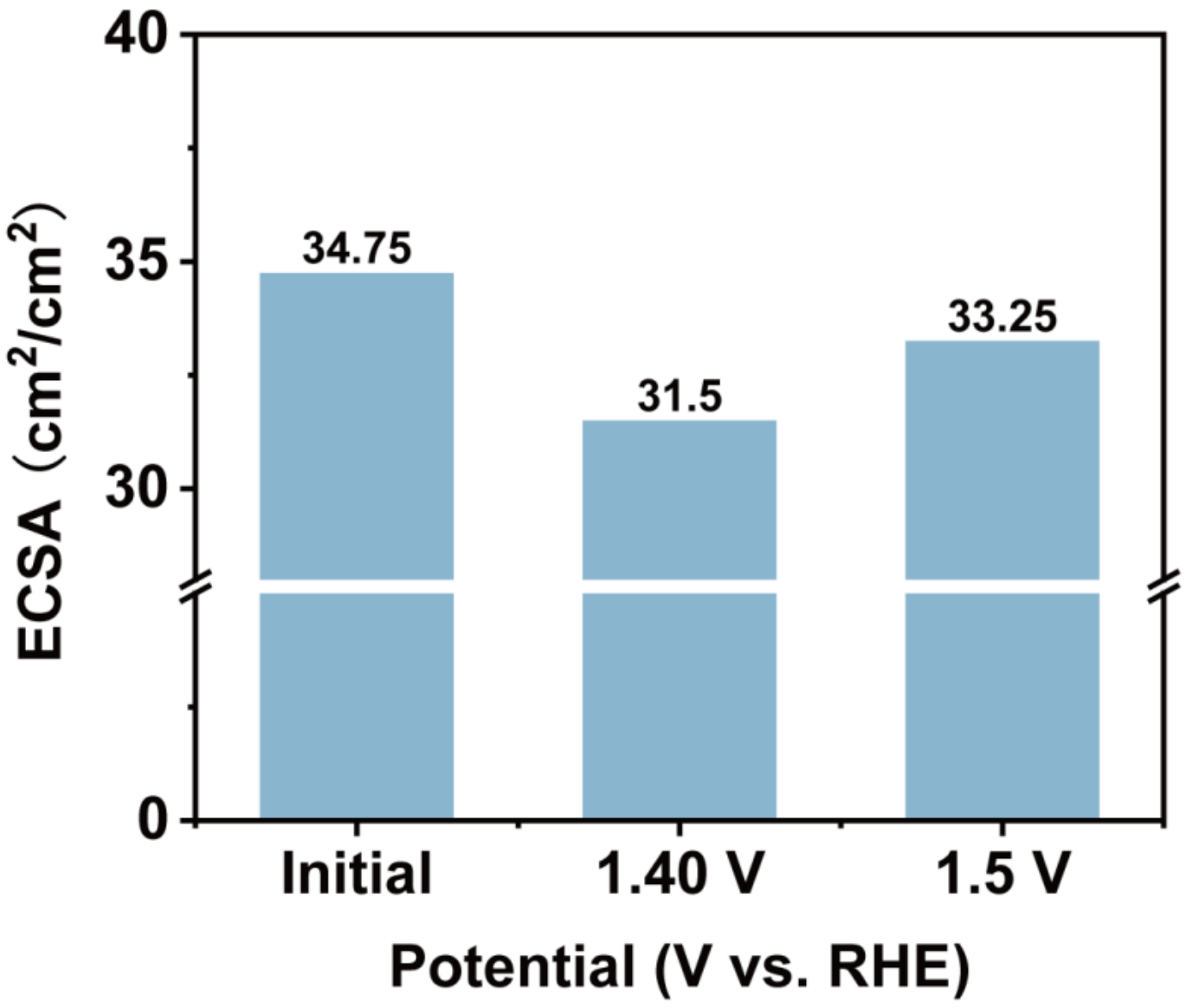

Supplementary Fig. 13. Electrochemical surface area (ECSA) of the electrode at different potentials

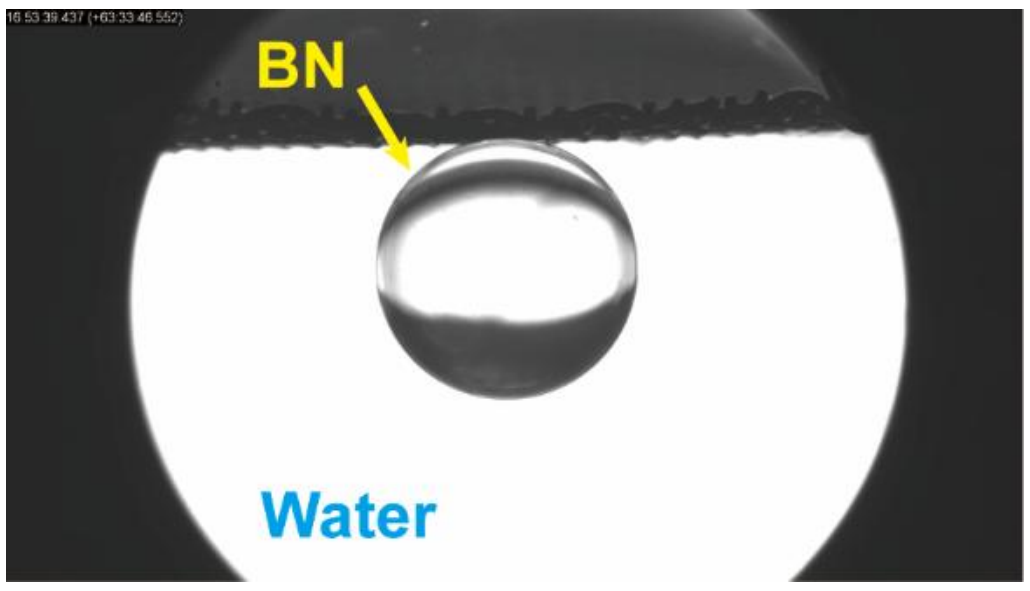

Supplementary Fig. 14. The underwater contact angle of BN droplet on NiFeS coated nickel mesh.

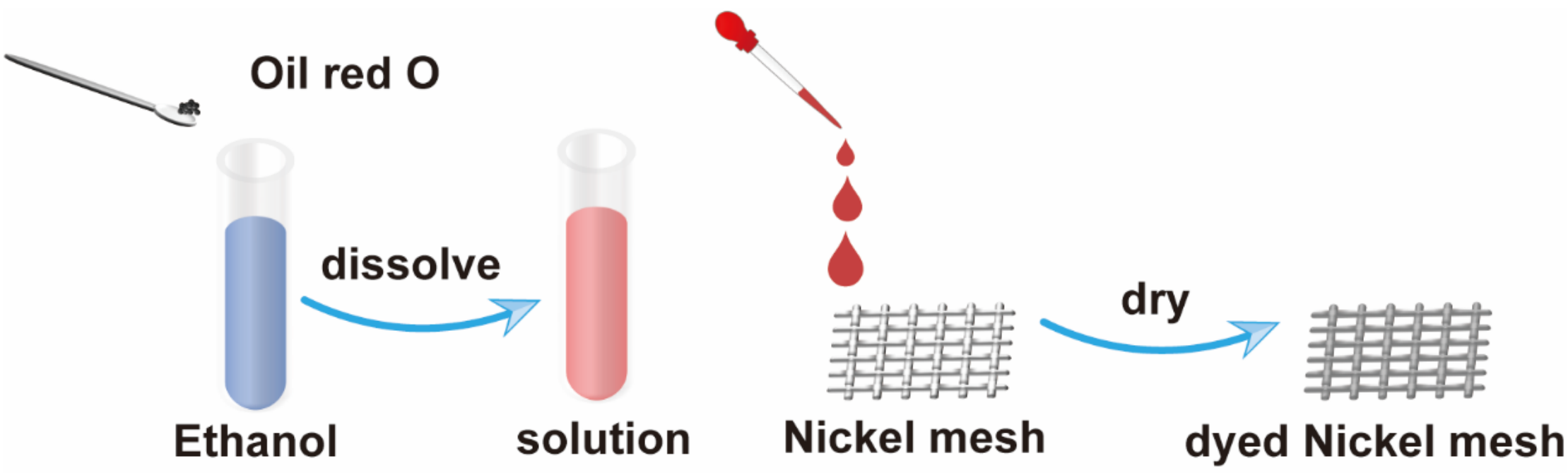

Supplementary Fig. 15. The scheme of dyeing with Oil Red O.

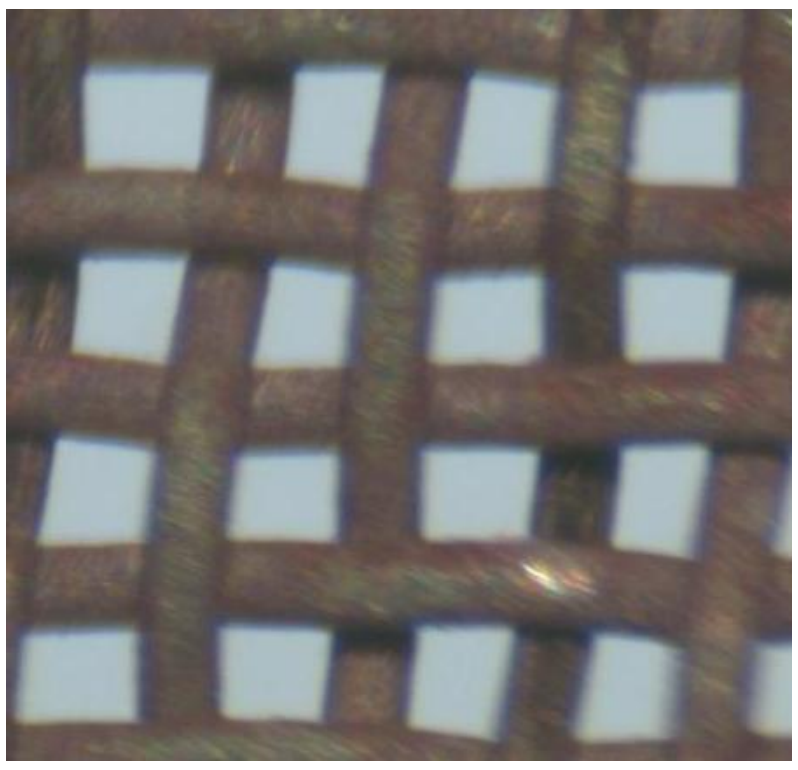

Supplementary Fig. 16. The initial sample after dyeing by oil red O.

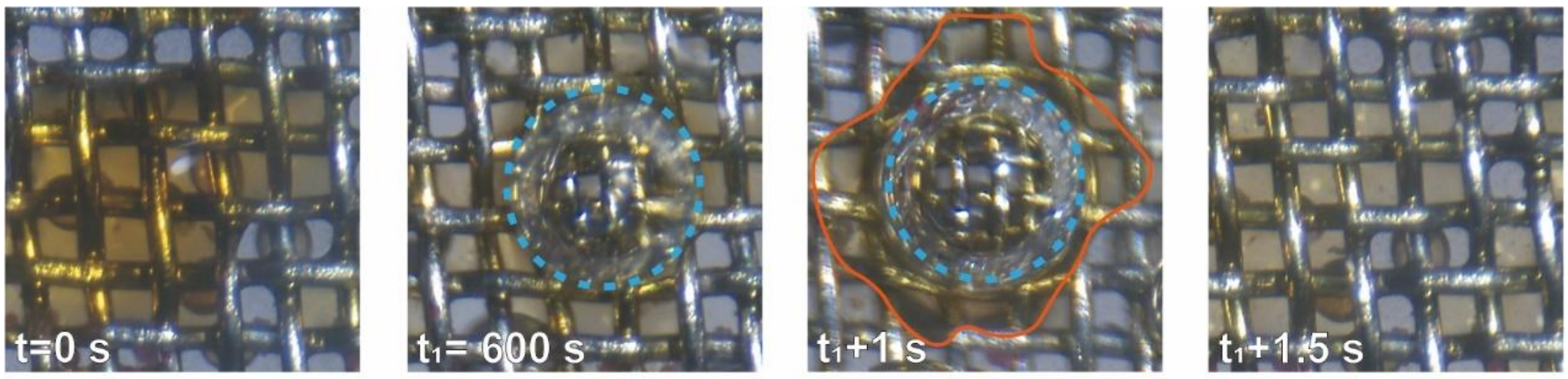

Supplementary Fig. 17. The process of the bubble-assisted oil droplet cleaning on bare nickel mesh.

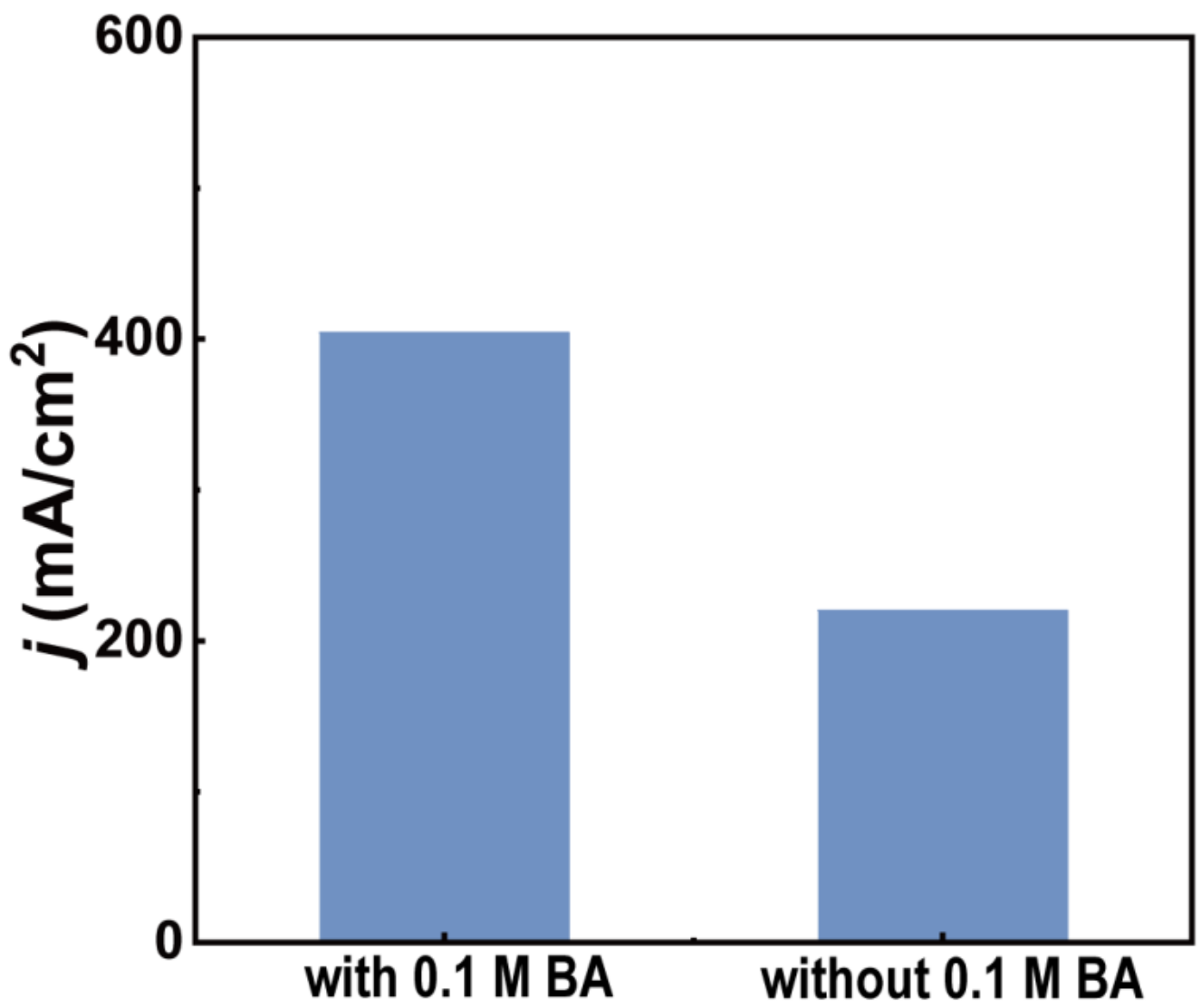

Supplementary Fig. 18. Comparison of current densities in AEMWE with and without benzylamine (BA) at 1.65 V.

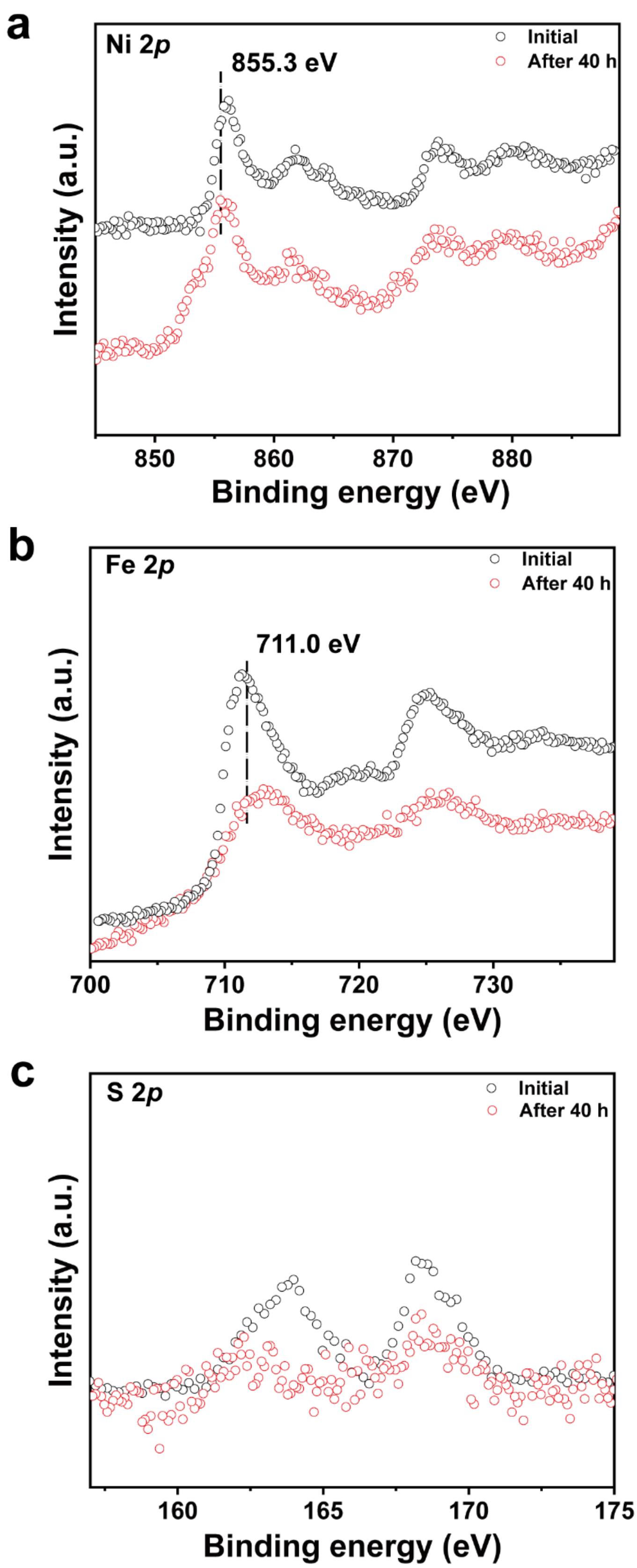

Supplementary Fig. 19. XPS spectra of the NiFeS catalyst before and after 40 h electrolysis: (a) Ni 2p, (b) Fe 2p, and (c) S 2p regions.

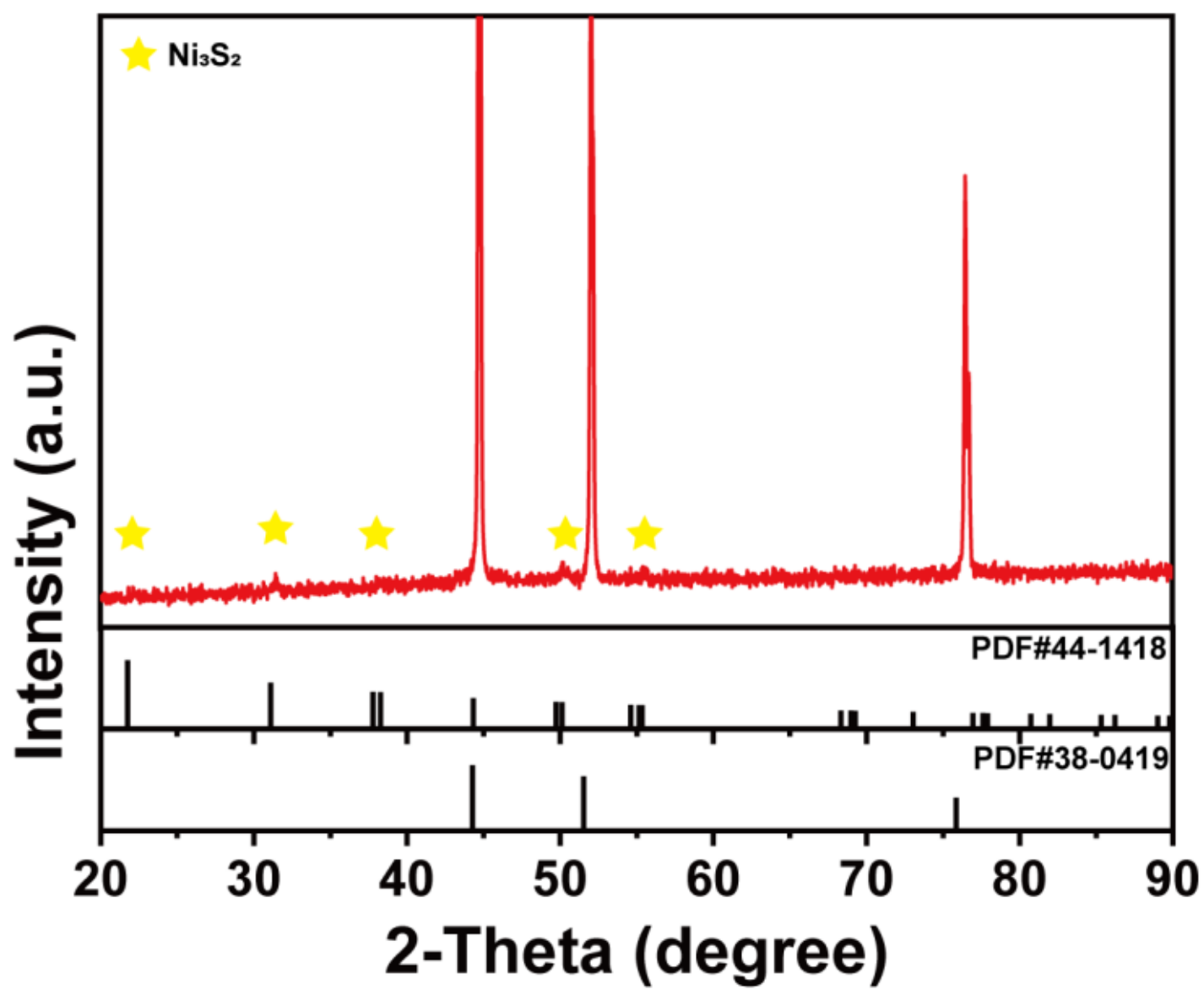

Supplementary Fig. 20. XRD pattern of the NiFeS catalyst after 40 h electrolysis

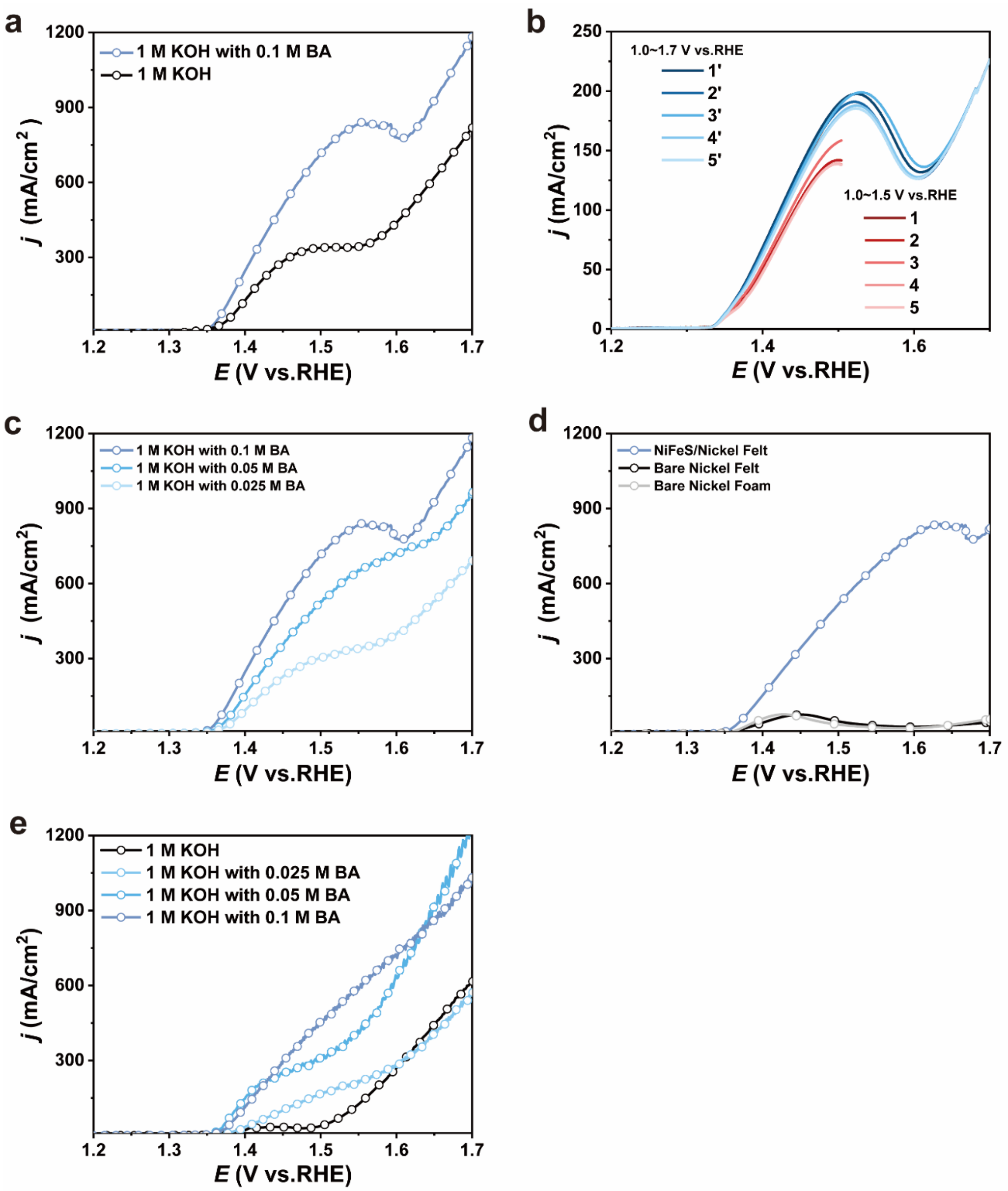

Supplementary Fig. 21. The non-*iR* corrected voltammograms of all electrodes. (a) Polarization curves of NiFeS catalyst in 1 M KOH with and without 0.1 M BA. (b) Multi-step LSV test and current density peaks at different scan ranges with a scan rate of 10 mV/s, (c) LSV curves of NiFeS catalyst on nickel felt for BOR at different BA concentrations (1 M KOH with 25, 50, 100 mM BA), (d) Polarization curves for benzylamine electrooxidation on NiFeS catalyst, bare nickel felt and bare nickel foam ,(e) LSV curves of NiFeS catalyst on nickel foam for BOR at different BA concentrations (1 M KOH with 25, 50, 100 mM BA)

Supplementary Table 1. Summary of oleo/hydrophilicity/phobicity at various interfaces

Solid-air-water interface	Criterion	Solid-water-oil interface
$\theta_{wg} < 57°$	none	Oleophobic
$57° < \theta_{wg} < 127°$	$\gamma_{og}\cos\theta_{og} > \gamma_{wg}\cos\theta_{wg}$	Oleophobic
	$\gamma_{og}\cos\theta_{og} < \gamma_{wg}\cos\theta_{wg}$	Oleophilic
$\theta_{wg} > 127°$	none	Oleophilic

Supplementary Table 2. Price assumptions and data sources used in the techno-economic analysis (TEA)

Item	Price (USD)	Unit	Source
Benzylamine (BA)	1.38	\$/kg	*GuideChem*[a]
Benzonitrile (BN)	2.07	\$/kg	*GuideChem*[a]
Hydrogen (H_2)	3	\$/kg	*GuideChem*[a]
Electricity	0.05	\$/kWh	*Nat. Commun.* **2024**, *15*, 9151.

[a] Price data obtained from the GuideChem database (https://china.guidechem.com), based on market quotations.

Reference

1. Chen C, Weng D, Mahmood A, Chen S, Wang J. Separation Mechanism and Construction of Surfaces with Special Wettability for Oil/Water Separation. *ACS Appl. Mater. Interfaces* 11, 11006-11027 (2019).

2. Li H*, et al.* Bioinspired Dynamic Antifouling of Oil-Water Separation Membrane by Bubble-Mediated Shape Morphing. *Adv. Funct. Mater.* 33, 2212582 (2023).

3. Liu X*, et al.* Electrosynthesis of adipic acid with high faradaic efficiency within a wide potential window. *Nat. Commun.* 15, 7685 (2024).
