# Supplementary material for: Bubble-assisted cleaning for primary amine oxidation towards ampere-level current densities
Source: Nat Commun. 2026 Jun 26;17:8015. doi: 10.1038/s41467-026-74878-0 (PMC13454444; doi:10.1038/s41467-026-74878-0)
Supplement: Supplementary file 2 [file 41467_2026_74878_MOESM2_ESM.pdf]

Description of Additional Supplementary Files

File Name: Supplementary Video 1

Description: Video of the gas-in-oil droplets detachment.

File Name: Supplementary Video 2

Description: Video of the bubble coalescence process between two reaction sites by oil film bridge.
