# Supplementary material for: Bubble-assisted cleaning for primary amine oxidation towards ampere-level current densities
Source: Nat Commun. 2026 Jun 26;17:8015. doi: 10.1038/s41467-026-74878-0 (PMC13454444; doi:10.1038/s41467-026-74878-0)
Supplement: Supplementary file 3 [file 41467_2026_74878_MOESM5_ESM.pdf]

nature portfolio

Peer Review File

Bubble-assisted cleaning for primary amine oxidation towards ampere-level current densities

Corresponding Author: Professor Liang Luo

Parts of this Peer Review File have been redacted as indicated to remove third-party material.

This file contains all reviewer reports in order by version, followed by all author rebuttals in order by version.

Version 0:

Reviewer comments:

Reviewer #1

(Remarks to the Author)
The manuscript reports a bubble-assisted method to lower electrode fouling for benzylamine electrooxidation coupled with hydrogen evolution. While the system demonstrates high current densities in AEM water electrolyzer, the concept of using oxygen bubble generation to assist in removing hydrophobic organic products from electrode surface has been well documented in the reported system. And in the current work, the underlying mechanism remains qualitative and does not substantially advance fundamental understanding beyond prior studies on bubble-mediated cleaning or wettability engineering. Thus, the work does not meet the criteria for publication in high-level journal Nature Communications. My specific concerns are as follows.
1. The concept of coupling organic oxidation reactions with hydrogen generation is primarily intended to lower the electrolyzer voltage and thereby improve overall system efficiency. This strategy has been extensively reported and well established in the existing literature on paired electrolysis. In the present manuscript, it seems that the authors “overcomplicate the problem” by introducing constraints that are not intrinsic to the system, converting the soluble benzylamine to insoluble benzonitrile. I would like to say, it is not the fundamental problems that should be addressed in the paired system for hydrogen production as mentioned in the introduction part.
2. Although a current density of 200 mA cm-2 is demonstrated, the FE for BOR is only ~55% under these conditions, indicating substantial competition from OER. This compromises the claimed advantage of value-added paired electrolysis.
3. The TEA is based on idealized assumptions. Some other key industrial considerations (e.g., catalyst degradation, product purification cost, bubble management) should be considered.
4. The authors claim that bubble-induced cleaning is the key factor, yet unnecessarily rely on in situ OER to generate bubbles. This appears to be conceptually redundant, as direct gas bubbling should in principle provide the same effect without sacrificing selectivity or efficiency. The authors should consider and compare these two methods.
5. Is the bubble-assisted cleaning effect dependent on the specific wettability, roughness, or microstructure of the NiFeS electrode, or can it be generalized to flat or less structured electrodes?

Reviewer #2

(Remarks to the Author)
This work reported a bubble-assisted cleaning strategy for promoting primary amine electrooxidation, achieving a current density of 200 mA cm-2 with a faradaic efficiency of 55% at 1.65 V in a hybrid electrolyzer. The authors demonstrated that the generated oxygen bubbles during the OER process could take insoluble benzonitrile away from electrode surface and thus enable the re-exposure of active sites. This manuscript was well-performed, which provides some important insights for the development of integrated electrochemical energy platforms. However, the following issues require to be solved before it can be published:
1. 1H-NMR or 13C-NMR tests are suggested to characterize the oxidation products after electrolysis, as supportive evidences with the gas chromatography-mass spectrometry results.
2. The authors should provide more details about electrolysis conditions (e.g. duration, charge passed or solution volume).
3. In this work, the authors should clarify the potential side reactions and by-products of benzylamine electrooxidation.
4. The stability test lacks discussion of potential changes in morphology, structure or composition of catalyst after 40-hour

electrolysis. TEM, XRD or XPS characterizations are usually needed to confirm the structural stability of catalyst.
5. Error bars are needed in Figures 2e and 4c.
6. The author should evaluate the benzylamine electrooxidation performance of bare nickel foam and nickel felt.
7. It is better to investigate the HER performance when coupled with the BOR over an anion exchange membrane water electrolysis (AEMWE) device.

Reviewer #3

(Remarks to the Author)
Aiming to address the critical issues of electrode poisoning and limited current density caused by insoluble products in organic electro-oxidation coupled with hydrogen evolution reaction (HER), this study proposes a new bubble-assisted self-cleaning strategy, rather than the conventional strategy of catalyst surface modification. Taking benzylamine oxidation reaction (BOR) as the model reaction, the generated oxygen bubbles from the activated oxygen evolution reaction (OER) are utilized to timely desorb the insoluble product benzonitrile from the electrode surface. And the constructed AEMWE achieves a high current density of 200 mA/cm² with stable operation for 40 hours, demonstrating both academic innovation and industrial application potential. The research features a novel design concept and a complete experimental system, integrating in-situ observation and TEA, providing a new paradigm for the design of hybrid electrolysis systems. However, the manuscript still needs improvements in mechanism elaboration and data support. Revision is recommended before acceptance, and the specific revision suggestions are as follows.
Major comments:
1.The description in Figure 1b regarding the oil (BN) contact angle in air being close to 0°, as well as the dynamic process of Figure S4, are somewhat ambiguous as compared to the subsequent descriptions of super-hydrophilic and super-hydrophobic characteristics. The authors need to provide a clear explanation of the material properties.
2.For the discussion related to Figure 2，the authors only analyzed the electrode poisoning behavior caused by insoluble organic products through the variation of current density. It is expected to provide more evidence, such as the changes in Cdl or the impact on ECSA.
3.In Figure 3d, the discussion on the difference of bubble coalescence with and without oil film is overbrief. An in-depth analysis needs to be conducted. There is no Figure S17.
4.In the TEA calculation of the electrochemical reactor, there is no clear annotation of the price source for different materials. It is suggested to present the information with a table.

Minor comments:
1.For Figure 1b, the demonstrated value of the contact angle of the underwater oil droplet is 150° while the related description is over 160°.
2.There is no mention of Figure S5 in the main text.
3.It seems that the information in Note 2 of supplementary information is not related to the GC-MS information described in the text.
4.The tense used in the Note 3 of supplementary information is incorrect.
5.There are also several formatting issues in the manuscript, such as inconsistent annotation of current density units; incorrect annotation of electricity cost units in the TEA calculation; and ambiguous numerical expressions for income, expenditure and profit in the text.

Version 1:

Reviewer comments:

Reviewer #1

(Remarks to the Author)
The authors have addressed my concerns, and I therefore recommend its publication.

Reviewer #2

(Remarks to the Author)
The authors have addressed the concerns, and it is now suitable for publication.

Reviewer #3

(Remarks to the Author)
The authors have revised the manuscript well and it can be accepted now.

Open Access This Peer Review File is licensed under a Creative Commons Attribution 4.0 International License, which permits use, sharing, adaptation, distribution and reproduction in any medium or format, as long as you give appropriate credit to the original author(s) and the source, provide a link to the Creative Commons license, and indicate if changes were made.
In cases where reviewers are anonymous, credit should be given to 'Anonymous Referee' and the source.
The images or other third party material in this Peer Review File are included in the article's Creative Commons license, unless indicated otherwise in a credit line to the material. If material is not included in the article's Creative Commons license and your intended use is not permitted by statutory regulation or exceeds the permitted use, you will need to obtain permission directly from the copyright holder.
To view a copy of this license, visit https://creativecommons.org/licenses/by/4.0/

Dear Editor,

Thank you very much for your and the reviewers' hard work on our manuscript entitled "Bubble-assisted cleaning for primary amine oxidation towards ampere-level current densities" (#NCOMMS-24-55216-T). These comments are all valuable and very helpful for improving our work. We have carefully studied the comments and made revisions point by point.

The major revisions are summarized as follows,

1. The core concerns regarding the novelty of the fundamental concept have been carefully addressed and clarified.

2. The TEA analysis has been further deepened and elaborated.

3. The origin of the observed Faradaic efficiency has been clarified, and additional discussions on system optimization have been incorporated.

We have carefully revised the manuscript and Supplementary Information in accordance with the reviewers' comments, and all changes have been marked in the revised version for ease of reference. We appreciate for Editors/Reviewers' hard work earnestly, and hope that the revisions can meet the requirements for publication.

Yours sincerely
Liang Luo

Reviewer #1:

The manuscript reports a bubble-assisted method to lower electrode fouling for benzylamine electrooxidation coupled with hydrogen evolution. While the system demonstrates high current densities in AEM water electrolyzer, the concept of using oxygen bubble generation to assist in removing hydrophobic organic products from electrode surface has been well documented in the reported system. And in the current work, the underlying mechanism remains qualitative and does not substantially advance fundamental understanding beyond prior studies on bubble-mediated cleaning or wettability engineering. Thus, the work does not meet the criteria for publication in high-level journal Nature Communications. My specific concerns are as follows.

Response：

We appreciate the reviewer’s insightful and helpful comments on our manuscript. Your suggestions help us substantially improve the quality of the manuscript. For the major concern of fundamental understanding, during the reaction, insoluble organic products tend to continuously cover the electrode surface despite the use of oleophobic modifications on the electrode, particularly under high current densities. Therefore, implementing an in-situ bubble-assisted oil removal strategy is crucial for achieving continuous and efficient removal of organic residues and maintaining high-performance reaction operation.

1. The concept of coupling organic oxidation reactions with hydrogen generation is primarily intended to lower the electrolyzer voltage and thereby improve overall system efficiency. This strategy has been extensively reported and well established in the existing literature on paired electrolysis. In the present manuscript, it seems that the authors “overcomplicate the problem” by introducing constraints that are not intrinsic to the system, converting the soluble benzylamine to insoluble benzonitrile. I would like to say, it is not the fundamental problems that should be addressed in the paired system for

hydrogen production as mentioned in the introduction part.

Response: We thank the reviewer for pointing out this issue. We agree that coupling organic oxidation reactions with hydrogen generation as paired electrolysis is primarily intended to lower the electrolyzer voltage and thereby improve overall system efficiency. Current researches mainly focus on promoting the faradaic efficiency (FE).

(1) However, as the demand of hydrogen production dramatically increases, the installed capacity of electrolyzers is projected to surge, with the total installed power expected to reach 49 Mtpa by 2030. Such substantial installed capacity inevitably necessitates the coupling of high-current anodic reactions (Joule, 2025, 9, 102049). However, most of the paired electrolysis systems still suffer from the "problem" of rather limited low anodic current density (typical range of 50-100 mA/cm^2), which reversely compromises the increasingly hydrogen production, owing to the sluggish reaction kinetics and poor mass transfer. Thus, the FE of paired electrolysis would not be the top priority for the applications. The added value should be comprehensively considered.

(2) Secondly, the subsequent purification of aqueous products becomes the major part of total cost for the scaling-up application of conventional anodic electrochemical organic reactions, commonly higher than the energy consumption of electrochemical reactions. For instance, in glycerol oxidation, the purification cost of products like glycolic acid (GA) commonly reaches up to 60-70% of the total cost, as shown in Figures R1a, b, c (Nat. Catal., 2024, 7, 330). The cost of purification could be calculated as follow,

$$\begin{aligned}\text{purification cost} &= \text{Distillation column cost} + \text{purification material costs} \\ &+ \text{seperations energy cost} \\ &= 58.6\% \times 0.18\ \$/\text{kg GA} + 81.9\% \times 1.24\ \$/\text{kg GA} \\ &+ 65.4\% \times 0.77\ \$/\text{kg GA} = 1.62\ \$/\text{kg GA}\end{aligned}$$

The proportion of the purification cost within the total cost could be 73.8% according to the calculation,

$$\frac{1.62\ \$/\text{kg GA}}{2.2\ \$/\text{kg GA}} = 73.8\%$$

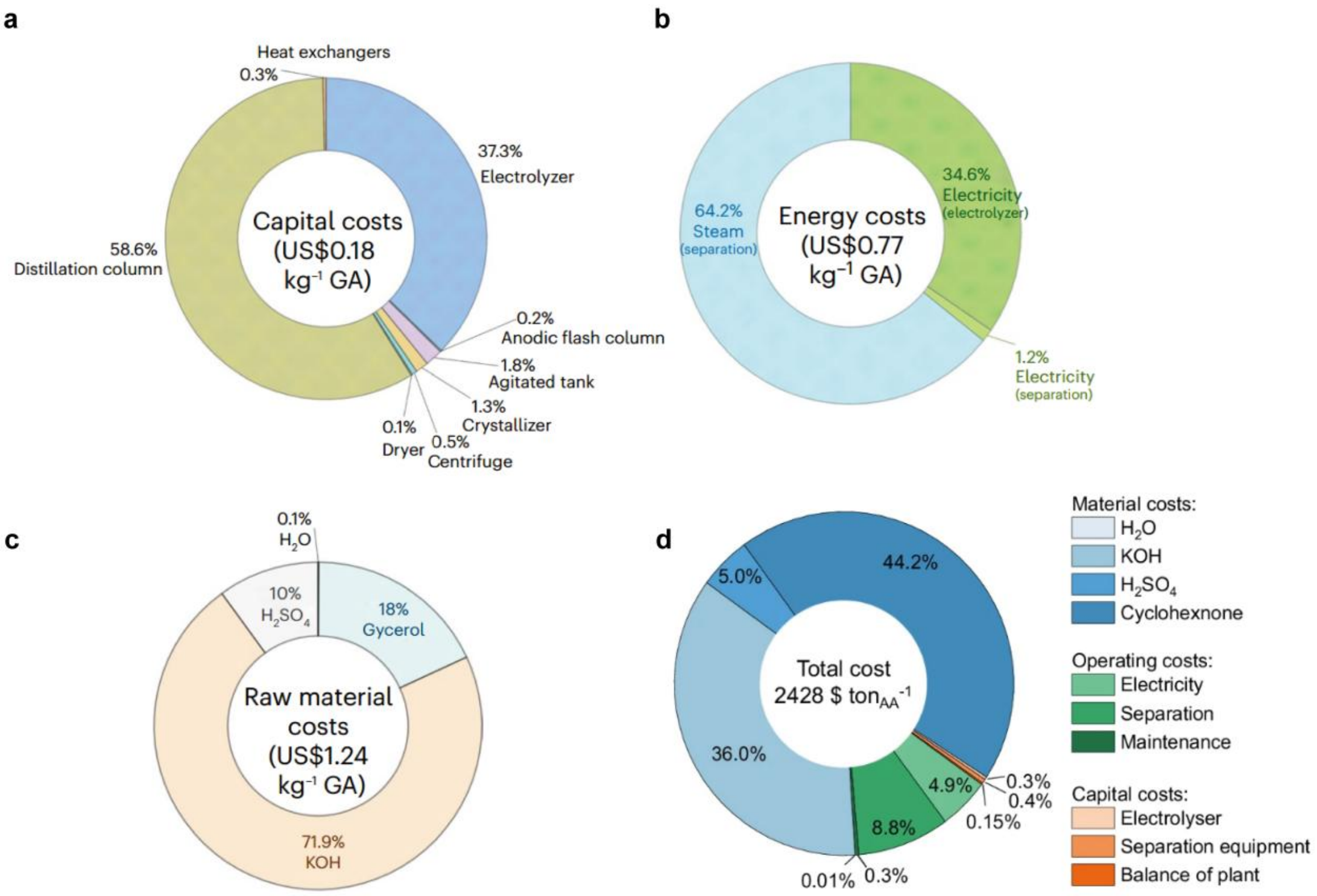

Figure R1. (a) Capital costs, (b) Energy cost, (c) Raw material costs of the glycolic acid electrochemical process under the base case scenario. (d) Total cost of the adipic acid electrochemical process.

Similarly, as shown in Figure R1d, the proportion of the purification cost of cyclohexanone oxidation reaches 50.2% (Nat. Commun., 2024, 15, 7685),

$$\begin{aligned} &\text{purification cost} \\ &\quad = \text{purification material cost} + \text{seperation costs} \\ &\quad + \text{seperations equipment cost} = (36.0\% + 5.0\%) + 8.8\% + 0.4\% \\ &\quad = 50.2\% \end{aligned}$$

(3) In addition, for the development of anodic organic oxidation reactions involving phase-transition themselves, not limited to the oxidation of benzylamine to benzonitrile, the electrode fouling due to the product insolubility remains a critical yet challenging “problem” for paired electrolysis. Even though there have been some reported strategies by building oleophobic electrode surfaces (Adv. Funct. Mater. 2018, 28, 1800712), the long-term operation still remains challenging after the inevitable invalidation of wettability.

Hence, based on the comprehensive consideration, with the aim of addressing this problem towards practical applications, we introduce the reactions involving water insoluble phases, which can significantly reduce the cost of separation and purification while maintaining excellent selectivity. Such reactions can be designed as optional modular reactors to flexibly align with practical scalable applications and realize the comprehensive product value enhancement, since stable and efficient operation can be successfully achieved. We believe that introducing such phase-transformation hybrid electrolysis within bubble-assisted strategy provides a potential route for the design of next-generation integrated electrochemical platforms.

2. Although a current density of 200 mA cm^{-2} is demonstrated, the FE for BOR is only ~55% under these conditions, indicating substantial competition from OER. This compromises the claimed advantage of value-added paired electrolysis.

Response： Thank you very much for the point. It is a deliberate productivity-selectivity trade-off.

Throughput outweighs efficiency: the BOR can achieve nearly 100% FE at 1.40 V with rather limited current density (<25 mA/cm^2), which, however, becomes the limitation for hydrogen production. The total revenue consists of the produced cathodic hydrogen and the anodic product BN. Our technical approach involves modifying the anodic reaction to obtain higher-value organic products without affecting the normal hydrogen production mode of the electrolyzer. Notably, as combined with the BOR, both the hydrogen production efficiency can be effectively promoted, and the BN produced from the anodic reaction represent non-negligible sources of profit, as illustrated in the TEA analysis. Here, activating OER at 1.65 V is not merely a "parasitic loss" but a necessary functional tool. Sacrificing partial FE to sustain high-rate, continuous operation for long-term operation is necessary in practical applications.

The TEA results show that, the total cost and revenue are $408.63 and $585.27, respectively, indicating that the 1.65 V top the profit of $176.64 with a fixed working area of 10 m^2. The revenue contributions are $53.19 from hydrogen and $167 from BN (Figure 4e).

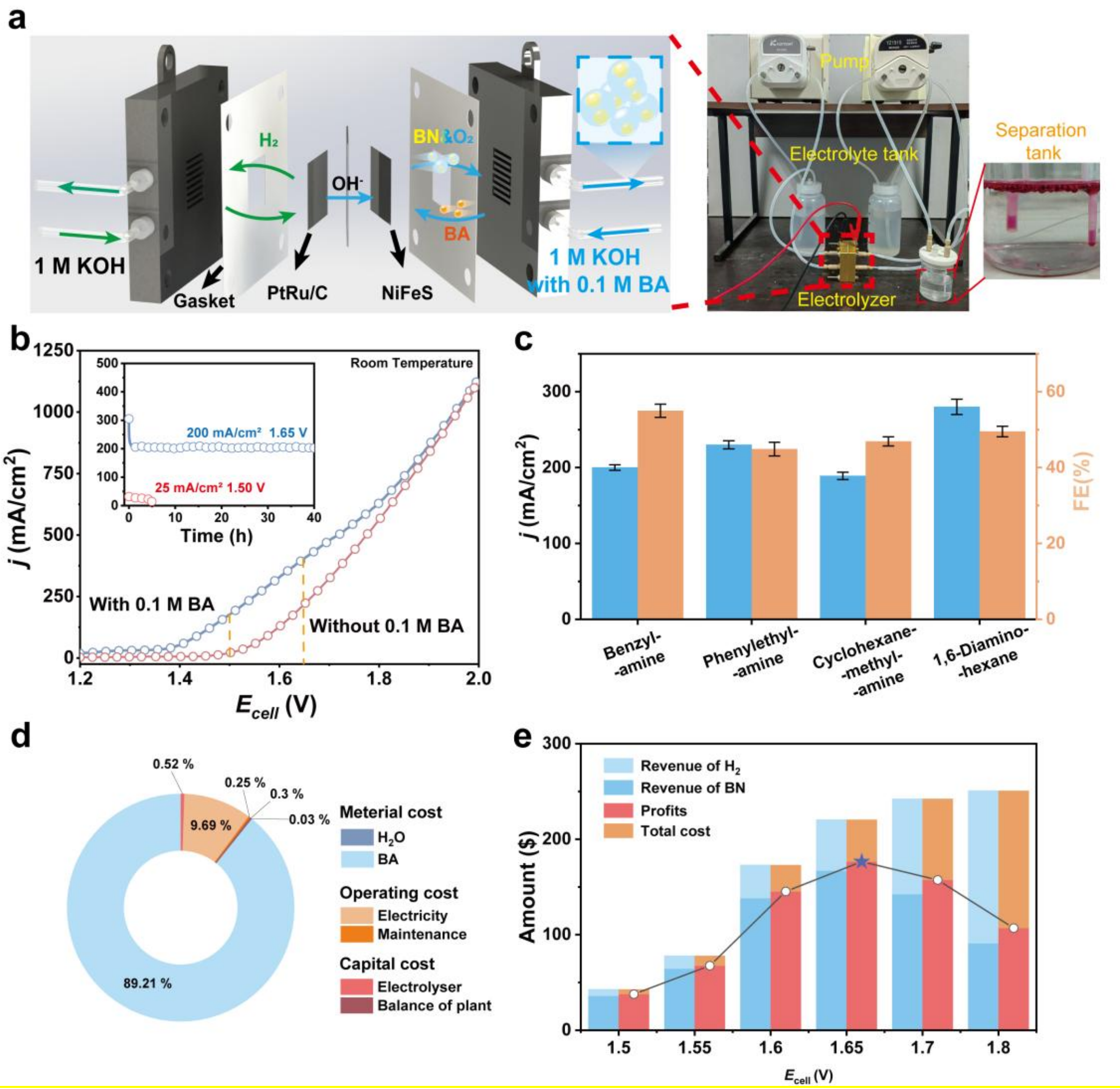

Figure 4. a) Schematic and operating schematic diagram of the AEMWE. b) LSV curves of with and without BA precursor based on a 2×2 cm^2 device. Inset: stability tests. c) Substrate scope of the electrocatalysis of primary amines into nitriles over the catalyst. d) Total cost of the BN electrochemical process at conditions of 55%, current density of 200 mA/cm^2, and cell voltage of 1.65 V. e) The revenues and profits in different operating conditions.

3. The TEA is based on idealized assumptions. Some other key industrial considerations (e.g., catalyst degradation, product purification cost, bubble management) should be considered.

Response： We sincerely appreciate the reviewer's suggestion for the concern

on TEA. As suggested, the related key considerations have been added.

(1) Catalyst cost: Our NiFeS catalyst is PGM-free catalyst. With a catalyst loading of 4.5 mg/cm^2, the calculated catalyst cost is only 0.0052 \$/cm^2, which accounts for a nearly negligible proportion in the TEA and is expected to be further reduced in large-scale production.

(2) Zero-energy purification: A nonnegligible economic advantage of our insoluble product characteristics lies in the spontaneous phase separation. Benzonitrile naturally stratifies from the aqueous electrolyte without the need for energy-intensive downstream processing (e.g., distillation or extraction). For instance, in glycerol oxidation, the analysis reveals that downstream product purification alone accounts for over 60% of capital costs (Nat. Catal., 2024, 7, 330). These substantial economic penalties, are often neglected in catalyst-focused studies. Our system enables spontaneous phase separation of insoluble benzonitrile, which directly circumvents these costly and energy-intensive purification steps, thereby offering a clear route towards economically sustainable electrosynthesis.

(3) Bubble management: In our case, the in situ generated oxygen bubbles effectively serve a dual role as both an in-situ cleaning agent and a flotation medium for oily product separation, thereby entirely eliminating the need for external gas pumping or mechanical agitation. The associated energy consumption of activating OER has been systematically quantified in the cost. The cost associated with product purification in typical aqueous-phase reactions is much higher (Nat. Catal., 2024, 7, 330). This highlights a key advantage of generating insoluble products from aqueous reactants, as the associated OER energy cost is negligible relative to the overall process.

We have added additional details and annotations for the TEA calculations in Supplementary Information.

Revisions in the manuscript:

The TEA results show that, the total cost and revenue are \$408.63 and \$585.27, respectively, indicating the profit of \$176.64 with a fixed working area of 10 m^2

(current density of 200 mA/cm^2, cell voltage of 1.65 V, FE of 55%). In addition, the in-situ generated oxygen bubbles via OER serve as intrinsic bubble-assisted cleaning and oily product flotation carriers. More importantly, with the efficient bubble-assisted removal, such unique water insoluble product inversely saves the extra purification treatment and related costs, which is usually over 50% for conventional overall aqueous paired electrolysis systems.

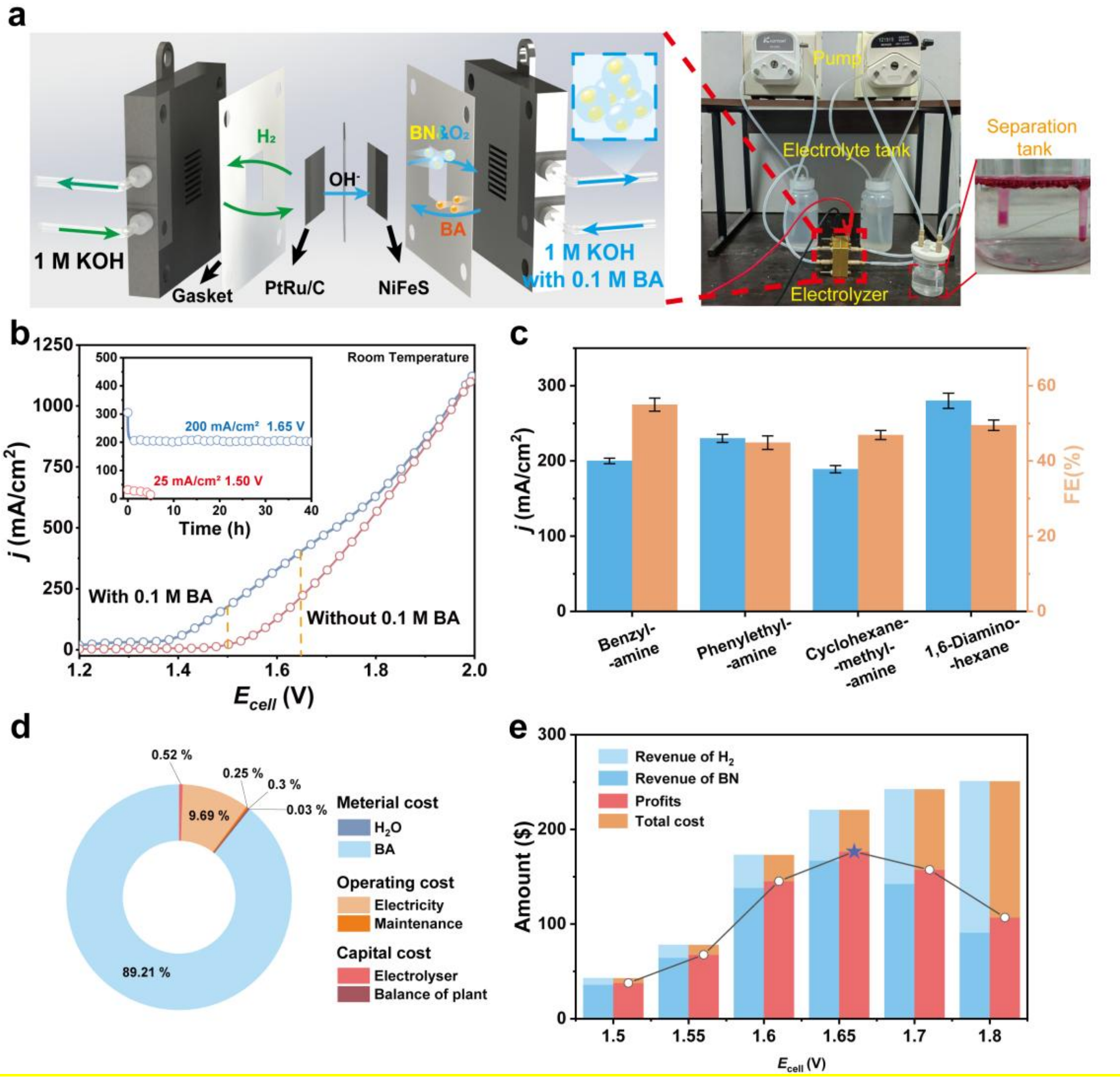

Figure 4. a) Schematic and operating schematic diagram of the AEMWE. b) LSV curves of with and without BA precursor based on a 2×2 cm^2 device. Inset: stability tests. c) Substrate scope of the electrocatalysis of primary amines into nitriles over the catalyst. d) Total cost of the BN electrochemical process at conditions of 55%, current density of 200 mA/cm^2, and cell voltage of 1.65 V. e) The revenues and profits in different operating conditions.

Revisions in the Supplementary Information:

Operating cost:

1. Electricity:

$$33\ kW \times 24\ h\ \times 0.05\ \$\ /(kW \cdot h) = 39.6\ \$ \tag{15}$$

2. Maintenance of electrolyzer:

$$10.0\ m^2 \times 1485\ \$/m^2\ \times 0.025 \div 350\ day = 1.03\ \$ \tag{16}$$

4. The authors claim that bubble-induced cleaning is the key factor, yet unnecessarily rely on in situ OER to generate bubbles. This appears to be conceptually redundant, as direct gas bubbling should in principle provide the same effect without sacrificing selectivity or efficiency. The authors should consider and compare these two methods.

Response: Thank you very much for the point. We totally agree that selectivity and efficiency are very important. As suggested, we have conducted the comparison of the two methods, listed as follows:

(1) Direct gas bubbling is a cleaning method, but would be challenging in this case. On one hand, the introduction of external bubbles can hardly clean the entire reactive area, and even leads to extra bubble-induced ohmic resistance owing to the bubble retention within the porous transport electrode. On the other hand, as demonstrated in the preliminary research (Figure 1d), there is a dramatic current drop over 70% within the first 50 seconds at 1.4 V, indicating that the adhesion of just a small amount of oily product BN on electrode surface would block massive active sites. Hence, even without taking the above-mentioned cleaning effect into account, continuous bubbling is required since the intermittent operation would not remove the oily product timely. This would inversely require a lot of energy input.

(2) The in situ electrochemically generated bubbles can offer precise control for oily product removal. Owing to the surface tension of oil-gas interface is lower than that of oil-water interface, the generated bubbles tend to merge with oily product, and thereby effectively and timely take them away from electrode surface. Moreover, such anodic reaction produces high-purity benzonitrile, and the activation of OER does not generate any additional by-products, as evidenced by the GC-MS results in Figure R2.

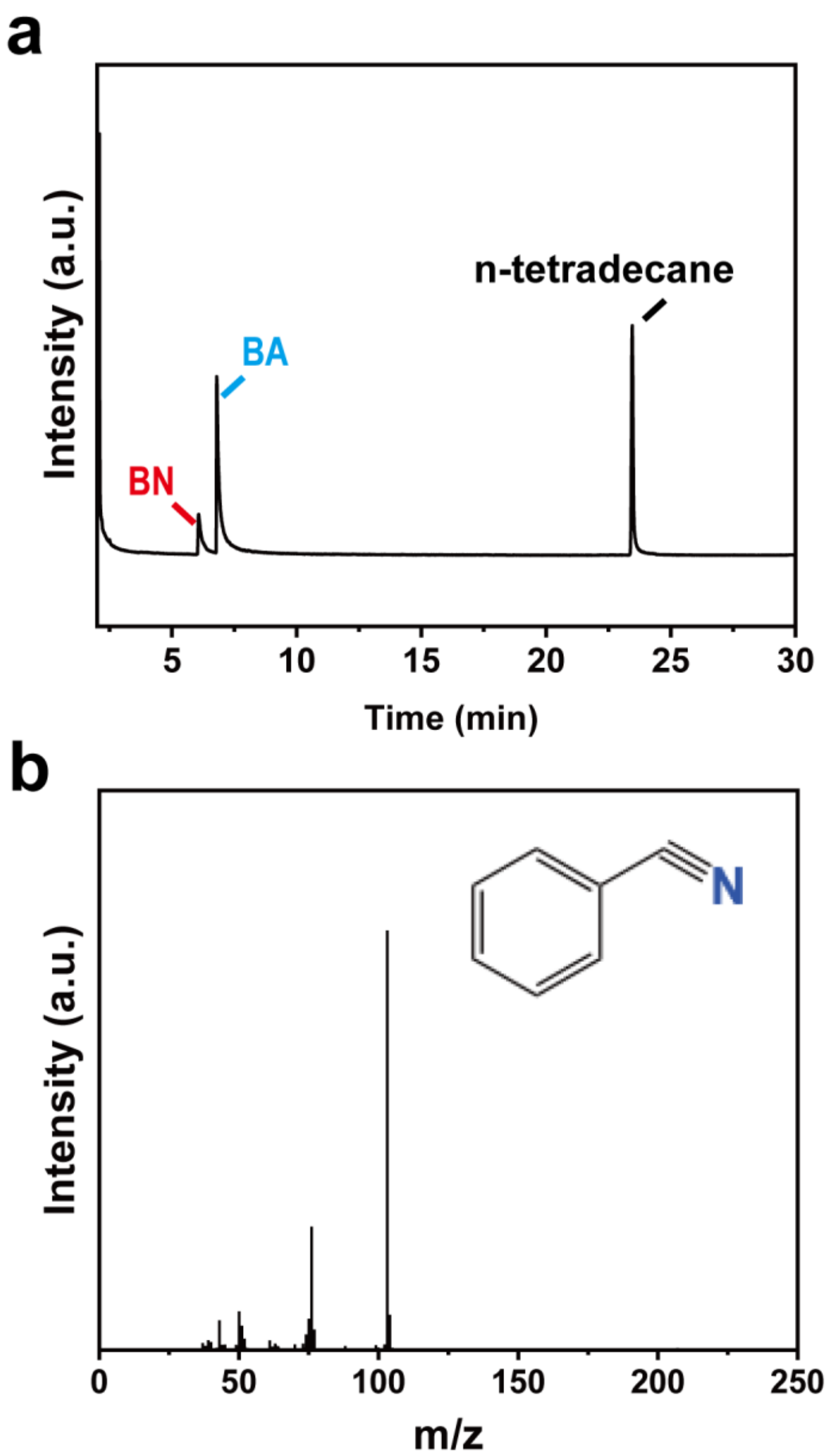

Figure R2. (a) GC chromatogram of the product. (b) MS spectrum at retention time of 6.071 min.

(3) Based on the calculations for comment 3, the energy cost associated with the in situ OER accounts for only a small fraction of the total process, while there are no additional purification capital or material costs involved.

5. Is the bubble-assisted cleaning effect dependent on the specific wettability, roughness, or microstructure of the NiFeS electrode, or can it be generalized to flat or less structured electrodes?

Response: Thank you very much for the point. Bubble-assisted cleaning effect is a universal thermodynamic phenomenon, not dependent on the specific

wettability, roughness or microstructure of the NiFeS electrode.

To verify such statement, apart from the demonstrated scenario of nanoarrayed NiFeS catalyst in the main text, we also observed the identical bubble-nucleation and oil removal phenomena on bare, unstructured nickel mesh (Figure S17). Typically, by activating the OER (Figure R3), the bubble nucleation occurs near the oil droplet, the total interface energy (E_1) of bubble and oil droplet is,

$$E_1 = 4\pi r_o^2 \gamma_{ow} + 4\pi r_g^2 \gamma_{gw} \tag{5}$$

where r_o and r_g are the radius of oil droplet and bubble, respectively. γ_{ow} and γ_{gw} are the oil-water interface tension and gas-water interface tension, respectively. When the bubble grows big enough with the contact with the oil droplet, the bubble tends to merge with the oil droplet, and the total interface energy (E_2) varies as,

$$E_2 = 4\pi (r_o^3 + r_g^3)^{2/3} \gamma_{gw} + 4\pi r_o^2 \gamma_{og} \tag{6}$$

where γ_{og} is the oil-gas interface tension, the volume of oil droplet is enlarged by bubble, the limited change in bubble volume can be ignored in this process.

$$(r_o^3 + r_g^3)^{\frac{2}{3}} \approx r_g^2 \tag{7}$$

Thus, the difference of total interface energy (ΔE) can be calculated by merging Equations (5), (6), and (7),

$$\Delta E = 4\pi r_o^2 (\gamma_{og} - \gamma_{ow}) \tag{8}$$

Since the surface tension of oil-gas interface is lower than that of oil-water interface, namely,

$$\gamma_{og} - \gamma_{ow} < 0 \tag{9}$$

such merging phenomenon would happen spontaneously, leading to generalized phenomena for flat or less structured electrodes.

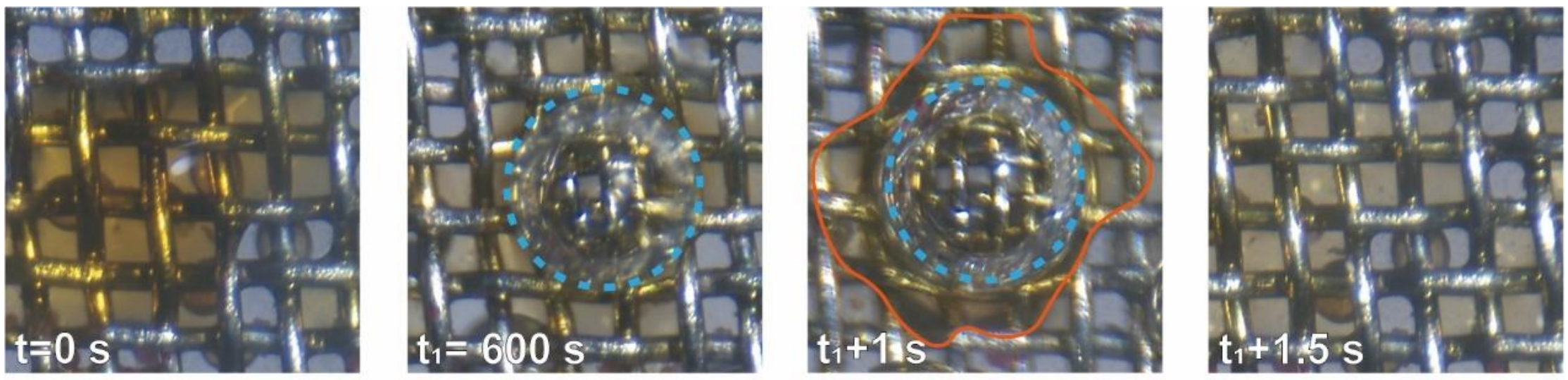

Figure S17. The process of the bubble-assisted oil droplet cleaning on bare nickel mesh.

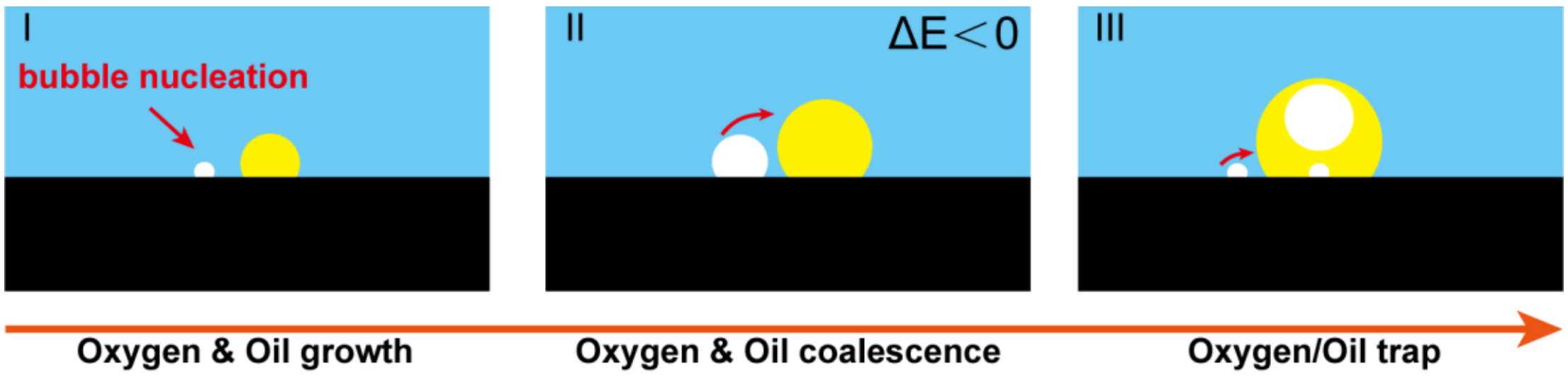

Figure R3. Evolution of bubble-assisted product removal on electrodes surface (I~III).

Reviewer #2

this work reported a bubble-assisted cleaning strategy for promoting primary amine electrooxidation, achieving a current density of 200 mA cm^{-2} with a faradaic efficiency of 55% at 1.65 V in a hybrid electrolyzer. The authors demonstrated that the generated oxygen bubbles during the OER process could take insoluble benzonitrile away from electrode surface and thus enable the re-exposure of active sites. This manuscript was well-performed, which provides some important insights for the development of integrated electrochemical energy platforms. However, the following issues require to be solved before it can be published:

Response: We appreciate the reviewer's recognition and constructive feedback of our work. We have carefully gone through all the points, and made the revisions point by point.

1. *^{1}H-NMR or ^{13}C-NMR tests are suggested to characterize the oxidation products after electrolysis, as supportive evidences with the gas chromatography-mass spectrometry results.*

Response: We appreciate the suggestion to include NMR characterization. As suggested, we have performed ^{1}H NMR analyses to further confirm the identity of the oxidation products, providing complementary evidence to our GC-MS results.

The ^{1}H NMR spectrum (Figure R4) shows only typical signals of product benzonitrile (BN), trace reactant BA (benzylamine), and the residual solvent ethyl acetate. The aromatic region (δ~7.2–8.1 ppm) exhibits multiple overlapping peaks, which are consistent with benzonitrile but partially overlap with benzylamine signals. In addition, characteristic solvent peaks are also observed. Importantly, no signals corresponding to benzaldehyde (~10 ppm) or benzoic acid (~12 ppm) are detected, indicating the absence of by-products.

Due to the peak overlap in the aromatic region and solvent interference, we further performed GC-MS analysis and NMR. As shown in Figure R2, a peak

corresponding to the molecular ion of benzonitrile is observed at m/z=103 in the mass spectrum, with no detectable by-products. NMR further confirms the absence of over-oxidation products, while GC-MS unambiguously verifies that benzonitrile is the sole organic product. The corresponding GC-MS data have been added to the Supplementary Information, and the manuscript has been revised accordingly.

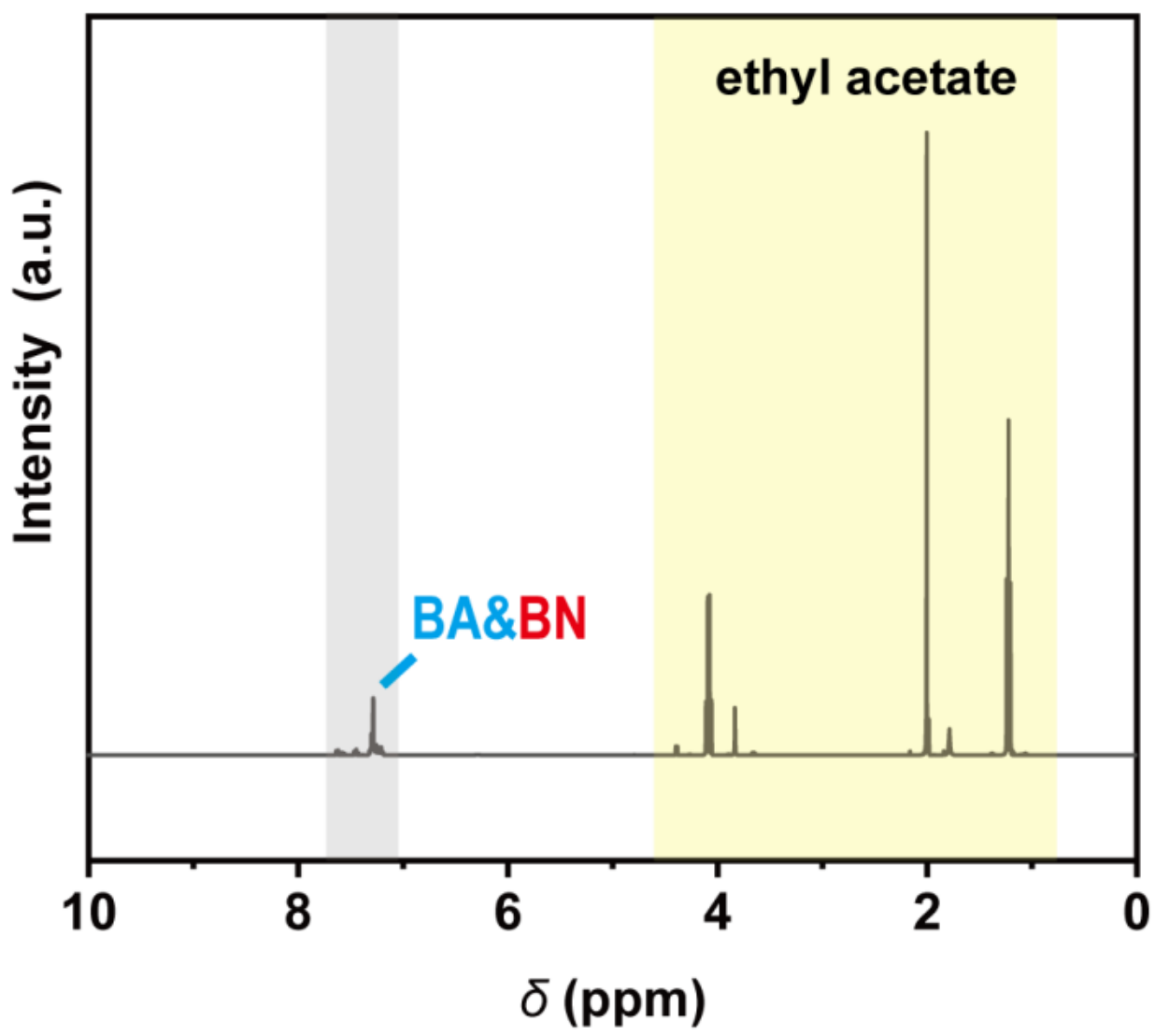

Figure R4. ^{1}H NMR spectrum of the electrolysis product after extraction

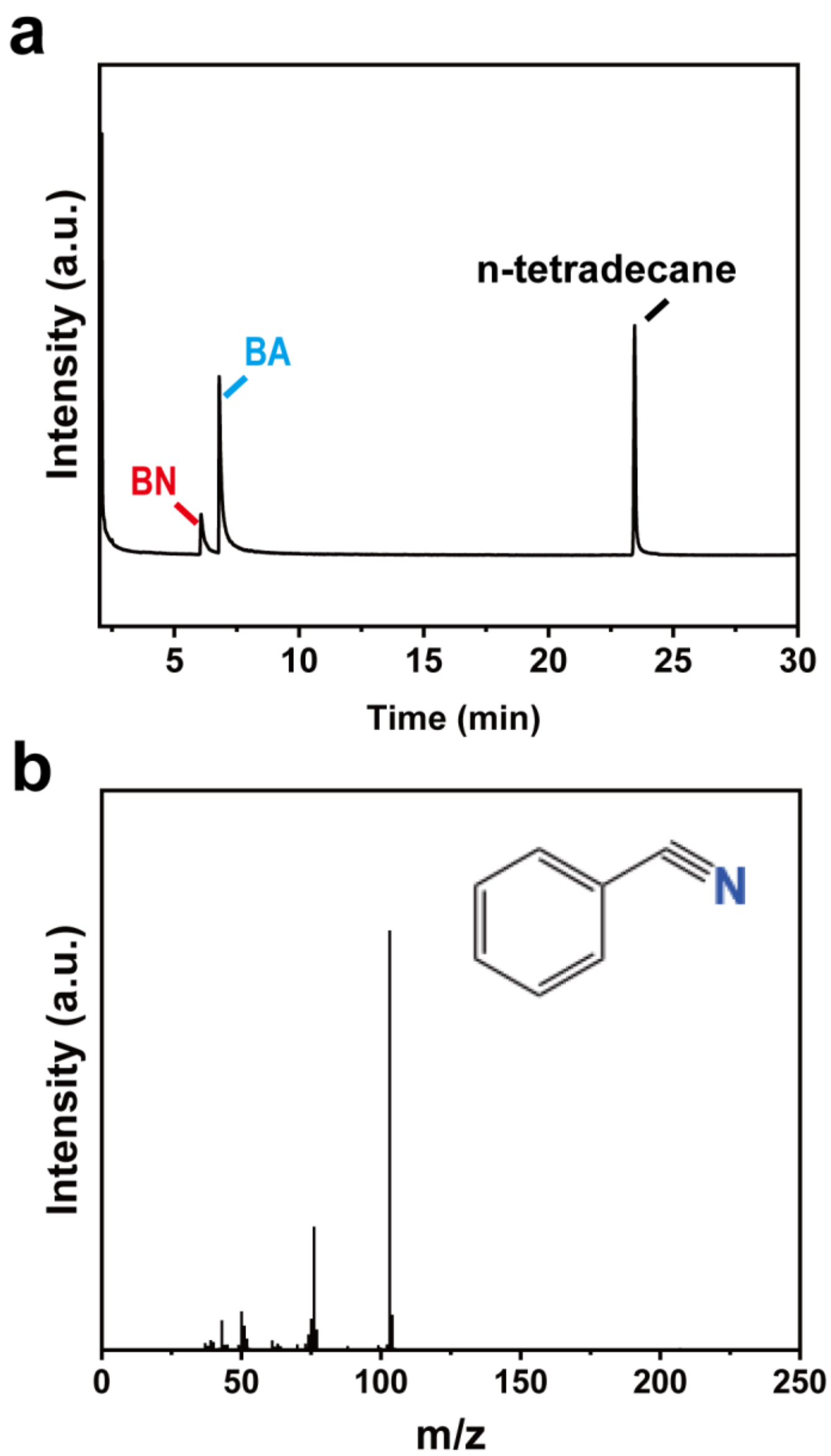

Figure R2. (a) GC chromatogram of the reaction mixture. (b) MS spectrum at retention time 6.071 min

Revisions in the manuscript:

Besides, the NiFeS catalyst grown on nickel foam exhibits a similar performance trend, delivering a current density of 450 mA/cm^2 in 0.1 M BA at 1.42 ± 0.04 V vs. RHE, compares to 75.0 mA/cm^2 of bare substrate (Figure S8). GC-MS analysis confirms that no detectable organic by-products, with average faradaic efficiency over 95% for BOR (Figures S9–S12).

2. The authors should provide more details about electrolysis conditions (e.g. duration, charge passed or solution volume).

Response： We thank the reviewer for pointing out the lack of detail regarding the electrolysis conditions for the electrolysis. As suggested, we have added the related details in the revised manuscript and Supplementary Information.

Revisions in the manuscript:

The as-prepared NiFeS@nickel felt catalyst exhibits an OER onset potential at approximately 1.5 V vs. RHE in 50 mL 1 M KOH, with a pre-oxidation peak of Ni active species (Ni^{2+} to Ni^{3+}) at 1.35 V (Figure 1c).

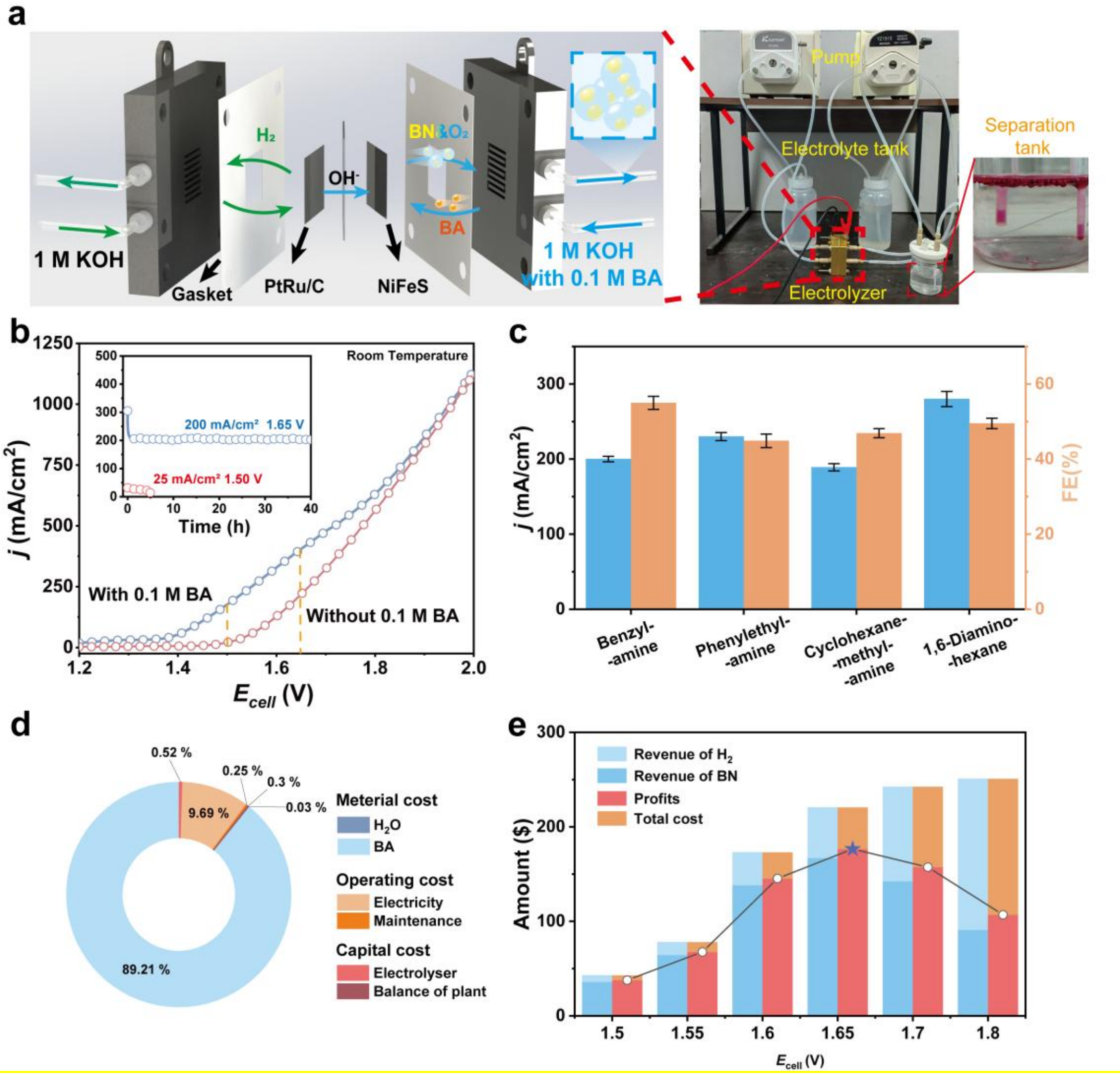

Figure 4. a) Schematic and operating schematic diagram of the AEMWE. b) LSV curves of with and without BA precursor based on a 2×2 cm^2 device. Inset: stability tests. c) Substrate scope of the electrocatalysis of primary amines into nitriles over the catalyst. d) Total cost of the BN electrochemical process at conditions of 55%, current density of 200 mA/cm^2, and cell voltage of 1.65 V. e) The revenues and profits in the different operating condition.

Revisions in the Supplementary Information:

Electrochemical measurements. Electrochemical experiments for hydrogen

evolution reaction (HER) and benzylamine oxidation reaction (BOR) were carried out in a standard three-electrode cell. The as-prepared catalysts were used as the working electrodes. Graphite rod and Hg/HgO (1 M KOH) were used as the counter and reference electrodes, respectively. The potentials in experiments were referred to the reversible hydrogen electrode (RHE). Electrochemical tests were conducted in 1.0 M KOH with/without 0.1 M benzylamine solution. The linear sweep voltammetry (LSV) measurements were conducted at a scan rate of 5 mV/s. Chronopotentiometry test of BOR was conducted in 200 mL 1 M KOH solution containing 0.1 M BA under vigorous stirring.

ECSA measurements. After full electrode activation, the potential range was adjusted to the non-Faradaic region (100 mV window) to ensure no electrochemical reactions occurred. CV measurements were performed at a scan rate of 50 mV/s until the CV curves became completely overlapped. Subsequently, CV tests were conducted at various scan rates of 20, 40, 60, 80, and 100 mV/s. The current density at the mid-potential was recorded for each scan rate. The C_{dl} was determined from the slope of the linear plot of current density versus scan rate. The ECSA was then calculated using a standard specific capacitance value of 40 μF/cm^2.

Device setup. The AEM device was assembled with NiFeS as anode catalyst and PtRu/C as cathode catalyst. The cathode and anode compartments were separately filled with 1 L KOH solution in the cathode compartment and 5 L KOH with BA in the anode compartment.

3. *In this work, the authors should clarify the potential side reactions and by-products of benzylamine electrooxidation.*

Response：

We thank the reviewer for highlighting the need to clarify potential side reactions and by-products during benzylamine electrooxidation. As discussed in our response to Comment 1, GC-MS analysis reveals a single dominant peak

corresponding to benzonitrile, with no other detectable organic by-products, indicating high selectivity under our experimental conditions.

While ¹H NMR shows some signal overlap between benzylamine and benzonitrile in the aromatic region, it does not affect the overall conclusion when combined with GC-MS results.

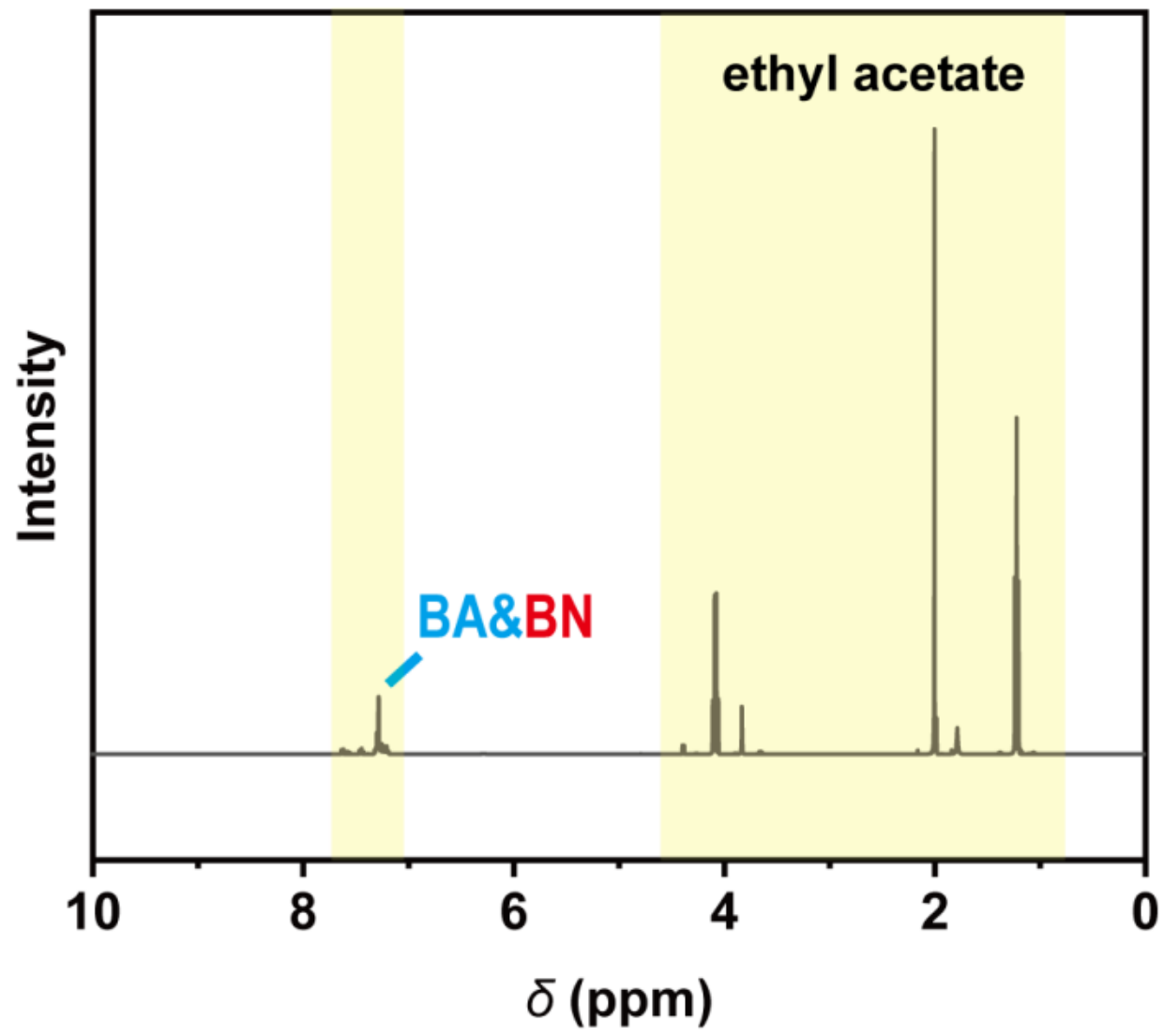

Figure R4. ¹H NMR spectrum of the electrolysis product after extraction

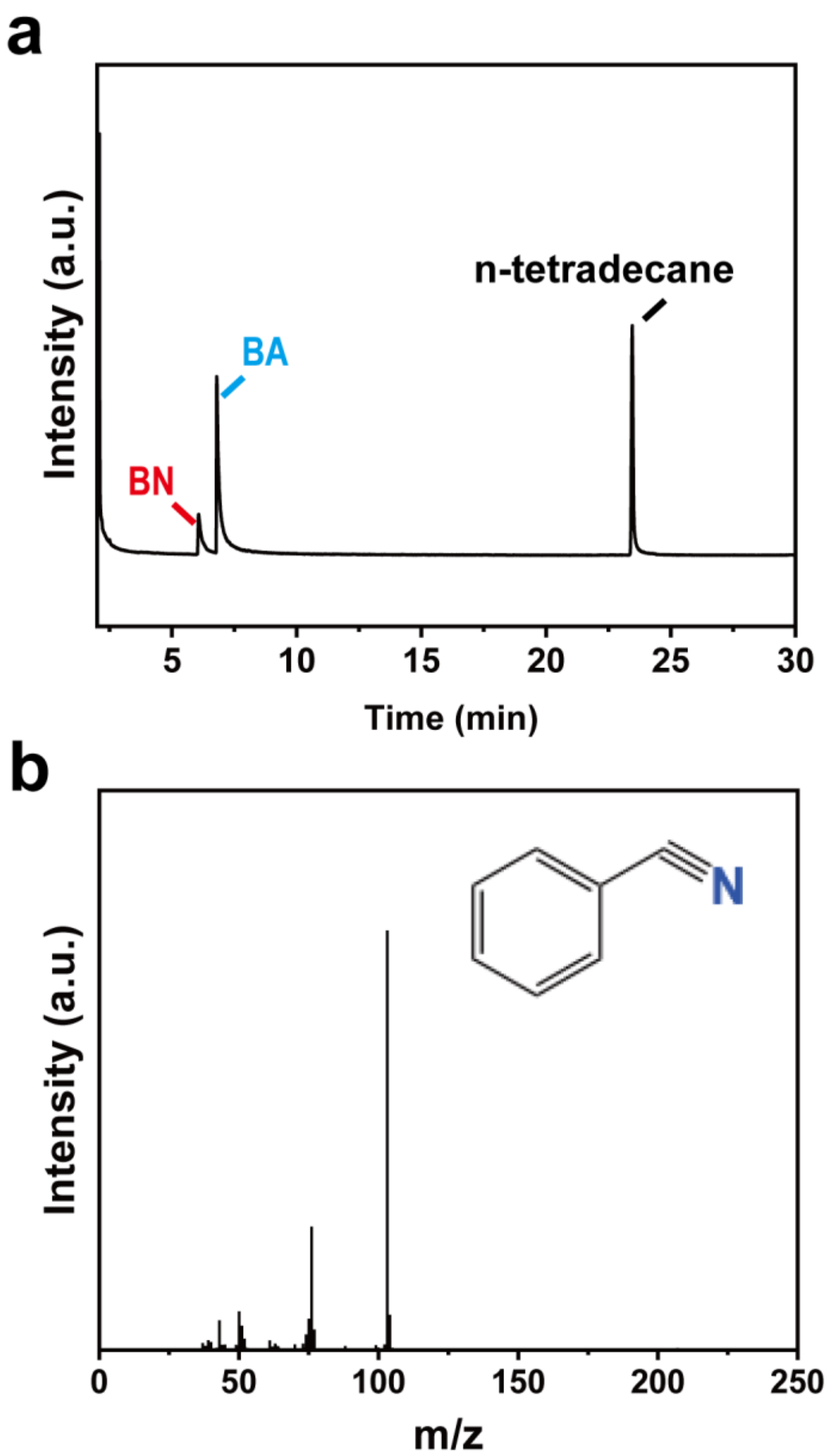

Figure R2. (a) GC chromatogram of the reaction mixture. (b) MS spectrum at retention time 6.071 min

These results collectively confirm that no significant side reactions occur, and benzonitrile is the exclusive organic product. The corresponding discussion has been clarified in the manuscript.

Revisions in the manuscript:

Besides, the NiFeS catalyst grown on nickel foam exhibits a similar performance trend, delivering a current density of 450 mA/cm^2 in 0.1 M BA at 1.42 ± 0.04 V vs. RHE, compares to 75.0 mA/cm^2 of bare substrate (Figure S8). GC-MS analysis confirms that no detectable organic by-products, with average faradaic efficiency over 95% for BOR (Figures S9–S12).

4. The stability test lacks discussion of potential changes in morphology, structure or composition of catalyst after 40-hour electrolysis. TEM, XRD or XPS characterizations are usually needed to confirm the structural stability of catalyst.

Response：

To examine the catalyst evolution after long-term electrolysis, we performed XPS characterization of the NiFeS catalyst after 40 h operation, and the results are shown in Figure R3. By comparing the spectra before and after electrolysis, the Ni 2p and Fe 2p signal remains clearly observable with a similar characteristic binding energy, indicating that Ni-containing surface species are still retained after prolonged operation. In contrast, the S 2p region, although the signal intensity decreases markedly after 40 h electrolysis, sulfur-related signals are still detectable, indicating that sulfur species are partially lost but not completely removed from the catalyst surface.

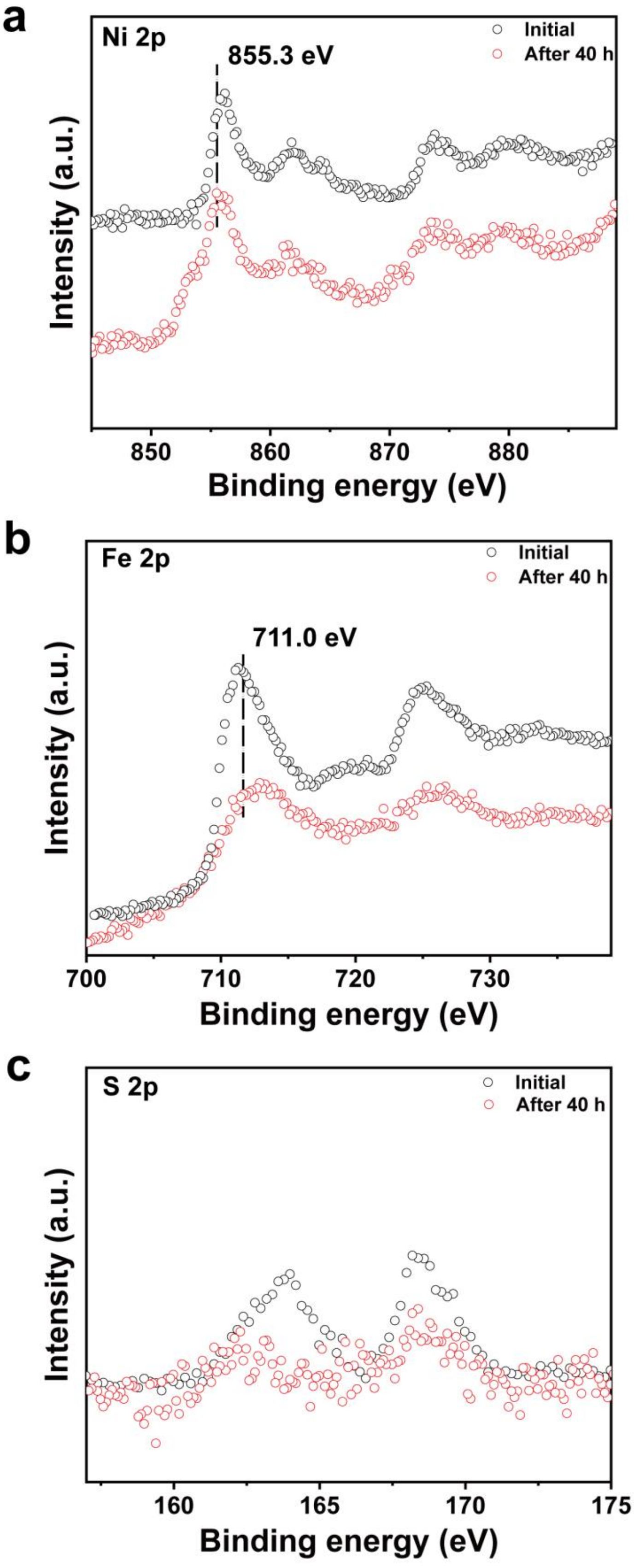

Figure R5. XPS spectra of the NiFeS catalyst before and after 40 h electrolysis: (a) Ni 2p, (b) Fe 2p, and (c) S 2p regions.

We have supplemented the XRD testing, as shown in Figure R6. Strong peaks appear at 44.3°, 51.5°, and 75.9° in the spectra, which correspond to diffraction signals from the nickel felt substrate. We could only identify the Ni_3S_2 species through the signals at 31.0° and 50.1°, in combination with the XPS data.

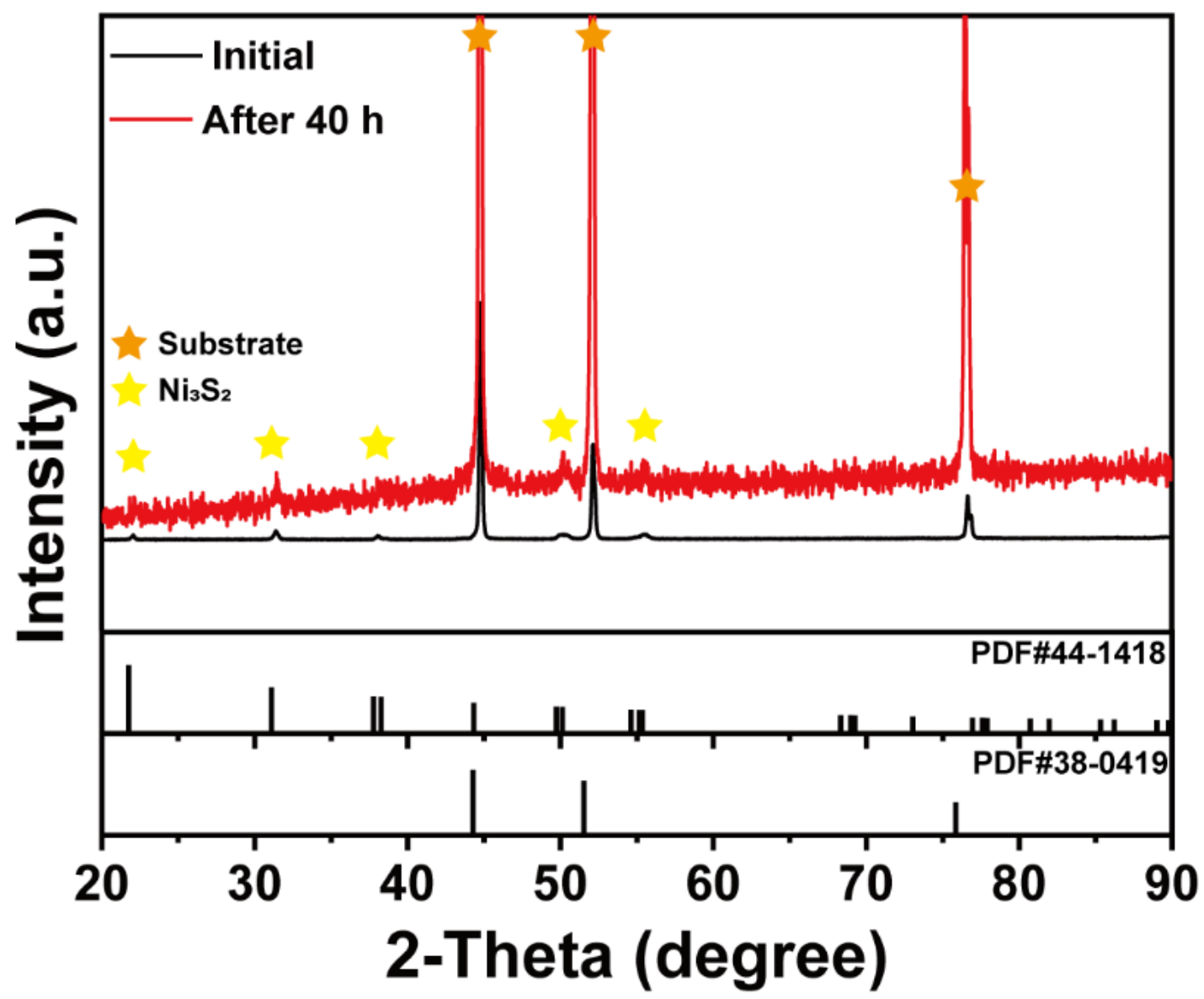

Figure R6. XRD pattern of the NiFeS catalyst after 40 h electrolysis

Revisions in the manuscript:

The current density at the potential of 1.50 V (without OER) stabilizes eventually at 25 mA/cm^2. In contrast, after the initial decrease due to the balance between the oily product generation and bubble-assisted removal, the current density at the potential of 1.65 V could be maintained at 200 mA/cm^2 for over 40 h, demonstrating a superior stability and performance as well. After the operation, the Ni 2p and Fe 2p signals remain clearly observed at 855.3 eV and 711.0 eV by XPS data, and the Ni_3S_2 species could be identified by XRD data, indicating that the surface species are still retained after prolonged operation. (Figures S19 and S20).

5. *Error bars are needed in Figures 2e and 4c.*

Response： Thank you very much for the point. Error bars have been added to Figures 2e and 4c in the revised manuscript. The values shown in these figures were obtained from repeated independent experiments, and the error bars

represent the variation among these measurements. These additions provide a more rigorous presentation of the FE and yield data and the substrate-scope results.

Revisions in the manuscript:

The results indicate that the catalyst achieves a nearly 100% FE at a potential of 1.40 V, with a yield of 0.55 mmol/h (Figure 2e). As the anodic potential increases, the OER becomes increasingly competitive, leading to a decrease in FE of BOR. The yield of BN rises gradually from 0.55 mmol/h to 0.71 mmol/h as the potential reaches 1.50 V, corresponding to an FE of 48.6%, reflecting competing OER activation. Beyond 1.55 V, the dominant OER decreases both BN yield and FE.

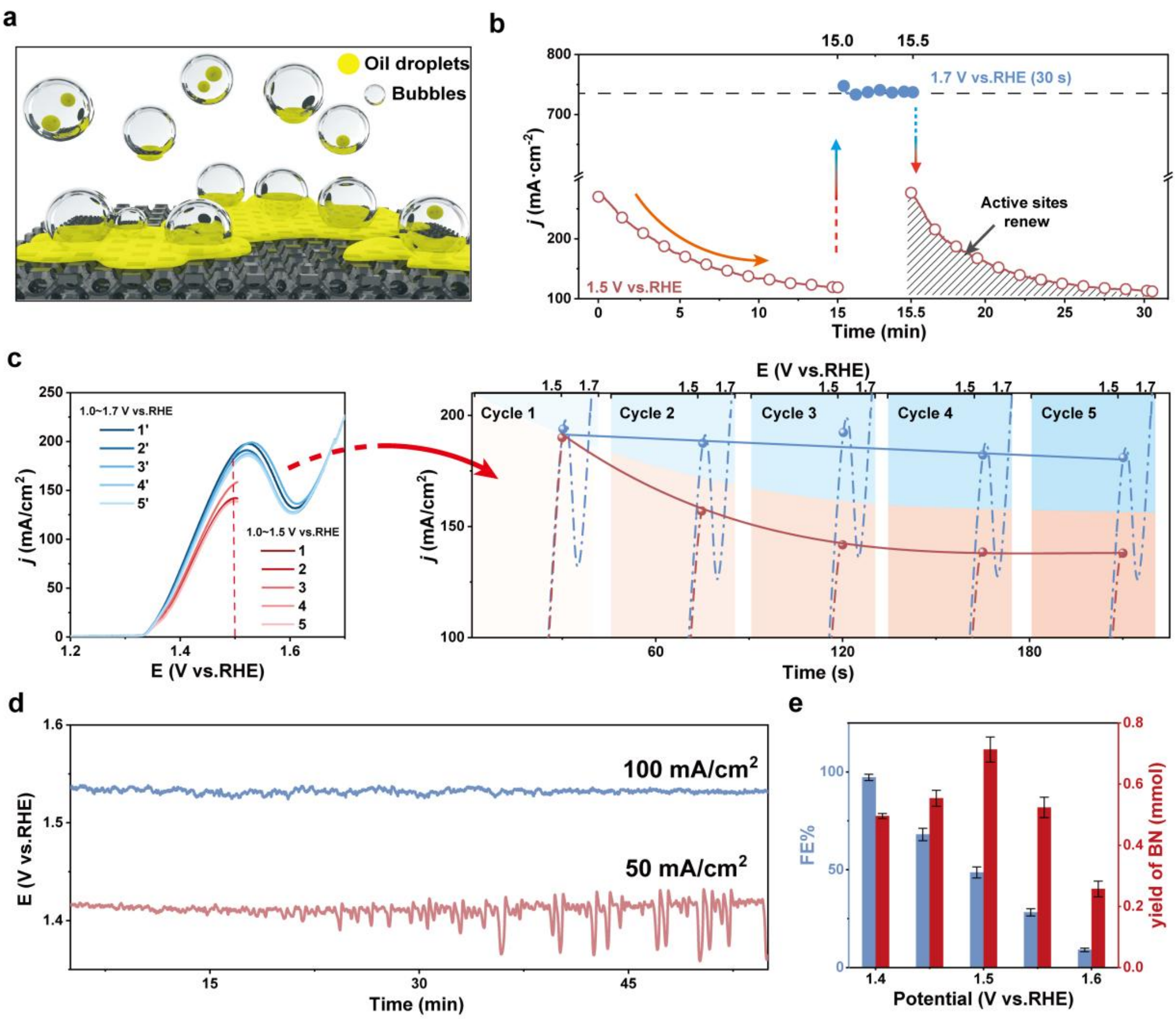

Figure 2. a) Schematic illustration of bubble-assisted removal of oily product on electrode surface. b) Surface renewal via high-potential pulse enhances current stability. c) Multi-step LSV test and current density peaks at different scan ranges with a scan rate of 10 mV/s. d) Potential variations under constant current densities of 50 mA/cm^2 and 100 mA/cm^2. e) Histogram of FE and yield under different potentials in 1 M KOH with 0.1 M BA.

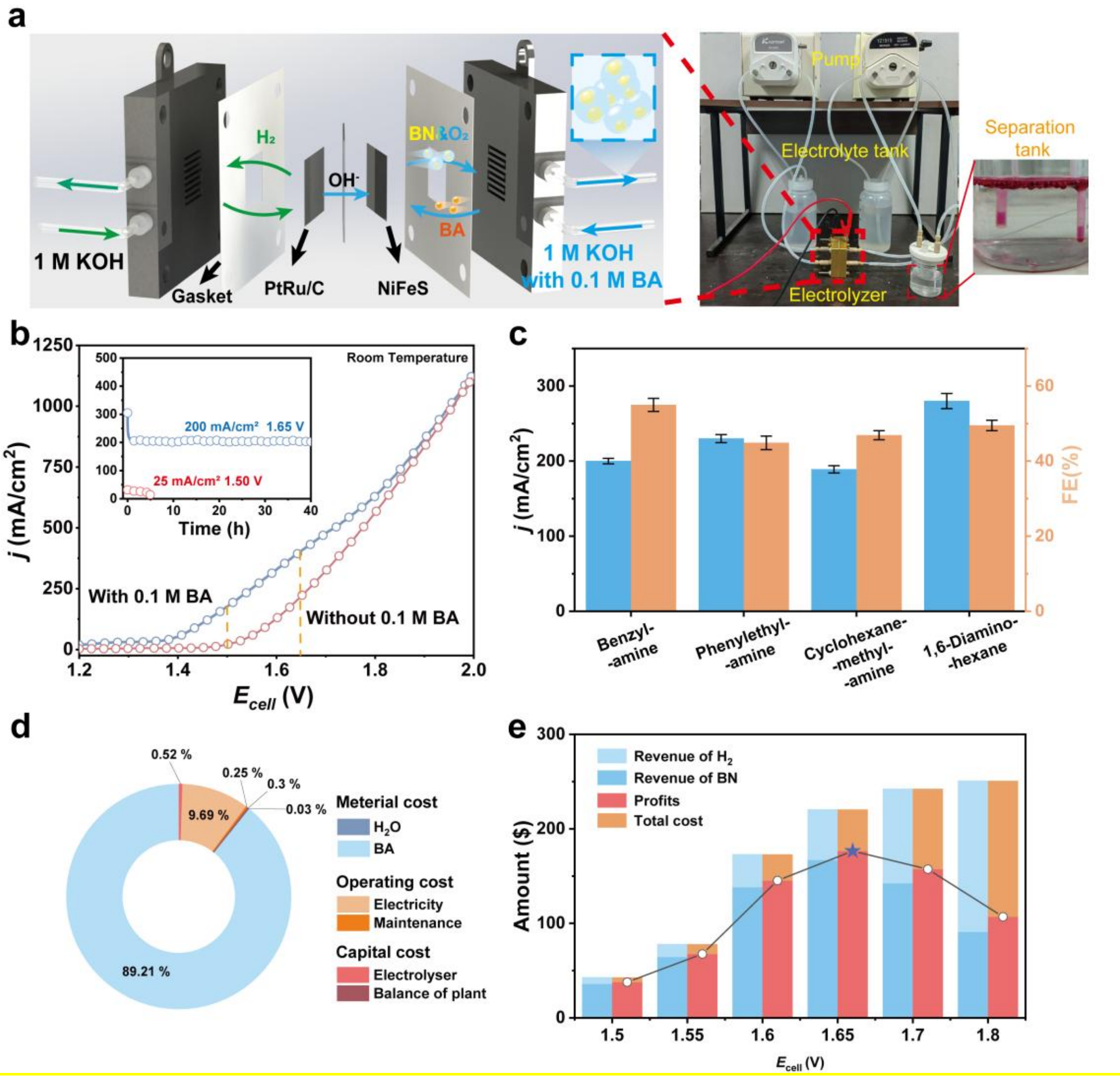

Figure 4. a) Schematic and operating schematic diagram of the AEMWE. b) LSV curves of with and without BA precursor based on a 2×2 cm^2 device. Inset: stability tests. c) Substrate scope of the electrocatalysis of primary amines into nitriles over the catalyst. d) Total cost of the BN electrochemical process at conditions of 55%, current density of 200 mA/cm^2, and cell voltage of 1.65 V. e) The revenues and profits in the different operating condition.

6. The author should evaluate the benzylamine electrooxidation performance of bare nickel foam and nickel felt.

Response: Thank you very much for the suggestion. We have evaluated the electrooxidation performance of benzylamine using bare nickel foam and nickel felt electrodes to highlight the contribution of our catalyst. The catalyst we prepared shows a sharp increase in current density, reaching 850 mA/cm^2 for 0.1 M BA at 1.42 ± 0.04 V vs. RHE, compared to 75.7 mA/cm^2 for the bare substrate.

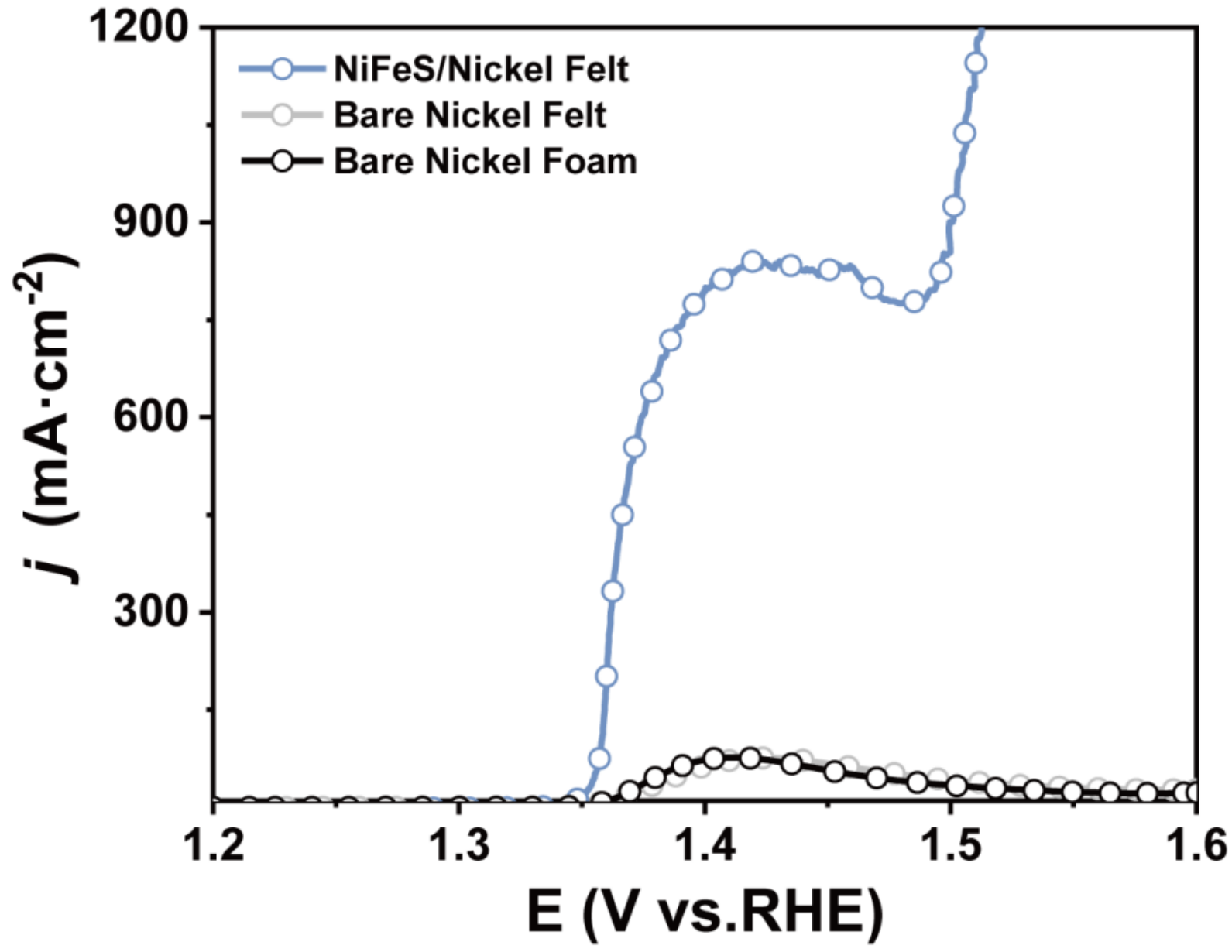

Figure R7. Polarization curves for benzylamine electrooxidation on NiFeS catalyst and bare nickel felt.

Revisions in the manuscript:

With the increase of added concentrations of BA precursor (Figure S6), the current density of polarization curve increases accordingly, and can even reach up to 850 mA/cm^2 for 0.1 M BA at 1.42 ± 0.04 V vs. RHE (corresponding to the oxidation potentials of BOR), while that of bare nickel felt or nickel foam are both only 75.7 mA/cm^2, indicating the ideal activity of NiFeS catalyst (Figure S7). Besides, the NiFeS catalyst grown on nickel foam exhibits a similar performance trend, delivering a current density of 450 mA/cm^2 in 0.1 M BA at 1.42 ± 0.04 V vs. RHE, compares to 75.0 mA/cm^2 of bare substrate (Figure S8). GC-MS analysis confirms that no detectable organic by-products, with average faradaic efficiency over 95% for BOR (Figures S9–S12).

7. It is better to investigate the HER performance when coupled with the BOR over an anion exchange membrane water electrolysis (AEMWE) device.

Response: We thank the reviewer for this valuable suggestion. To evaluate the HER performance when coupled with BOR in an anion exchange membrane water electrolysis (AEMWE) device, we have carried out additional measurements under identical conditions, as shown as Figure R8.

At a cell voltage of 1.65 V, the current density is 220 mA/cm^2 in the absence of BA (pure water electrolysis), whereas it increases to 404 mA/cm^2 upon the introduction of 0.1 M BA. This corresponds to an enhancement of approximately 83%. This significant increase in current density demonstrates that coupling HER with BOR effectively improves the overall electrolysis performance by replacing the sluggish OER with a more favorable anodic reaction.

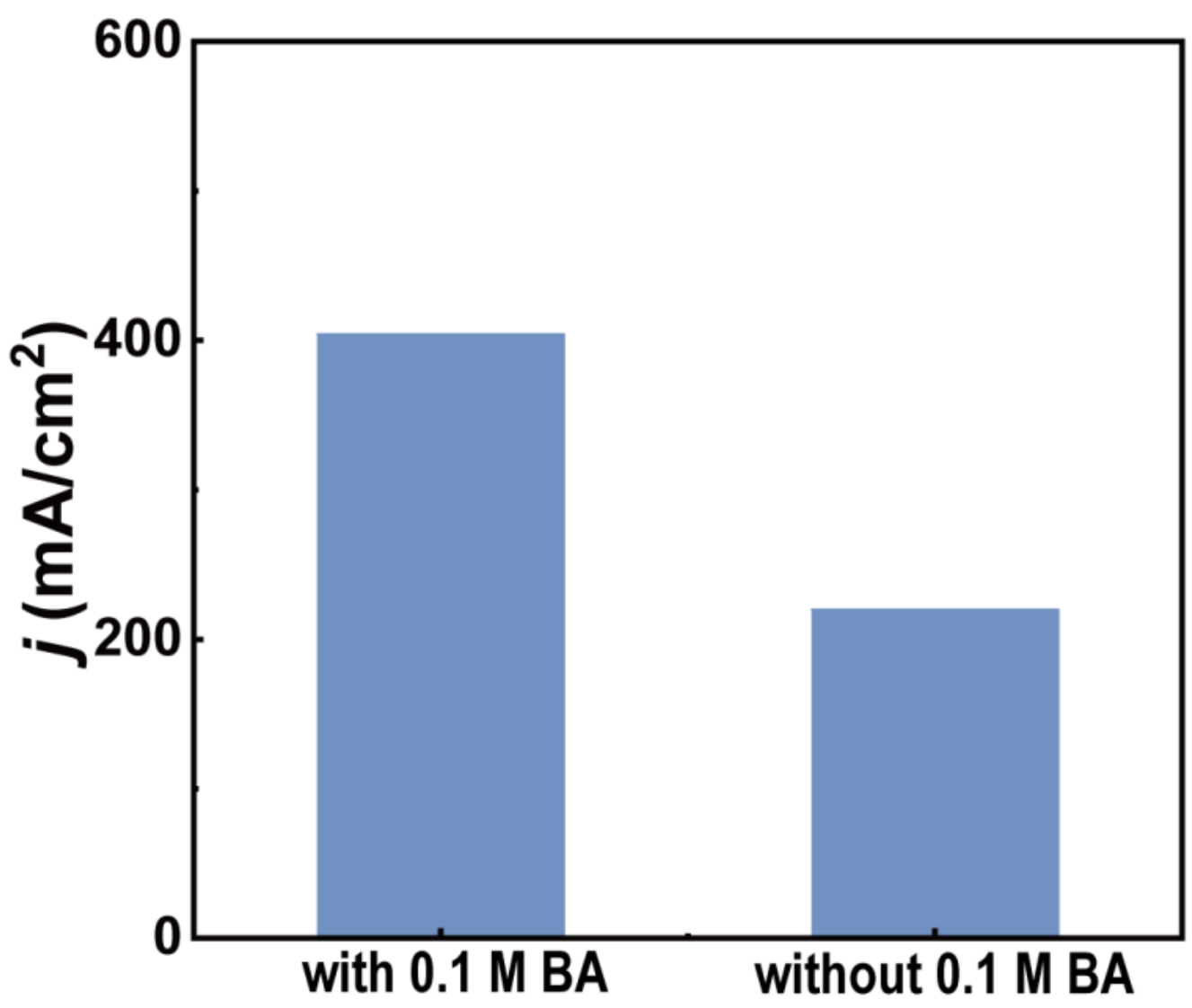

Figure R8. Comparison of current densities in AEMWE with and without benzylamine (BA) at 1.65 V.

Revisions in the manuscript:

Following the introduction of the BA solution into the organic chamber, the flow reactor promptly delivers an anodic current from approximately 1.40 V, and ultimately achieves an ampere-level current density at 1.57 V, almost twice of the current density without BA introduction, indicating a significant promotion for the hydrogen production (Figure S18).

Reviewer #3

Aiming to address the critical issues of electrode poisoning and limited current density caused by insoluble products in organic electro-oxidation coupled with hydrogen evolution reaction (HER), this study proposes a new bubble-assisted self-cleaning strategy, rather than the conventional strategy of catalyst surface modification. Taking benzylamine oxidation reaction (BOR) as the model reaction, the generated oxygen bubbles from the activated oxygen evolution

reaction (OER) are utilized to timely desorb the insoluble product benzonitrile from the electrode surface. And the constructed AEMWE achieves a high current density of 200 mA/cm^2 with stable operation for 40 hours, demonstrating both academic innovation and industrial application potential. The research features a novel design concept and a complete experimental system, integrating in-situ observation and TEA, providing a new paradigm for the design of hybrid electrolysis systems. However, the manuscript still needs improvements in mechanism elaboration and data support. Revision is recommended before acceptance, and the specific revision suggestions are as follows.

Response：

We are very grateful for the thorough and insightful review of our manuscript. We appreciate the reviewer's positive assessment of our work, particularly acknowledging the novelty of the bubble-assisted self-cleaning strategy, its potential for industrial application, and the completeness of our experimental system, including the integration of in-situ observation and TEA.

We have carefully considered all the points raised by Reviewer 3 and agree that further elaboration and data support would significantly strengthen the manuscript. We are committed to addressing each comment to the best of our ability.

1. The description in Figure 1b regarding the oil (BN) contact angle in air being close to 0°, as well as the dynamic process of Figure S4, are somewhat ambiguous as compared to the subsequent descriptions of super-hydrophilic and super-hydrophobic characteristics. The authors need to provide a clear explanation of the material properties.

Response：

We thank Reviewer 3 for highlighting the ambiguity in our initial description. We have revised the manuscript to clearly emphasize the initial super-oil-repellent property of the electrode surface underwater. This initial excellent oleophobicity

is crucial for preventing the adhesion of benzonitrile (BN) product under operating conditions.

Crucially, we also explicitly address that this initial advantageous oleophobic characteristic is not maintained during prolonged electrolysis. We explain how factors such as product accumulation or surface modification over time can lead to a decrease in this oleophobicity, ultimately contributing to electrode fouling. This dynamic change in surface properties during the reaction is a key aspect of our investigation and directly relates to the observed performance degradation. The descriptions of the text associated Figure 1b and Figure S4 have been revised.

Revisions in the manuscript:

The electrode surface can demonstrate similar wettability. The water droplet contact angle of ~0° indicates the superhydrophilicity of the electrode, and the product (BN) oil droplet contact angle presents ~0° (Figure S4). However, the underwater oil droplet contact angle exceeds 160°, presenting superoleophobic nature (Figures 1b). This initial superoleophobicity is expected for managing the organic product (Figure S5).

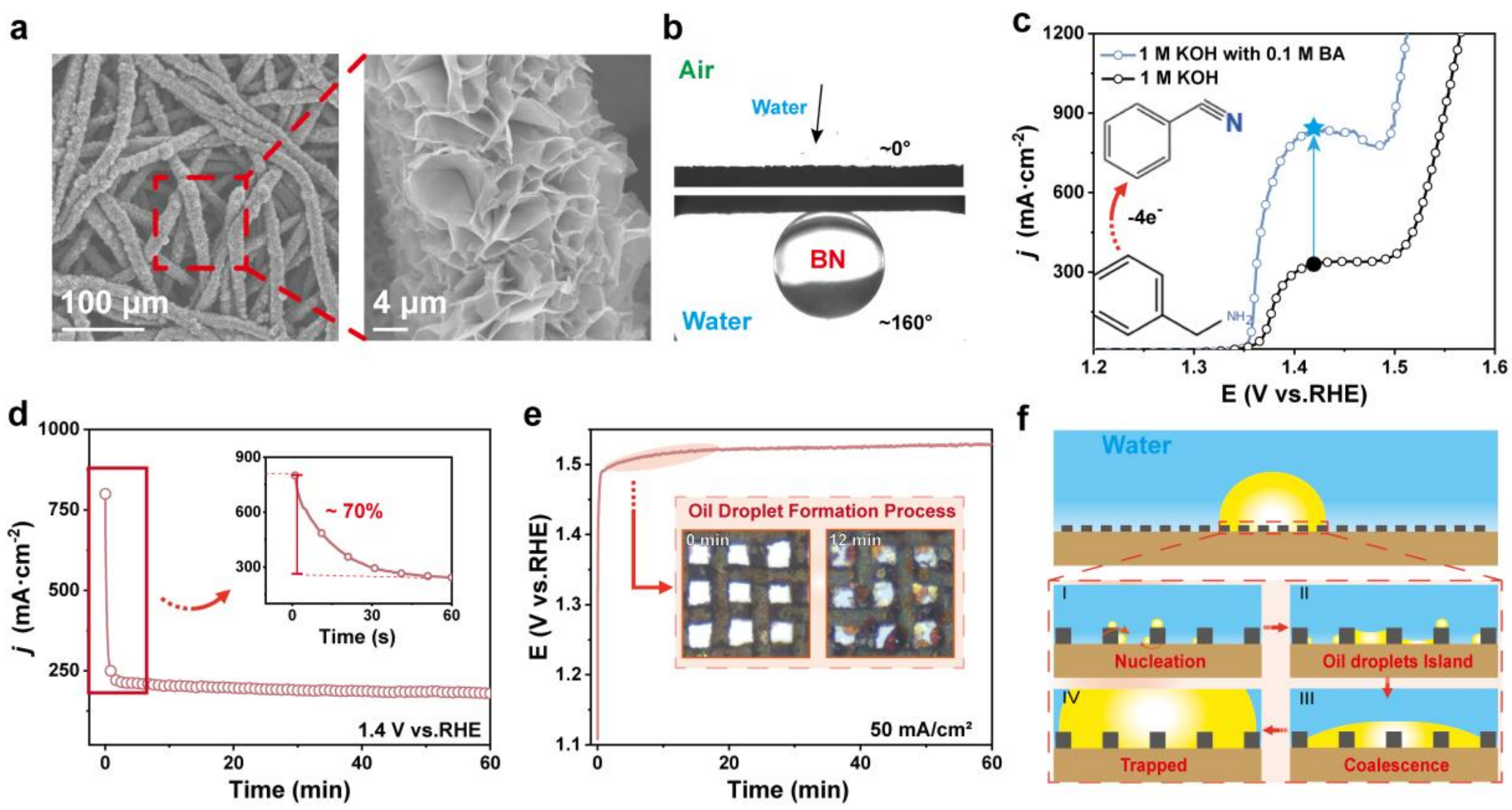

Figure 1. a) SEM images and the contact angle of NiFeS catalyst coating on nickel felt. b) The water droplet contact angle and underwater oil droplet contact angle. c) Polarization curves of NiFeS catalyst in 1 M KOH with and without 0.1 M BA. d) The stability test of NiFeS catalyst in 1 M KOH with 0.1 M BA. Inset: the decline of stability within the first 50 seconds. e) Chronoamperometry test at the current density of 50 mA/cm^2. Inset: the images of electrode surface from 0 min to 12 min. f) Schematic illustration of oil

trapped process on the electrode surface.

2. For the discussion related to Figure 2, the authors only analyzed the electrode poisoning behavior caused by insoluble organic products through the variation of current density. It is expected to provide more evidence, such as the changes in C_{dl} or the impact on ECSA.

Response:

We thank Reviewer 3 for this constructive suggestion. We agree that providing additional evidence beyond current density variations would strengthen our analysis of the electrode poisoning behavior.

To address this, we have performed Electrochemical Surface Area (ECSA) measurements, Figure R9. The ECSA data provide further quantitative support for the impact of organic product deposition on the electrode's active surface area and catalytic activity. The observed changes in ECSA correlate well with the current density variations discussed previously, reinforcing our conclusions regarding the electrode poisoning mechanism and the effectiveness of our proposed strategy.

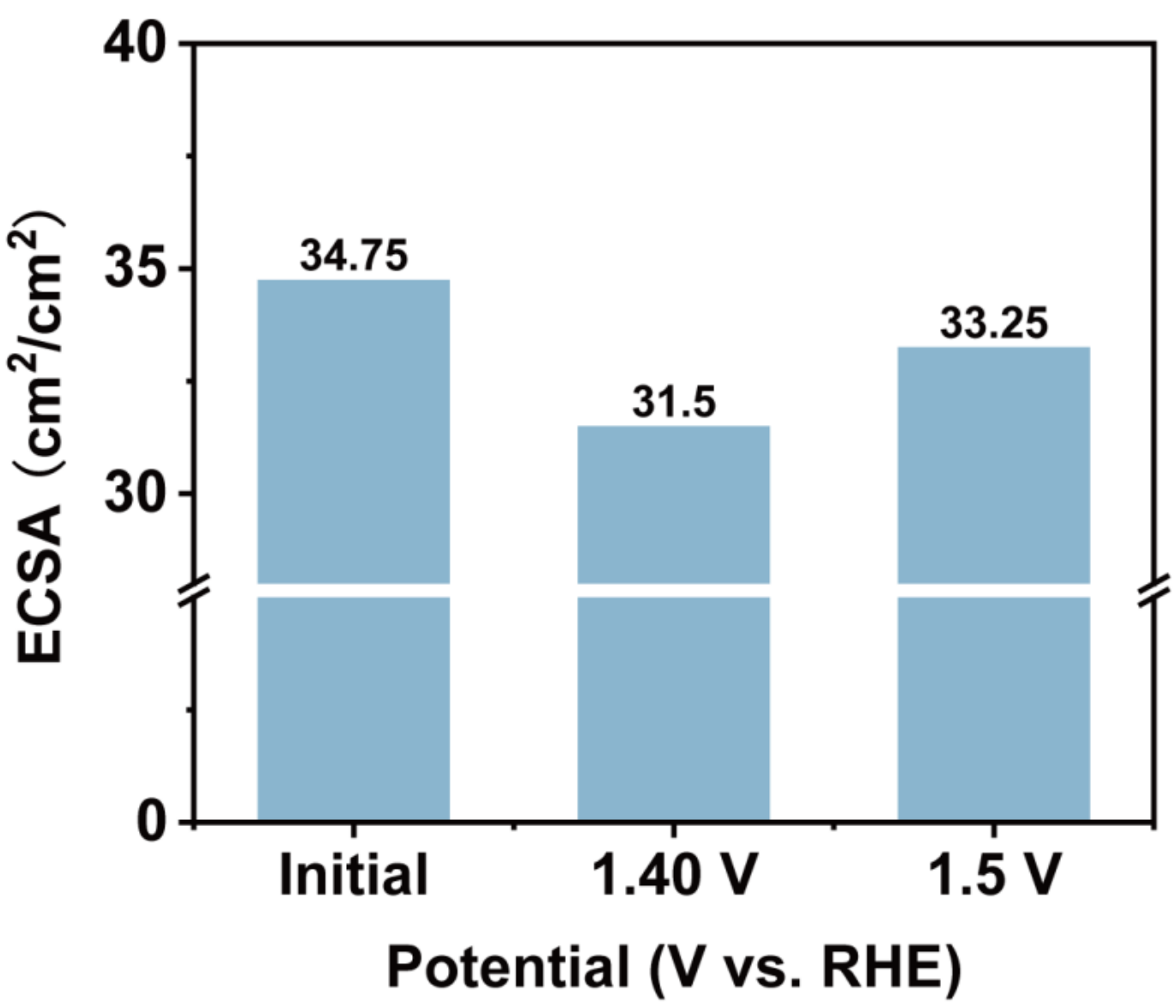

Figure R9. Electrochemical surface area (ECSA) of the electrode at different potentials

Revisions in the manuscript:

However, different from the LSV, the current density under the constant potential of 1.40 V vs. RHE decreases significantly from 800 mA/cm^2 to 230 mA/cm^2 within the first 50 seconds in each cycle, representing a decrease of over 70% (Figure 1d). To elucidate the reason of the rapid performance degradation, we investigated the electrochemical surface area (ECSA) measurements. As shown in Figure S13, the ECSA of the electrode is initially 34.75 cm^2/cm^2, but decreases to 31.5 cm^2/cm^2 after operating at 1.40 V vs. RHE for 10 min. Notably, when the reaction is conducted at a higher potential of 1.5 V vs. RHE for 10 min, the ECSA recovers to 33.25 cm^2/cm^2 on some extent.

3. In Figure 3d, the discussion on the difference of bubble coalescence with and without oil film is overbrief. An in-depth analysis needs to be conducted. There is no Figure S17.

Response: We agree that a more in-depth analysis is warranted to fully explain the observed differences between conditions with and without the oil film. We also apologize for the error in referencing Figure S17. In the revised manuscript, we expanded the discussion related to Figure 3d to provide a more thorough explanation of the role of the oil film on bubble coalescence.

Revisions in the manuscript:

Due to the strong interaction between gas and oil phases, bubbles preferentially nucleate and grow within the oil-covered regions. After rupture of the oil film, bubble coalescence proceeds through the formation and evolution of oil bridge (Figure 3d). The curvature of the oil bridge generates a Laplace pressure gradient, which governs the coalescence behavior. In the presence of an oil film, the formation of oil-mediated bridges between adjacent bubbles reduces the interfacial energy barrier and enhances the curvature gradient, thereby accelerating the coalescence process. Consequently, the oil film not only enhances bubble coalescence through dynamic Laplace pressure effects, but also promotes efficient removal of the oil layer from the electrode surface,

minimizing the probability of secondary fouling.

4. In the TEA calculation of the electrochemical reactor, there is no clear annotation of the price source for different materials. It is suggested to present the information with a table.

Response: Thank you very much for the point. We agree that clearly annotating the price sources for different materials is essential for reproducibility and clarity. To address this, we have compiled a detailed table summarizing the price sources of all materials considered in the TEA, including references and corresponding assumptions. This table has been added to the Supplementary Information of the revised manuscript.

In addition, we would like to clarify that product purity plays an important role in determining economic value. In our system, low-purity benzylamine feedstock can be selectively converted into high-purity benzonitrile, which is a higher-value product. This upgrading process further enhances the economic attractiveness of the proposed electrochemical strategy. We believe these additions improve the transparency and reliability of the TEA.

Sheet R1. Price assumptions and data sources used in the techno-economic analysis (TEA)

Item	Price (USD)	Unit	Source
Benzylamine (BA)	1.38	$/kg	*GuideChem*[1]
Benzonitrile (BN)	2.07	$/kg	*GuideChem*[1]
Hydrogen (H_2)	3	$/kg	*GuideChem*[1]
Electricity	0.05	$/kWh	*Nat. Commun.* **2024**, *15*, 9151.

[1]Price data obtained from the GuideChem database (https://china.guidechem.com), based on market quotations.

Revisions in the Supplementary Information:

Sheet S2. Price assumptions and data sources used in the techno-economic analysis (TEA)

Item	Price (USD)	Unit	Source

Benzylamine (BA)	1.38	$/kg	*GuideChem*[1]
Benzonitrile (BN)	2.07	$/kg	*GuideChem*[1]
Hydrogen (H_2)	3	$/kg	*GuideChem*[1]
Electricity	0.05	$/kWh	*Nat. Commun.* **2024**, *15*, 9151.

[1]Price data obtained from the GuideChem database (https://china.guidechem.com), based on market

Minor comments:

5. For Figure 1b, the demonstrated value of the contact angle of the underwater oil droplet is 150° while the related description is over 160°.

Response: We sincerely apologize for this careless mistake and are very grateful to the reviewer for catching it. We have carefully re-checked the original data and corrected the textual description in the revised manuscript to accurately reflect the 160° contact angle demonstrated in Figure 1b, ensuring complete consistency between the figure and the text.

Revisions in the manuscript:

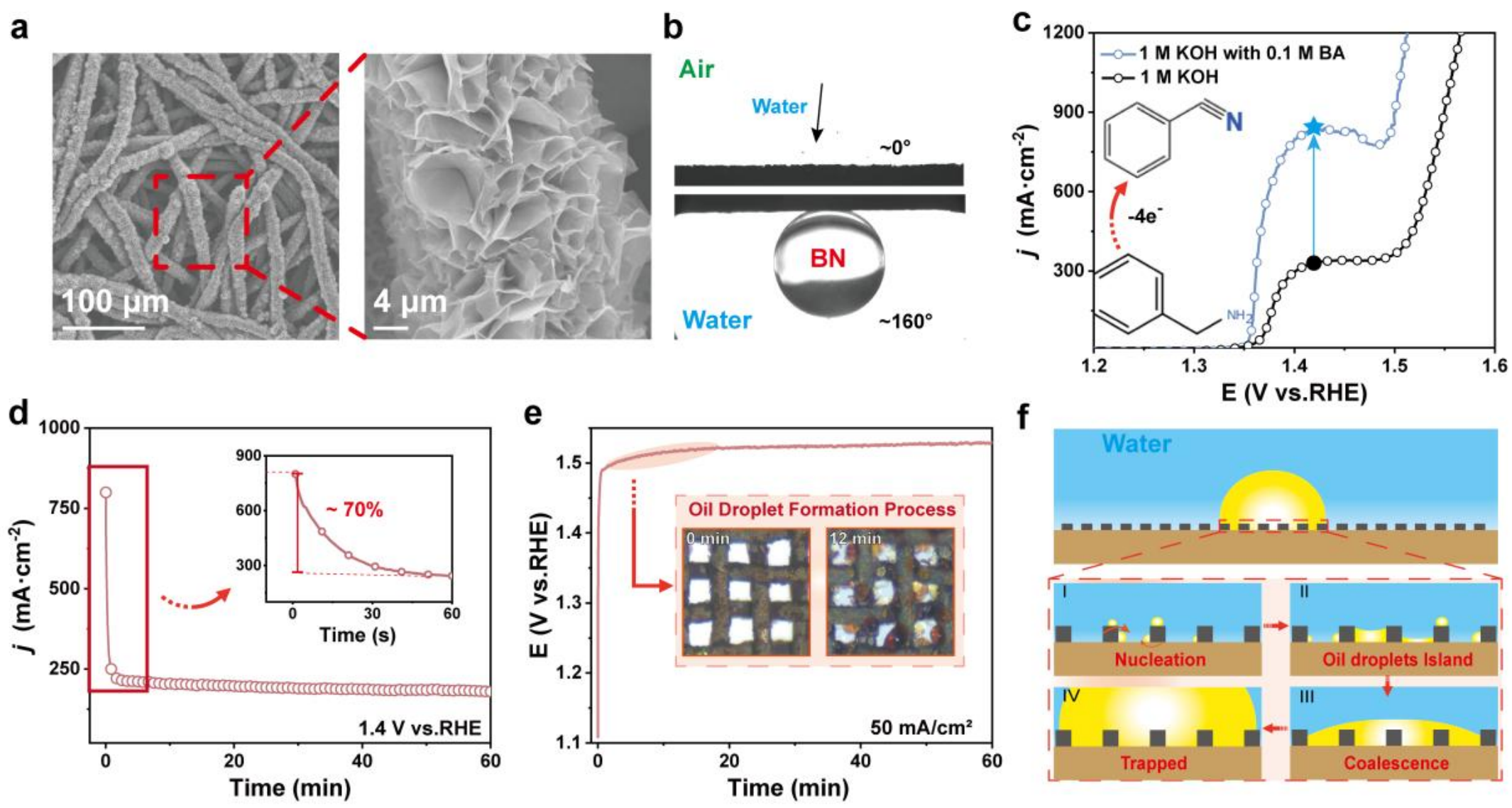

Figure 1. a) SEM images and the contact angle of NiFeS catalyst coating on nickel felt. b) The water droplet contact angle and underwater oil droplet contact angle. c) Polarization curves of NiFeS catalyst in 1 M KOH with and without 0.1 M BA. d) The stability test of NiFeS catalyst in 1 M KOH with 0.1 M BA. Inset: the decline of stability within the first 50 seconds. e) Chronoamperometry test at the current density of 50 mA/cm^2. Inset: the images of electrode surface from 0 min to 12 min. f) Schematic illustration of oil trapped process on the electrode surface.

6. There is no mention of Figure S5 in the main text.

Response:

Figure S5 is intended to provide supplementary information corresponding to Note 2 in the Supplementary Information. We have now added the appropriate reference to Figure S5 in the revised manuscript to ensure clarity and consistency.

Revisions in the manuscript:

The electrode surface can demonstrate similar wettability. The water droplet contact angle of ~0° indicates the superhydrophilicity of the electrode, and the product (BN) oil droplet contact angle presents ~0° (Figure S4). However, the underwater oil droplet contact angle exceeds 160°, presenting superoleophobic nature (Figures 1b). This initial superoleophobicity is expected for managing the organic product (Figure S5).

7. It seems that the information in Note 2 of supplementary information is not related to the GC-MS information described in the text.

Response:

We apologize for this oversight. The reviewer is correct that the previous Note 2 was not properly aligned with the GC-MS discussion in the main text.

We would like to clarify that Notes 1 and 2, together with Figure S5, are related to theoretical analysis of interfacial properties rather than product characterization. Specifically, these sections are intended to support the superhydrophilicity and underwater oleophobicity of the catalyst. Figure S5 serves as supplementary information to Notes 1 and 2 and is discussed in Note 2 accordingly.

Revisions in the manuscript:

A comparable interfacial cleaning phenomenon was noted on bare nickel mesh electrodes (Figure S17), indicating that the bubble-driven detachment mechanism is substrate-independent and primarily reliant on the physicochemical interactions between gas bubbles and oily product

(Supplementary Information Note 2).

8. The tense used in the Note 3 of supplementary information is incorrect.

Response: Thank you very much for the point. We have thoroughly proofread Note 3 in the Supplementary Information and corrected the grammatical tenses to ensure academic accuracy and readability.

Revisions in the Supplementary Information:

To evaluate the economic potential for the electrosynthesis of BN, TEA is carried out. Notably, the price of electricity, H_2O, KOH, and H_2 selected in this study is based on the recently published literature.

The anticipated service life of the electrolyzer is 20 years and the working time per year is 350 days. The specific sample for the calculation of BN production under optimistic case assumptions: Take a reactor with a fixed working area of 10 m^2 is considered as an example at a current density of 0.2 A/cm^2 with 55% FE of BN at anode and 99% of H_2 at cathode as an example:

1. The electricity price is considered to be 0.05 $/ (kW h).

2. The price of H_2O is 0.0007 $/kg. The price of KOH is 0.82 $/kg. The price of H_2 was 3 $/kg. The price of BA is 1.38 $/kg. The price of BN is 2.07 $/kg.

3. The operating voltage is set as 1.65 V.

4. The electrolyzer cost per area is calculated as follow,

$$450\ \$/kW \times 1.65\ \text{V} \times 0.2\ A/cm^2 \times 10^4 \times 10^{-3} = 1485\ \$/m^2 \quad (10)$$

5. The required total current is calculated as,

$$0.2\ A/cm^2 \times 10\ m^2 \times 1000\ cm^2/m^2 = 20000\ A \quad (11)$$

The power of BM production is calculated as follow,

$$20000\ A \times 1.65\ V \times 10^{-3} = 33\ kW \quad (12)$$

Capital cost:

Electrolyzer cost:

$$10.0\ m^2 \times 1485\ \$/m^2 \div 20\ year = 2.06\ \$ \quad (13)$$

Balance of plant:

$$2.06\ \$/ton \times 58\% = 1.20\ \$ \quad (14)$$

Operating cost:

1. Electricity:

$$33\ kW \times 24\ h\ \times 0.05\ \$ /(kW \cdot h) = 39.6\ \$ \quad (15)$$

2. Maintenance of electrolyzer:

$$10.0\ m^2 \times 1485\ \$/m^2\ \times 0.025 \div 350\ day = 1.03\ \$ \quad (16)$$

Material cost:

1. Water

$$0.0007\ \$/ton \times 18\ \mathrm{kg/mol} \times \frac{20000\ \mathrm{A} \times 99\ \% \times 24\ \mathrm{h} \times 3600\ \mathrm{s/h}}{96485 \times 2} \div 1000\ kg/ton = 0.056\ \$ \quad (17)$$

Water consumption costs are omitted from the analysis as they accounted for less than 0.1% of the total expenses.

2. BA

$$1.38\ \$/kg\ \times 107.15\ \mathrm{kg/mol} \times \frac{20000\ \mathrm{A} \times 55\ \% \times 24\ \mathrm{h} \times 3600\ \mathrm{s/h}}{96485 \times 4} = 364.74\ \$ \quad (18)$$

3. KOH

Owing to the potential for KOH recovery and reuse, it is not included in the overall cost assessment.

Total cost:

$$2.06 + 1.20\ + 39.6 + 1.03 + 364.74 = 408.63\ \$ \quad (19)$$

Revenue:

1. BN

$$1.38\ \$/kg\ \times 104.21\ \mathrm{kg/mol} \times \frac{20000\ \mathrm{A} \times 55\ \% \times 24\ \mathrm{h} \times 3600\ \mathrm{s/h}}{964854 \times 4} = 532.08\ \$ \quad (20)$$

2. H_2

$$3\ \$/kg\ \times 2\ \mathrm{kg/mol} \times \frac{20000\ \mathrm{A} \times 99\ \% \times 24\ \mathrm{h} \times 3600\ \mathrm{s/h}}{964854 \times 4} = 53.19\ \$ \quad (21)$$

Total Revenue:

$$532.08 + 53.19 = 585.27\ \$ \quad (22)$$

9. There are also several formatting issues in the manuscript, such as inconsistent annotation of current density units; incorrect annotation of electricity cost units in the TEA calculation; and ambiguous numerical expressions for income, expenditure and profit in the text.

Response:

We have carefully gone through the entire manuscript and Supplementary

Information, making the following specific corrections:

(1) Current density units: We have standardized all current density units throughout the text and figures.

(2) TEA calculation units: We have corrected the annotation of electricity cost units in the TEA calculation section to standard formats (e.g., $/kWh).

(3) Economic expressions: We have clarified and standardized the numerical expressions and formatting for income, expenditure, and profit to eliminate any ambiguity.

Revisions in the manuscript:

The hybrid device demonstrates industrially relevant performance, delivering 200 mA/cm^2 at 1.65 V with an observed 55% faradaic efficiency, and stably operates for 40 hours with essentially no decay in potential.

Based on the performance of BOR coupled AEMWE device, the techno-economic analysis (TEA) of the BOR process was further conducted, incorporating operating costs, capital costs, and material costs (Figure 4d) (see more details in Supplementary Information Note 3). The TEA results show that, the total cost and revenue are $408.63 and $585.27, respectively, indicating the profit of $176.64 with a fixed working area of 10 m^2 (current density of 200 mA/cm^2, cell voltage of 1.65 V, FE of 55%). In addition, the in-situ generated oxygen bubbles via OER serve as intrinsic bubble-assisted cleaning and oily product flotation carriers. More importantly, with the efficient bubble-assisted removal, such unique water insoluble product inversely saves the extra purification treatment and related costs, which is usually over 50% for conventional overall aqueous paired electrolysis systems.

Revisions in the Supplementary Information:

1. Electricity:

$$33\ kW \times 24\ h\ \times 0.05\ \$\ /(kW \cdot h) = 39.6\ \$ \tag{15}$$
